# Ligature-Induced Experimental Peri-Implantitis—A Systematic Review

**DOI:** 10.3390/jcm7120492

**Published:** 2018-11-28

**Authors:** David Reinedahl, Bruno Chrcanovic, Tomas Albrektsson, Pentti Tengvall, Ann Wennerberg

**Affiliations:** 1 Department of Prosthodontics, Institute of Odontology, Sahlgrenska Academy, Gothenburg University, Gothenburg 405 30, Sweden; ann.wennerberg@odontologi.gu.se; 2 Department of Prosthodontics, Faculty of Odontology, Malmö University, Malmö 205 06, Sweden; brunochrcanovic@hotmail.com (B.C.); tomas.albrektsson@biomaterials.gu.se (T.A.); 3 Department of Biomaterials, Institute of Clinical Sciences, Sahlgrenska Academy, Gothenburg University, Gothenburg 405 30, Sweden; pentti.tengvall@gu.se

**Keywords:** osseointegration, dental implant, peri-implantitis, ligature-induced peri-implantitis, aseptic loosening, systematic review

## Abstract

This systematic review sought to analyze different experimental peri-implantitis models, their potential to induce marginal bone resorption (MBR) and the necessity of bacteria for bone loss to occur in these models. An electronic search in PubMed/Medline, Web of Science, and ScienceDirect was undertaken. A total of 133 studies were analyzed. Most studies induced peri-implantitis with ligatures that had formed a biofilm, sometimes in combination with inoculation of specific bacteria but never in a sterile environment. Most vertical MBR resulted from new ligatures periodically packed above old ones, followed by periodically exchanged ligatures and ligatures that were not exchanged. Cotton ligatures produced the most MBR, followed by steel, “dental floss” (not further specified in the studies) and silk. The amount of MBR varied significantly between different animal types and implant surfaces. None of the analyzed ligature studies aimed to validate that bacteria are necessary for the inducement of MBR. It cannot be excluded that bone loss can be achieved by other factors of the model, such as an immunological reaction to the ligature itself or trauma from repeated ligature insertions. Because all the included trials allowed plaque accumulation on the ligatures, bone resorbing capacity due to other factors could not be excluded or evaluated here.

## 1. Introduction

Experiments that aimed to mimic peri-implantitis were first introduced in the early 1990s in response to reports on progressive peri-implant bone loss around dental implants [1,2]. These experiments have mainly been based on the infectious model of explanation where bacteria are the presumed cause of the phenomenon [2,3]. The view of peri-implantitis as a strictly infectious condition, as suggested in the studies these models were based on, is currently a matter of debate. Albrektsson and co-workers recently proposed that tissue responses to dental implants should be viewed similarly to other biomaterials; primarily as an immune mediated inflammatory and foreign body reaction (FBR), indicating that immunological reactions may be important also for MBR [4]. The initial, and most frequently used experimental peri-implantitis model was adopted from the ligature-induced periodontitis model. In short, ligatures made of silk, cotton, stainless-steel wire, or other materials, are placed around the neck of dental implants, usually in a sub-marginal position. The ligatures are then allowed to accumulate plaque for a few months, after which a certain amount of marginal bone resorption (MBR) can be expected. Previous authors have claimed that plaque accumulation is necessary for bone resorption to occur in response to ligation [2,5], but Baron et al. pointed out a lack of validation for this claim in their review from year 2000 and concluded that other factors, such as a foreign body reaction to the ligature may also trigger a peri-implant inflammatory response [6]. The aims of the present review were to categorize (1) the models that induce experimental peri-implantitis described in literature and (2) to verify whether the amount of vertical MBR varies between different ligature techniques (exchanged or non-exchanged ligature), material and size of ligatures, animals or implant surfaces used in these studies. With this information in mind, we then (3) critically analyzed the attempts to validate the presence of bacteria containing biofilms as a cause of MBR in ligature-induced peri-implantitis models.

## 2. Materials and Methods

The present study followed the PRISMA Statement for Transparent Reporting of Systematic Reviews and Meta-analyses [7].

### 2.1. Search Strategies

An electronic search without time restrictions for publications in English was undertaken in January 2018 in the following databases: PubMed/Medline, Web of Science, and ScienceDirect. The following terms were used in the search strategies:

(((dental implant) OR oral implant)) AND (experimentally induced periimplantitis) OR experimentally induced peri-implantitis) OR experimental periimplantitis) OR experimental peri-implantitis) OR ligature induced periimplantitis) OR ligature induced peri-implantitis) OR ligature) OR plaque induced periimplantitis) OR plaque induced peri-implantitis) OR plaque accumulation) OR mechanical overload) OR bacterial inoculation)

An additional manual search of related journals was conducted. The reference list of the identified studies and the relevant reviews on the subject were scanned for possible additional studies.

### 2.2. Inclusion and Exclusion Criteria

The inclusion criteria comprised in vivo studies performing experimentally induced peri-implantitis. All animal species used for the experiments and all methods to induce peri-implantitis were considered. Studies inducing periodontitis (around teeth) were not included, unless they were also investigating peri-implantitis around implants. Studies evaluating only mucositis around implants were excluded.

### 2.3. Study Selection

The titles and abstracts of all reports identified through the electronic searches were read independently by the authors. For studies appearing to meet the inclusion criteria, or for which there were insufficient data in the title and abstract to make a clear decision, the full report was obtained. Disagreements were resolved by discussion between the authors and the authors rapidly reached a consensus.

### 2.4. Data Extraction

Two review authors independently searched for trials aiming to validate the necessity of biofilm in order to induce MBR by means of ligatures. They then independently extracted data using specially designed data extraction forms. The data extraction forms were piloted on several papers, which were modified as required before use.

From the studies included in the final analysis, the following data was extracted (when available): Year of publication, animal species used in the experiments, number of animals, jaw receiving the implants, number of extracted teeth (to make room for the implants), implants’ healing period, study design (split design, inter-quadrant, other), implant characteristics (shape, dimensions, material, surface, bone/tissue level), number of implants per animal, number of surgery stages, time between abutment connection and peri-implantitis induction, use or not of randomization, use or not of loading, pre- and post-operative care (antibiotic, and plaque control method, timing, and duration), method of induction of peri-implantitis (ligature, overload, bacterial inoculation, other), ligature (material, size, exchange regimen, duration, control side protocol), diagnostic markers (clinical measurements, mobility, microbiological sampling, radiographies, histometric measurements, histological evaluation, other), peri-implant bone defect (time of registration, vertical MBR, horizontal MBR, measurement method, development after ligature removal, number of lost implants). Contact with authors for possible missing data was performed.

### 2.5. Analyses

Descriptive statistics were utilized to report the data. In order to standardize and clarify ambiguous data, vertical MBR was reported for each publication. Vertical MBR was the continuous outcome evaluated, and the statistical unit was the implant (the implants used in the animals). The untransformed proportion (random-effects DerSimonian–Laird (1986) method) for vertical bone loss was calculated [8] with consideration for the different implant surfaces, the different animal species, the different methods to induce peri-implantitis and, when ligature was the method used, the different regimen applied, i.e., application of only one ligature, exchange of ligatures over time, and application of a new ligature on the top of an old one. Meta-regression was performed for the outcome of vertical MBR, using the time period of peri-implantitis induction as covariate. Statistical significance was set at *p* < 0.05. The data were analyzed using the software OpenMeta (Analyst) (MetaMorph Inc., Nashville, TN, USA) [9].

## 3. Results

### 3.1. Literature Search

**Figure 1:**
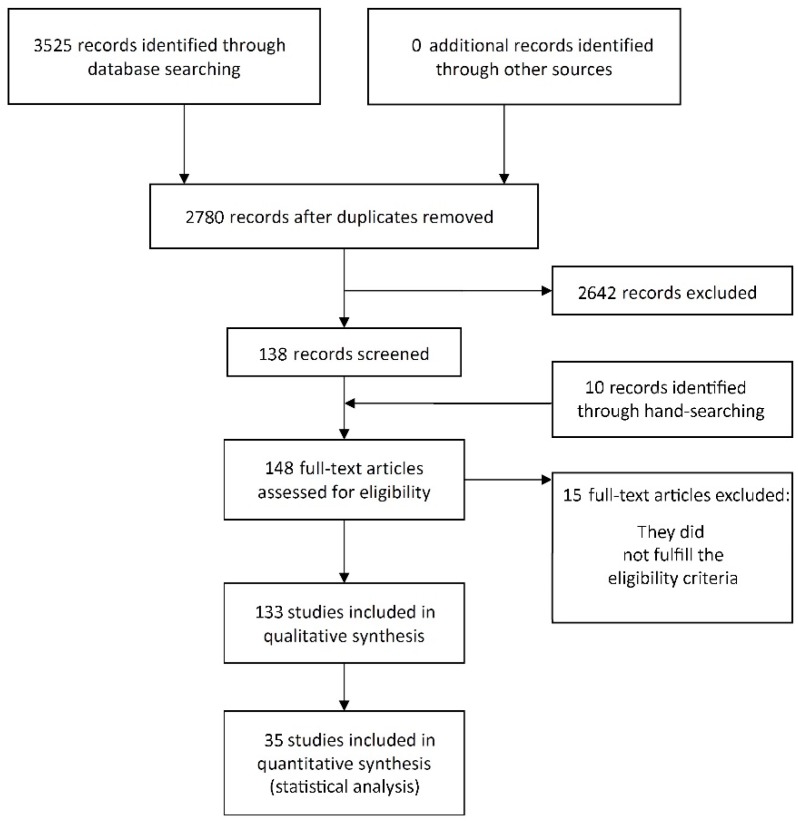
Study selection.

The study selection process is summarized in Figure 1. The search strategy in the databases returned 3525 papers. A number of 745 articles were cited in more than one database (duplicates). The reviewers independently screened the abstracts for those articles related to the aim of the review. Of the resulting 2780 studies, 2642 were excluded for not being related to the topic or for not being induced peri-implantitis animal studies. Additional hand-searching of journals and of the reference lists of selected studies yielded 10 additional papers. The full-text reports of the remaining 148 articles led to the exclusion of 15 because they did not meet the inclusion criteria. Thus, a total of 133 publications were included in the review (see References S1).

### 3.2. Description of the Studies and Analyses

Regarding ligature-induced peri-implantitis; none of the included publications attempted to validate the presence of a bacterial biofilm in order to achieve MBR.

Detailed data of the 133 included studies are listed in Tables S1 and S2. Dogs were the most commonly used animal for the experiments (being the case of 94 studies) followed by monkeys (26 studies), mice (8 studies), micro-/mini-pigs (3 studies), and rats (2 studies).

Concerning the method of peri-implantitis induction, most of the studies (*n* = 91) induced peri-implantitis with the use of ligatures alone. Ligatures of cotton were used in 50 studies, silk in 31 studies, 3 metallic ligatures, one the resorbable suture Vicryl, one “dental floss”, and 5 studies used a combination of ligatures of different materials. Thirteen studies applied a period of ligature and then a period of plaque accumulation (or vice-versa), 8 studies compared two or more groups concerning the method of peri-implantitis induction (ligature and/or overload and/or plaque accumulation), 5 studies used cotton ligatures followed by inoculation of *Porphyromonas gingivalis*. Seven studies provided a time of plaque accumulation alone, 4 studies exposed the implants to overload, 2 studies applied ligatures plus cyanoacrylate, 2 studies used bacterial inoculation alone, and 1 study immunized the animals followed by an injection of lipopolysaccharides.

The implant healing period before the induction of experimental peri-implantitis was at average 96.0 ± 57.1 days (min–max, 0–365). Seventy-nine studies used bone-level implants, 46 tissue-level implants, 5 studies used both types of implants, 2 studies installed the implants at the sub-crestal level, and proper information was not provided in one study.

The ligature methods were of three different regimens: One ligature not replaced (*n* = 53), exchange of ligatures (*n* = 43), and periodical replacement of ligatures on top of old ones (*n* = 11). Information was not available in 4 studies. Only 30 studies provided information on ligature size, varying from suture size 7–0 to 2–0 United States Pharmacopeia (USP). Other examples included 0.010 inch, 0.254 mm, and 1.58 mm. The duration of ligature placement time varied between 7 and 660 days, and some studies intercalated the use of ligatures between periods of plaque accumulation or of plaque control.

Plaque control maintenance was performed in 111 studies, where the period varied between “only once” to 2 years. Information about plaque control was not available in 8 studies. The plaque control methods used varied considerably among studies and included one or a combination of two or more of the following activities: Daily brushing with toothpaste, interdental brushing, electric pencil brush, application of chlorhexidine, scrubbing with chlorhexidine, implant scaling with plastic scaler, gentle mechanical cleaning of pockets, manual irrigation with syringe, dental water jet, flossing, and curettage. The plaque control maintenance also varied considerably among studies concerning the duration period, the number of maintenance periods, and timing (for example; after teeth extraction, after implant installation, at abutment connection, and after ligature removal).

The studies usually made use of several diagnostic markers listed in Table S2, but the present review focused on the analysis of the vertical MBR. Table 1 shows the estimated vertical bone loss in relation to implant surface type, species, method of peri-implantitis induction, and ligature regimen, according to the untransformed proportion using the DerSimonian–Laird method (1986) analysis [6]. Only studies providing the number of implants used, mean value and standard deviation for bone loss were included here (*n* = 35 of the 133 reviewed studies) because these values are necessary for the analyses. Detailed data for these 35 studies are listed in Tables S3 and S4 and a reference list is available in References S2.

## 4. Discussion

This systematic review presents a broad inclusion of experimental peri-implantitis studies that were all based on the infectious model except for a few studies that exposed the implants to overload. The infectious model of explanation for peri-implantitis stems from the traditional view that osseointegration results from ordinary bone healing adjacent to an inert implant and where all subsequent marginal bone loss is due to bacterial challenge [3,10]. Currently, it is becoming more and more clear that osseointegration is an FBR of demarcation type that is dependent on local and systemic factors [11,12,13]. Even though the demarcation type reaction serves a functional purpose by enabling dental implants to carry chewing forces, its immunological purpose is to shield off the material and limit its tissue damage. It has been well established that an FBR is initiated at the time of implantation of all biomaterials and that its expression may vary between encapsulation, dissolution, resorption or rejection, depending on the properties of the material or the trauma associated with its insertion. Its expression type may also vary over time in response to changing local or systemic factors [14,15].

Sub-marginal ligation remains the most commonly used means to induce experimental peri-implantitis and provides a relatively rapid and effective MBR. Plaque accumulation alone on the other hand, provides minimal or absent MBR as evident from the dog (*n* = 5) and monkey (*n* = 2) studies which used that technique in the present review [16,17,18,19,20,21,22]. Prior to its introduction in dental implant research in the early 1990s, the model had been used extensively in experimental periodontitis studies [23]. While it has been realized that ligature-induced peri-implantitis may not completely mimic the onset and progression of disease in humans [24], it is still generally regarded as a solely infectious model as evident from repeated claims that bone resorption results from exposition to biofilm, not the ligature per se [2,4,25]. The earliest studies on experimental peri-implantitis revealed that the infectious model of explanation was adopted from studies on native tissues (experimental periodontitis), rather than implanted material. For example, Lindhe et al. referred to a 1966 study on germ-free vs. conventional rats to support the theory [2]. In the provided reference, Rovin et al. described an increased inflammatory response in the gingival tissues of conventional rats compared to germ free rats after ligation with USP 3-0 braided silk sutures. However, none of the rats lost any bone and neither did the distance between the epithelial attachment and cemento-enamel junction vary between these groups at any time interval until the termination of the experiment after 6 months of ligation. Rovin et al. concluded that their specific rat type appeared unsusceptible to periodontitis [26]. We failed to identify any experimental peri-implantitis studies that attempted to validate that ligatures cannot induce MBR without the presence of a biofilm. As concluded in a previous review by Baron et al., it still “remains uncertain whether the peri-implant inflammatory response is really only the result of increased plaque accumulation or if the thread itself, as a foreign body, also acts as a stimulus” [5]. 

In the present review dogs displayed more bone loss in response to ligation than monkeys, which points to differences in their susceptibility to ligature-induced peri-implantitis. The time required to achieve a certain amount of bone loss also varied greatly within the groups of dogs or monkeys in single studies [27,28]. The difference in MBR between rodents and large animals in the present review may be due to the small implants and ligatures used in rodents compared to dogs and monkeys. One must also consider the small number of rodent studies (*n* = 2) included in the analysis. Recently, different mouse strains and knock out models have been used to better understand why the marginal bone resorption in response to ligation differs between animals [29,30].

The significant differences in the MBR that resulted from different ligature materials in the present meta-analysis, may be due to differences between the FBRs provoked by these materials. They could also be related to varying plaque carriage potentials for these materials, but this can hardly explain why smooth stainless-steel ligatures induced more bone loss than twined silk. Consider also the significant differences in MBR between different ligature regimens, with more bone loss generated from regularly exchanged ligatures and new ligatures packed on top of old ones, as compared to only one ligature. Studies that utilized the exchanged ligature technique in the present review typically carried out the exchange every 3 weeks by first removing the old ligature and then pushing a new one into the bottom of the existing peri-implant pocket. Assuming that an anaerobic flora exists in the apical part of a lesion prior to ligature exchange, it is likely that the removal procedure will disrupt the biofilm and also introduce air into the lesion (aeration is generally considered an important part of the surgical treatment of an anaerobic infection). It would also take some time for a new anaerobic biofilm to develop on the new, presumably clean ligature. This may indicate that a close proximity between ligature and bone, is more critical for continuous bone loss to occur than the type and composition of its bacterial flora. The general disregard of the ligature itself as a potential inducer of bone loss may perhaps explain why details about ligature size, material, structure, and sometimes composition, were left out in many studies. In fact, none of the included studies compared the effect of different ligature types or regimens.

The addition of ligature around a dental implant introduces a new foreign body on another foreign body. It is plausible to assume that the new foreign body will alter the biological response of adjacent tissues to a type more specific for the introduced ligature material. Because bacteria were always present on the ligatures of the included studies, their isolated effect could not be evaluated in the present review. However, the FBRs to some of the most common ligature materials have been described elsewhere in the literature. Silk, for example, is commonly used in in vivo applications and produced the least amount of marginal bone loss in the present review. Setzen et al. reported that twined silk sutures, identical with those commonly used in the trials of the present review, produced a relatively extensive foreign body reaction and the thickest fibrous capsule of 11 sterile suture materials that were placed subcutaneously in a rabbit model [31]. Cotton on the other hand, has been shown to induce intense FBRs and also produced the most vertical MBL in the present review. Even though cotton is generally avoided in vivo, it has sometimes been used as a negative control in studies that compared the FBR to different biomaterials [32]. From reports on iatrogenic injuries following surgery, it is known that retained sterile cotton sponges can cause large lesions of foreign body type in both hard and soft tissues, sometimes referred to as “cottonballomas”. For example, Kalbermatten et al. reported a massive osteolytic process surrounded by a thin rim of bone next to a sterile cotton sponge that had been left 25 years earlier during the stabilization of a femoral fracture [33]. The case exemplifies the slow progression often seen in foreign body type lesions, as well as their potential to induce both bone resorption and bone remodelling. Even microscopic remnants of sterile lint retained from cotton sponges during routine surgery have been shown to cause considerable foreign body granulomas with ingested lint particles visible within macrophages and foreign body giant cells after 4 weeks in rats [34]. It is plausible that similar particles can disseminate from the cotton ligatures used to induce experimental peri-implantitis, but their presence or eventual role does not appear to have been investigated in experimental peri-implantitis models. Within the orthopedic field, macrophage activation after phagocytosis or cell-surface contact with wear particles is considered the key causal reason for aseptic implant loosening, which is the main cause for revision surgery over the mid- and long-term [35,36].

The addition of bacteria on ligatures, as was the case in all reviewed studies, will obviously alter the inflammatory reaction to the ligature and likely plays an important role in the initiation and progression of MBR, i.e. it is an offer to bacteria to invade new space. Bacteria may however not be the only cause, as indicated by the results of this meta-analysis and other cited literature. It therefore remains uncertain whether a ligature-induced lesion represents clinical peri-implantitis better than a man-made defect would, perhaps created at implant insertion and then allowed to accumulate biofilm for a short time. A man-made defect would probably decrease animal suffering significantly, considering that ligatures have sometimes been left in place for as long as 1 year to induce a few millimeters of bone loss [37,38,39]. A few experiments with man-made defects have been reported. For example, Takasaki et al. combined manually created bone defects with a shorter ligature period of 5 weeks [40]. 

It also remains uncertain as to what extent a ligature-induced lesion can represent clinical peri-implantitis and until further validation has been provided, clinical conclusions from ligature-induced peri-implantitis studies should be drawn with great care and the experimental and clinical conditions should be treated as separate entities at all times.

## Figures and Tables

**Table 1:** Estimated vertical bone resorption in relation to implant surface type, species, method of peri-implantitis induction and ligature regimen.

Factor	MBR (in mm) (95% CI), *p*-Value, SE	Heterogeneity	Number of Measurements * Included for the Meta Regression (Number of Studies)
**Surface**			
Turned	2.265 (1.786, 2.745), *p* < 0.001, 0.245	*I*^2^ = 99.5%, *p* < 0.001	29 (21)
Acid-etched	2.864 (2.491, 3.237), *p* < 0.001, 0.190	*I*^2^ = 94.72%, *p* < 0.001	12 (6)
SB	2.509 (1.555, 3.463), *p* < 0.001, 0.487	*I*^2^ = 98.03%, *p* < 0.001	6 (5)
SB+F	1.697 (-0.640, 4.034), *p* = 0.155, 1.193	*I*^2^ = 98.94%, *p* < 0.001	3 (2)
SBAE	2.175 (1.658, 2.693), *p* < 0.001, 0.264	*I*^2^ = 98.44%, *p* < 0.001	21 (10)
SBAE/HA-coated	2.700 (2.174, 3.226), NA, 0.268	NA	1 (1)
HA-coated	2.349 (1.254, 3.444), *p* < 0.001, 0.559	*I*^2^ = 98.58%, *p* < 0.001	12 (7)
HA-plasma	1.650 (1.410, 1.890), *p* < 0.001, 0.122	*I*^2^ = 0%, *p* = 0.683	2 (1)
CaP-plasma sprayed	0.469 (-0.010, 0.949), *p* = 0.055, 0.245	*I*^2^ = 75.67%, *p* < 0.043	2 (1)
TPS	2.184 (1.523, 2.844), *p* < 0.001, 0.337	*I*^2^ = 98.16%, *p* < 0.001	13 (9)
Cancellous	1.932 (1.432, 2.431), *p* < 0.001, 0.255	*I*^2^ = 0%, *p* = 0.990	4 (1)
Anodized	3.462 (3.273, 3.651), *p* < 0.001, 0.097	*I*^2^ = 16.1%, *p* = 0.311	4 (2)
**Overall**	**2.295 (2.042, 2.548), *p* < 0.001, 0.129**	***I*^2^ = 99.18%, *p* < 0.001**	109 (35 **)
**Species**			
Dog	2.389 (2.152, 2.626), *p* < 0.001, 0.121	*I*^2^ = 98.3%, *p* < 0.001	98 (30)
Monkey	1.649 (1.254, 2.044), *p* < 0.001, 0.202	*I*^2^ = 96.75%, *p* < 0.001	9 (4)
Rat	0.800 (0.393, 1.207), NA, 0.208	NA	1 (1)
Mouse	0.579 (0.549, 0.609), NA, 0.015	NA	1 (1)
**Overall**	**2.295 (2.042, 2.548), *p* < 0.001, 0.129**	***I*^2^ = 99.18%, *p* < 0.001**	109 (35 **)
**Method**			
Plaque	0.689 (0.507, 0.871), *p* < 0.001, 0.093	*I*^2^ = 66.17%, *p* = 0.007	7 (2)
Inoculation	0.800 (0.393, 1.207), NA, 0.208	NA	1 (1)
**Ligature**			
Cotton	2.730 (2.478, 2.982), *p* < 0.001, 0.129	*I*^2^ = 97.88%, *p* < 0.001	66 (22)
Silk	1.683 (1.296, 2.070), *p* < 0.001, 0.197	*I*^2^ = 98.98%, *p* < 0.001	28 (8)
“Dental floss”	2.361 (1.675, 3.046), *p* < 0.001, 0.350	*I*^2^ = 86.05%, *p* = 0.007	2 (1)
Steel	2.607 (1.359, 3.856), *p* < 0.001, 0.637	*I*^2^ = 98.08%, *p* < 0.001	5 (2)
**Overall**	**2.295 (2.042, 2.548), *p* < 0.001, 0.129**	***I*^2^ = 99.18%, *p* < 0.001**	109 (35 **)
**Ligature regimen**			
Only one	2.000 (1.647, 2.352), *p* < 0.001, 0.180	*I*^2^ = 98.91%, *p* < 0.001	37 (13)
Exchange	2.303 (2.030, 2.576), *p* < 0.001, 0.139	*I*^2^ = 97.89%, *p* < 0.001	48 (14)
New on top	3.123 (2.409, 3.838), *p* < 0.001, 0.365	*I*^2^ = 98.66%, *p* < 0.001	20 (6)
**Overall**	**2.395 (2.098, 2.620), *p* < 0.001, 0.133**	***I*^2^ = 99.2%, *p* < 0.001**	105 (33) ***

CI—confidence interval, SE—standard error, MBL—marginal bone loss, SB—sandblasted, SB+F—sandblasted + fluoride modified, SBAE—Sandblasted/acid-etched, HA—hydroxyapatite, NA—not applicable (there is only one study for this category), TPS—Titanium plasma-sprayed. * Some studies performed measurements of MBL in different implant surfaces, and/or different time points, and/or different methods. ** The total number of studies is always 35 for the factors “surface”, “species”, and “method”, even though the sum of the studies for all categories under the same factor is higher than 35. The reason for this is that some studies performed measurements of MBL in different conditions, as explained in the footnote “*” above. An exception is made for “ligature regimen” (see footnote “***” below). *** The ligature regimen was not clearly informed in four measurements performed in two studies.

## Supplementary Materials

The following are available online at http://www.mdpi.com/2077-0383/7/12/492/s1, Table S1: Main characteristics of the studies included in the qualitative synthesis; Table S2: Induction and outcome of experimentally induced peri-implant bone loss in studies included in the qualitative synthesis; Table S3: Main characteristics of the studies included in the quantitative synthesis; Table S4: Induction and outcome of experimentally induced peri-implant bone loss in studies included in the quantitative synthesis; References S1: List of 133 studies included in the qualitative synthesis and excluded full text studies; References S2: List of 35 studies included in the quantitative synthesis.
