# Supplementary material for: Ligature-Induced Experimental Peri-Implantitis—A Systematic Review
Source: J Clin Med. 2018 Nov 28;7(12):492. doi: 10.3390/jcm7120492 (PMC6306937; doi:10.3390/jcm7120492)
Supplement: Supplementary file 1 [file jcm-07-00492-s001.zip › Reference SI and S2.pdf]

Reference S1: list of 133 studies included in the qualitative synthesis and excluded full text studies

a. Publications included in the review;
b. Publications excluded from the review.

a. Publications included in the review

The 133 publications included in the review are listed below.

1. Abrahamsson I, Berglundh T, Lindhe J. Soft tissue response to plaque formation at different implant systems. A comparative study in the dog. Clin Oral Implants Res. 1998;9(2):73-9.
2. Akagawa Y, Matsumoto T, Hashimoto M, Tsuru H. Clinical evaluation of the gingiva around single crystal sapphire endosseous implant after experimental ligature-induced plaque accumulation in monkeys. J Prosthet Dent. 1992;68(1):111-5.
3. Akagawa Y, Matsumoto T, Kawamura M, Tsuru H. Changes of subgingival microflora around single-crystal sapphire endosseous implants after experimental ligature-induced plaque accumulation in monkeys. J Prosthet Dent. 1993;69(6):594-8.
4. Albouy JP, Abrahamsson I, Berglundh T. Spontaneous progression of experimental peri-implantitis at implants with different surface characteristics: an experimental study in dogs. J Clin Periodontol. 2012;39(2):182-7.
5. Albouy JP, Abrahamsson I, Persson LG, Berglundh T. Implant surface characteristics influence the outcome of treatment of peri-implantitis: an experimental study in dogs. J Clin Periodontol. 2011;38(1):58-64.
6. Albouy JP, Abrahamsson I, Persson LG, Berglundh T. Spontaneous progression of ligatured induced peri-implantitis at implants with different surface characteristics. An experimental study in dogs II: histological observations. Clin Oral Implants Res. 2009;20(4):366-71.
7. Albouy JP, Abrahamsson I, Persson LG, Berglundh T. Spontaneous progression of peri-implantitis at different types of implants. An experimental study in dogs. I: clinical and radiographic observations. Clin Oral Implants Res. 2008;19(10):997-1002.
8. Battula S, Lee JW, Wen HB, Papanicolaou S, Collins M, Romanos GE. Evaluation of Different Implant Designs in a Ligature-Induced Peri-implantitis Model: A Canine Study. Int J Oral Maxillofac Implants. 2015;30(3):534-45.
9. Becker ST, Föge M, Beck-Broichsitter BE, Gavrilova O, Bolte H, Rosenstiel P, Wiltfang J. Induction of periimplantitis in dental implants. J Craniofac Surg. 2013;24(1):e15-8.
10. Berglundh T, Gotfredsen K, Zitzmann NU, Lang NP, Lindhe J. Spontaneous progression of ligature induced peri-implantitis at implants with different surface roughness: an experimental study in dogs. Clin Oral Implants Res. 2007;18(5):655-61.
11. Carcuac O, Abrahamsson I, Albouy JP, Linder E, Larsson L, Berglundh T. Experimental periodontitis and peri-implantitis in dogs. Clin Oral Implants Res. 2013;24(4):363-71.
12. Carcuac O, Abrahamsson I, Charalampakis G, Berglundh T. The effect of the local use of chlorhexidine in surgical treatment of experimental peri-implantitis in dogs. J Clin Periodontol. 2015;42(2):196-203.
13. Charalampakis G, Abrahamsson I, Carcuac O, Dahlén G, Berglundh T. Microbiota in experimental periodontitis and peri-implantitis in dogs. Clin Oral Implants Res. 2014;25(9):1094-8.
14. Comut AA, Weber HP, Shortkroff S, Cui FZ, Spector M. Connective tissue orientation around dental implants in a canine model. Clin Oral Implants Res. 2001;12(5):433-40.
15. Cook SD, Rust-Dawicki AM. In vivo evaluation of CSTi dental implants in the presence of ligature-induced peri-implantitis. J Oral Implantol. 1995;21(3):191-200.
16. Deppe H, Greim H, Brill T, Wagenpfeil S. Titanium deposition after peri-implant care with the carbon dioxide laser. Int J Oral Maxillofac Implants. 2002;17(5):707-14.
17. Deppe H, Horch HH, Henke J, Donath K. Peri-implant care of ailing implants with the carbon dioxide laser. Int J Oral Maxillofac Implants. 2001;16(5):659-67.
18. Deppe H, Wagenpfeil S, Donath K. Comparative value of attachment measurements in implant dentistry. Int J Oral Maxillofac Implants. 2004;19(2):208-15.
19. Eke PI, Braswell LD, Fritz ME. Microbiota associated with experimental peri-implantitis and periodontitis in adult Macaca mulatta monkeys. J Periodontol. 1998;69(2):190-4.

20. Ericsson I, Berglundh T, Marinello C, Liljenberg B, Lindhe J. Long-standing plaque and gingivitis at implants and teeth in the dog. Clin Oral Implants Res. 1992;3(3):99-103.
21. Ericsson I, Persson LG, Berglundh T, Edlund T, Lindhe J. The effect of antimicrobial therapy on periimplantitis lesions. An experimental study in the dog. Clin Oral Implants Res. 1996;7(4):320-8.
22. Ericsson I, Persson LG, Berglundh T, Marinello CP, Lindhe J, Klinge B. Different types of inflammatory reactions in peri-implant soft tissues. J Clin Periodontol. 1995;22(3):255-61.
23. Fan X, Wang Z, Ji P, Bian Y, Lan J. rgpA DNA vaccine induces antibody response and prevents alveolar bone loss in experimental peri-implantitis. J Periodontol. 2013;84(6):850-6.
24. Fickl S, Kebschull M, Calvo-Guirado JL, Hürzeler M, Zuhr O. Experimental Peri-Implantitis around Different Types of Implants - A Clinical and Radiographic Study in Dogs. Clin Implant Dent Relat Res. 2015;17 Suppl 2:e661-9.
25. Fritz ME, Braswell LD, Koth D, Jeffcoat M, Reddy M, Cotsonis G. Experimental peri-implantitis in consecutively placed, loaded root-form and plate-form implants in adult Macaca mulatta monkeys. J Periodontol. 1997;68(11):1131-5.
26. Godoy-Gallardo M, Manzanares-Céspedes MC, Sevilla P, Nart J, Manzanares N, Manero JM, Gil FJ, Boyd SK, Rodríguez D. Evaluation of bone loss in antibacterial coated dental implants: An experimental study in dogs. Mater Sci Eng C Mater Biol Appl. 2016;69(1):538-45.
27. Golubovic V, Mihatovic I, Becker J, Schwarz F. Accuracy of cone-beam computed tomography to assess the configuration and extent of ligature-induced peri-implantitis defects. A pilot study. Oral Maxillofac Surg. 2012;16(4):349-54.
28. Gotfredsen K, Berglundh T, Lindhe J. Bone reactions at implants subjected to experimental peri-implantitis and static load. A study in the dog. J Clin Periodontol. 2002;29(2):144-51.
29. Grunder U, Hürzeler MB, Schüpbach P, Strub JR. Treatment of ligature-induced peri-implantitis using guided tissue regeneration: a clinical and histologic study in the beagle dog. Int J Oral Maxillofac Implants. 1993;8(3):282-93.
30. Guo M, Wang Z, Fan X, Bian Y, Wang T, Zhu L, Lan J. kgp, rgpA, and rgpB DNA vaccines induce antibody responses in experimental peri-implantitis. J Periodontol. 2014;85(11):1575-81.
31. Hanisch O, Cortella CA, Boskovic MM, James RA, Slots J, Wikesjö UM. Experimental peri-implant tissue breakdown around hydroxyapatite-coated implants. J Periodontol. 1997;68(1):59-66.
32. Hayek RR, Araújo NS, Gioso MA, Ferreira J, Baptista-Sobrinho CA, Yamada AM, Ribeiro MS. Comparative study between the effects of photodynamic therapy and conventional therapy on microbial reduction in ligature-induced peri-implantitis in dogs. J Periodontol. 2005;76(8):1275-81.
33. Hickey JS, O'Neal RB, Scheidt MJ, Strong SL, Turgeon D, Van Dyke TE. Microbiologic characterization of ligature-induced peri-implantitis in the microswine model. J Periodontol. 1991;62(9):548-53.
34. Hiyari S, Naghibi A, Wong R, Sadreshkevary R, Yi-Ling L, Tetradis S, Camargo PM, Pirih FQ. Susceptibility of different mouse strains to peri-implantitis. J Periodontal Res. 2018;53(1):107-116.
35. Hiyari S, Wong RL, Yaghsezian A, Naghibi A, Tetradis S, Camargo PM, Pirih FQ. Ligature-induced peri-implantitis and periodontitis in mice. J Clin Periodontol. 2018;45(1):89-99.
36. Htet M, Madi M, Zakaria O, Miyahara T, Xin W, Lin Z, Aoki K, Kasugai S. Decontamination of Anodized Implant Surface with Different Modalities for Peri-Implantitis Treatment: Lasers and Mechanical Debridement with Citric Acid. J Periodontol. 2016 Mar 11:1-17. [Epub ahead of print]
37. Huang B, Piao M, Zhang L, Wang X, Xu L, Zhu W, Meng H. Ligature-induced peri-implant infection in crestal and subcrestal implants: a clinical and radiographic study in dogs. PeerJ. 2015 Jul 30;3:e1139.
38. Huang B, Zhang L, Xu L, Zhu W, Witek L, Tovar N, Coelho PG, Meng H. Effect of implant placement depth on the peri-implant bone defect configurations in ligature-induced peri-implantitis: An experimental study in dogs. Med Oral Patol Oral Cir Bucal. 2018;23(1):e30-e37
39. Hürzeler MB, Quiñones CR, Kohal RJ, Rohde M, Strub JR, Teuscher U, Caffesse RG. Changes in peri-implant tissues subjected to orthodontic forces and ligature breakdown in monkeys. J Periodontol. 1998;69(3):396-404.
40. Hürzeler MB, Quiñones CR, Morrison EC, Caffesse RG. Treatment of peri-implantitis using guided bone regeneration and bone grafts, alone or in combination, in beagle dogs. Part 1: Clinical findings and histologic observations. Int J Oral Maxillofac Implants. 1995;10(4):474-84.

41. Hürzeler MB, Quiñones CR, Schüpback P, Morrison EC, Caffesse RG. Treatment of peri-implantitis using guided bone regeneration and bone grafts, alone or in combination, in beagle dogs. Part 2: Histologic findings. Int J Oral Maxillofac Implants. 1997;12(2):168-75.
42. Ikumi N, Suzawa T, Yoshimura K, Kamijo R. Bone Response to Static Compressive Stress at Bone-Implant Interface: A Pilot Study of Critical Static Compressive Stress. Int J Oral Maxillofac Implants. 2015;30(4):827-33.
43. Ishii K, Matsuo M, Hoshi N, Takahashi SS, Kawamata R, Kimoto K. Effect of Ultraviolet Irradiation of the Implant Surface on Progression of Periimplantitis-A Pilot Study in Dogs. Implant Dent. 2016;25(1):47-53.
44. Isidor F. Clinical probing and radiographic assessment in relation to the histologic bone level at oral implants in monkeys. Clin Oral Implants Res. 1997;8(4):255-64.
45. Isidor F. Histological evaluation of peri-implant bone at implants subjected to occlusal overload or plaque accumulation. Clin Oral Implants Res. 1997;8(1):1-9.
46. Isidor F. Loss of osseointegration caused by occlusal load of oral implants. A clinical and radiographic study in monkeys. Clin Oral Implants Res. 1996;7(2):143-52.
47. Isidor F. Mobility assessment with the Periotest system in relation to histologic findings of oral implants. Int J Oral Maxillofac Implants. 1998;13(3):377-83.
48. Jovanovic SA, Kenney EB, Carranza FA Jr, Donath K. The regenerative potential of plaque-induced peri-implant bone defects treated by a submerged membrane technique: an experimental study. Int J Oral Maxillofac Implants. 1993;8(1):13-8.
49. Klinge B. Implants in relation to natural teeth. J Clin Periodontol. 1991;18(6):482-7.
50. Koutouzis T, Eastman C, Chukkapalli S, Larjava H, Kesavalu L. A Novel Rat Model of Polymicrobial Peri-Implantitis: A Preliminary Study. J Periodontol. 2017;88(2):e32-e41.
51. Kozlovsky A, Tal H, Laufer BZ, Leshem R, Rohrer MD, Weinreb M, Artzi Z. Impact of implant overloading on the peri-implant bone in inflamed and non-inflamed peri-implant mucosa. Clin Oral Implants Res. 2007;18(5):601-10.
52. Lang NP, Brägger U, Walther D, Beamer B, Kornman KS. Ligature-induced peri-implant infection in cynomolgus monkeys. I. Clinical and radiographic findings. Clin Oral Implants Res. 1993 Mar;4(1):2-11. Erratum in: Clin Oral Implants Res 1993;4(2):111.
53. Lang NP, Wetzel AC, Stich H, Caffesse RG. Histologic probe penetration in healthy and inflamed peri-implant tissues. Clin Oral Implants Res. 1994;5(4):191-201.
54. Leonhardt A, Berglundh T, Ericsson I, Dahlén G. Putative periodontal pathogens on titanium implants and teeth in experimental gingivitis and periodontitis in beagle dogs. Clin Oral Implants Res. 1992;3(3):112-9.
55. Levin L, Zigdon H, Coelho PG, Suzuki M, Machtei EE. Reimplantation of dental implants following ligature-induced peri-implantitis: a pilot study in dogs. Clin Implant Dent Relat Res. 2013;15(1):1-6.
56. Lin X, Liu T, Wu G, Zheng Y, Wismeijer D, Liu Y. Peri-implantitis Induced by Stainless Steel Ligature in Beagle Dogs. Int J Periodontics Restorative Dent. 2017;37(3):e170-e179.
57. Lindhe J, Berglundh T, Ericsson I, Liljenberg B, Marinello C. Experimental breakdown of peri-implant and periodontal tissues. A study in the beagle dog. Clin Oral Implants Res. 1992;3(1):9-16.
58. López-Píriz R, Solá-Linares E, Granizo JJ, Díaz-Güemes I, Enciso S, Bartolomé JF, Cabal B, Esteban-Tejeda L, Torrecillas R, Moya JS. Radiologic evaluation of bone loss at implants with biocide coated titanium abutments: a study in the dog. PLoS One. 2012;7(12):e52861.
59. López-Píriz R, Solá-Linares E, Rodriguez-Portugal M, Malpica B, Díaz-Güemes I, Enciso S, Esteban-Tejeda L, Cabal B, Granizo JJ, Moya JS, Torrecillas R. Evaluation in a Dog Model of Three Antimicrobial Glassy Coatings: Prevention of Bone Loss around Implants and Microbial Assessments. PLoS One. 2015 Oct 21;10(10):e0140374.
60. Machado MA, Stefani CM, Sallum EA, Sallum AW, Tramontina VA, Nociti Júnior FH. Treatment of ligature-induced peri-implantitis defects by regenerative procedures: a clinical study in dogs. J Oral Sci. 1999;41(4):181-5.
61. Machado MA, Stefani CM, Sallum EA, Sallum AW, Tramontina VA, Nogueira-Filho GR, Nociti Júnior FH. Treatment of ligature-induced peri-implantitis defects by regenerative procedures. Part II: A histometric study in dogs. J Oral Sci. 2000;42(3):163-8.
62. Machtei EE, Kim DM, Karimbux N, Zigdon-Giladi H. The use of endothelial progenitor cells combined with barrier membrane for the reconstruction of peri-implant osseous defects: an animal experimental study. J Clin Periodontol. 2016;43(3):289-97.
63. Madi M, Zakaria O, Ichinose S, Kasugai S. Effect of Induced Periimplantitis on Dental Implants With and Without Ultrathin Hydroxyapatite Coating. Implant Dent. 2016;25(1):39-46.

64. Madi M, Zakaria O, Kasugai S. Coated vs uncoated implants: bone defect configurations after progressive peri-implantitis in dogs. J Oral Implantol. 2014;40(6):661-9.
65. Madi M, Zakaria O, Noritake K, Fuji M, Kasugai S. Peri-implantitis progression around thin sputtered hydroxyapatite-coated implants: clinical and radiographic evaluation in dogs. Int J Oral Maxillofac Implants. 2013;28(3):701-9.
66. Marinello CP, Berglundh T, Ericsson I, Klinge B, Glantz PO, Lindhe J. Resolution of ligature-induced peri-implantitis lesions in the dog. J Clin Periodontol. 1995;22(6):475-9.
67. Martines RT, Sendyk WR, Gromatzky A, Cury PR. Sandblasted/acid-etched vs smooth-surface implants: implant clinical reaction to experimentally induced peri-implantitis in Beagle dogs. J Oral Implantol. 2008;34(4):185-9.
68. Martinez A, Guitián F, López-Píriz R, Bartolomé JF, Cabal B, Esteban-Tejeda L, Torrecillas R, Moya JS. Bone loss at implant with titanium abutments coated by soda lime glass containing silver nanoparticles: a histological study in the dog. PLoS One. 2014 Jan 22;9(1):e86926.
69. Martins MC, Abi-Rached RS, Shibli JA, Araujo MW, Marcantonio E Jr. Experimental peri-implant tissue breakdown around different dental implant surfaces: clinical and radiographic evaluation in dogs. Int J Oral Maxillofac Implants. 2004;19(6):839-48.
70. Martins MC, Shibli JA, Abi-Rached RS, Marcantonio E Jr. Progression of experimental chronic peri-implantitis in dogs: clinical and radiographic evaluation. J Periodontol. 2005;76(8):1367-73.
71. McCracken M, Lemons JE, Jeffcoat M, Koth DL, Fritz ME. Histomorphological evaluation of loaded plate-form and root-form implants in Macaca mulatta monkeys. Clin Oral Implants Res. 2002;13(2):214-20.
72. Miyata T, Kobayashi Y, Araki H, Motomura Y, Shin K. The influence of controlled occlusal overload on peri-implant tissue: a histologic study in monkeys. Int J Oral Maxillofac Implants. 1998;13(5):677-83.
73. Miyata T, Kobayashi Y, Araki H, Ohto T, Shin K. The influence of controlled occlusal overload on peri-implant tissue. part 4: a histologic study in monkeys. Int J Oral Maxillofac Implants. 2002;17(3):384-90.
74. Miyata T, Kobayashi Y, Araki H, Ohto T, Shin K. The influence of controlled occlusal overload on peri-implant tissue. Part 3: A histologic study in monkeys. Int J Oral Maxillofac Implants. 2000;15(3):425-31.
75. Moest T, Wrede J, Schmitt CM, Stamp M, Neukam FW, Schlegel KA. The influence of different abutment materials on tissue regeneration after surgical treatment of peri-implantitis - a randomized controlled preclinical study. J Craniomaxillofac Surg. 2017;45(8):1190-1196.
76. Namgoong H, Kim MD, Ku Y, Rhyu IC, Lee YM, Seol YJ, Gu HJ, Susin C, Wikesjö UM, Koo KT. Bone reconstruction after surgical treatment of experimental peri-implantitis defects at a sandblasted/acid-etched hydroxyapatite-coated implant: an experimental study in the dog. J Clin Periodontol. 2015;42(10):960-6.
77. Nguyen Vo TN, Hao J, Chou J, Oshima M, Aoki K, Kuroda S, Kaboosaya B, Kasugai S. Ligature induced peri-implantitis: tissue destruction and inflammatory progression in a murine model. Clin Oral Implants Res. 2016 Jan 22. [Epub ahead of print]
78. Nociti FH Jr, Caffesse RG, Sallum EA, Machado MA, Stefani CM, Sallum AW. Evaluation of guided bone regeneration and/or bone grafts in the treatment of ligature-induced peri-implantitis defects: a morphometric study in dogs. J Oral Implantol. 2000;26(4):244-9.
79. Nociti FH Jr, Cesco De Toledo R, Machado MA, Stefani CM, Line SR, Gonçalves RB. Clinical and microbiological evaluation of ligature-induced peri-implantitis and periodontitis in dogs. Clin Oral Implants Res. 2001;12(4):295-300.
80. Nociti FH Jr, Machado MA, Stefani CM, Sallum EA, Sallum AW. Absorbable versus nonabsorbable membranes and bone grafts in the treatment of ligature-induced peri-implantitis defects in dogs. Part I. A clinical investigation. Clin Oral Implants Res. 2001;12(2):115-20.
81. Nociti FH Jr, Machado MA, Stefani CM, Sallum EA. Absorbable versus nonabsorbable membranes and bone grafts in the treatment of ligature-induced peri-implantitis defects in dogs: a histometric investigation. Int J Oral Maxillofac Implants. 2001;16(5):646-52.
82. Nociti Júnior FH, Caffesse RG, Sallum EA, Machado MA, Stefani CM, Sallum AW. Clinical study of guided bone regeneration and/or bone grafts in the treatment of ligature-induced peri-implantitis defects in dogs. Braz Dent J. 2001;12(2):127-31.
83. Park SY, Kim KH, Gwak EH, Rhee SH, Lee JC, Shin SY, Koo KT, Lee YM, Seol YJ. Ex vivo bone morphogenetic protein 2 gene delivery using periodontal ligament stem cells for enhanced re-osseointegration in the regenerative treatment of peri-implantitis. J Biomed Mater Res A. 2015;103(1):38-47.

84. Park SY, Kim KH, Rhee SH, Lee JC, Shin SY, Lee YM, Seol YJ. An immediate peri-implantitis induction model to study regenerative peri-implantitis treatments. Clin Oral Implants Res. 2015 May 9. doi: 10.1111/clr.12611. [Epub ahead of print]
85. Park SY, Kim KH, Shin SY, Koo KT, Lee YM, Chung CP, Seol YJ. Decontamination methods using a dental water jet and dental floss for microthreaded implant fixtures in regenerative periimplantitis treatment. Implant Dent. 2015;24(3):307-16.
86. Parlar A, Bosshardt DD, Cetiner D, Schafroth D, Unsal B, Haytaç C, Lang NP. Effects of decontamination and implant surface characteristics on re-osseointegration following treatment of peri-implantitis. Clin Oral Implants Res. 2009;20(4):391-9.
87. Pârvu AE, Tălu S, Taulescu MA, Bota A, Cătoi F, Crăciun C, Alb C, Pârvu O, Alb SF. Fractal analysis of ibuprofen effect on experimental dog peri-implantitis. Implant Dent. 2014;23(3):295-304.
88. Persson LG, Araújo MG, Berglundh T, Gröndahl K, Lindhe J. Resolution of peri-implantitis following treatment. An experimental study in the dog. Clin Oral Implants Res. 1999;10(3):195-203.
89. Persson LG, Berglundh T, Lindhe J, Sennerby L. Re-osseointegration after treatment of peri-implantitis at different implant surfaces. An experimental study in the dog. Clin Oral Implants Res. 2001;12(6):595-603.
90. Persson LG, Ericsson I, Berglundh T, Lindhe J. Guided bone regeneration in the treatment of periimplantitis. Clin Oral Implants Res. 1996;7(4):366-72.
91. Persson LG, Ericsson I, Berglundh T, Lindhe J. Osseintegration following treatment of peri-implantitis and replacement of implant components. An experimental study in the dog. J Clin Periodontol. 2001;28(3):258-63.
92. Persson LG, Mouhyi J, Berglundh T, Sennerby L, Lindhe J. Carbon dioxide laser and hydrogen peroxide conditioning in the treatment of periimplantitis: an experimental study in the dog. Clin Implant Dent Relat Res. 2004;6(4):230-8..
93. Pirih FQ, Hiyari S, Barroso AD, Jorge AC, Perussolo J, Atti E, Tetradis S, Camargo PM. Ligature-induced peri-implantitis in mice. J Periodontal Res. 2015;50(4):519-24.
94. Pirih FQ, Hiyari S, Leung HY, Barroso AD, Jorge AC, Perussolo J, Atti E, Lin YL, Tetradis S, Camargo PM. A Murine Model of Lipopolysaccharide-Induced Peri-Implant Mucositis and Peri-Implantitis. J Oral Implantol. 2015;41(5):e158-64.
95. Ramos UD, Suaid FA, Wikesjö UME, Susin C, Taba M Jr, Novaes AB Jr. Comparison between two antimicrobial protocols with or without guided bone regeneration in the treatment of peri-implantitis. A histomorphometric study in dogs. Clin Oral Implants Res. 2017;28(11):1388-1395.
96. Rodriguez JC, Koticha T, Eubanks DL, Rudek I, Molz FJ, Chiavaccini L, Claude A, Elder S, Wang HL. Influence of Microtextured Implant Surfaces on Peri-implantitis and Its Treatment: A Preclinical Trial. Int J Oral Maxillofac Implants. 2018;33(1):51–57.
97. Saito A, Hosaka Y, Sekiguchi K, Kigure T, Isobe S, Shibukawa Y, Sumii H, Ito T, Nakagawa T, Yamada S. Responses of peri-implant tissues to undisturbed plaque formation in dogs: clinical, radiographic, and microbiological findings. Bull Tokyo Dent Coll. 1997;38(1):13-20.
98. Schou S, Holmstrup P, Jørgensen T, Skovgaard LT, Stoltze K, Hjørting-Hansen E, Wenzel A. Anorganic porous bovine-derived bone mineral (Bio-Oss) and ePTFE membrane in the treatment of peri-implantitis in cynomolgus monkeys. Clin Oral Implants Res. 2003;14(5):535-47.
99. Schou S, Holmstrup P, Jørgensen T, Skovgaard LT, Stoltze K, Hjørting-Hansen E, Wenzel A. Implant surface preparation in the surgical treatment of experimental peri-implantitis with autogenous bone graft and ePTFE membrane in cynomolgus monkeys. Clin Oral Implants Res. 2003;14(4):412-22.
100. Schou S, Holmstrup P, Jørgensen T, Stoltze K, Hjørting-Hansen E, Wenzel A. Autogenous bone graft and ePTFE membrane in the treatment of peri-implantitis. I. Clinical and radiographic observations in cynomolgus monkeys. Clin Oral Implants Res. 2003;14(4):391-403.
101. Schou S, Holmstrup P, Keiding N, Fiehn NE. Microbiology of ligature-induced marginal inflammation around osseointegrated implants and ankylosed teeth in cynomolgus monkeys (Macaca fascicularis). Clin Oral Implants Res. 1996;7(3):190-200.
102. Schou S, Holmstrup P, Reibel J, Juhl M, Hjørting-Hansen E, Kornman KS. Ligature-induced marginal inflammation around osseointegrated implants and ankylosed teeth: stereologic and histologic observations in cynomolgus monkeys (Macaca fascicularis). J Periodontol. 1993;64(6):529-37.

103. Schou S, Holmstrup P, Skovgaard LT, Stoltze K, Hjørting-Hansen E, Gundersen HJ. Autogenous bone graft and ePTFE membrane in the treatment of peri-implantitis. II. Stereologic and histologic observations in cynomolgus monkeys. Clin Oral Implants Res. 2003;14(4):404-11.
104. Schou S, Holmstrup P, Stoltze K, Hjørting-Hansen E, Fiehn NE, Skovgaard LT. Probing around implants and teeth with healthy or inflamed peri-implant mucosa/gingiva. A histologic comparison in cynomolgus monkeys (Macaca fascicularis). Clin Oral Implants Res. 2002;13(2):113-26.
105. Schou S, Holmstrup P, Stoltze K, Hjørting-Hansen E, Kornman KS. Ligature-induced marginal inflammation around osseointegrated implants and ankylosed teeth. Clin Oral Implants Res. 1993;4(1):12-22.
106. Schüpbach P, Hürzeler M, Grunder U. Implant-tissue interfaces following treatment of peri-implantitis using guided tissue regeneration: a light and electron microscopic study. Clin Oral Implants Res. 1994;5(2):55-65.
107. Schwarz F, Herten M, Sager M, Bieling K, Sculean A, Becker J. Comparison of naturally occurring and ligature-induced peri-implantitis bone defects in humans and dogs. Clin Oral Implants Res. 2007 Apr;18(2):161-70. Erratum in: Clin Oral Implants Res. 2007;18(3):397.
108. Schwarz F, Jepsen S, Herten M, Sager M, Rothamel D, Becker J. Influence of different treatment approaches on non-submerged and submerged healing of ligature induced peri-implantitis lesions: an experimental study in dogs. J Clin Periodontol. 2006;33(8):584-95.
109. Schwarz F, Sahm N, Mihatovic I, Golubovic V, Becker J. Surgical therapy of advanced ligature-induced peri-implantitis defects: cone-beam computed tomographic and histological analysis. J Clin Periodontol. 2011;38(10):939-49.
110. Sennerby L, Persson LG, Berglundh T, Wennerberg A, Lindhe J. Implant stability during initiation and resolution of experimental periimplantitis: an experimental study in the dog. Clin Implant Dent Relat Res. 2005;7(3):136-40.
111. Shi Q, Song K, Zhou X, Xiong Z, Du T, Lu X, Cao Y. Effects of non-equilibrium plasma in the treatment of ligature-induced peri-implantitis. J Clin Periodontol. 2015;42(5):478-87.
112. Shibli JA, Martins MC, Lotufo RF, Marcantonio E Jr. Microbiologic and radiographic analysis of ligature-induced peri-implantitis with different dental implant surfaces. Int J Oral Maxillofac Implants. 2003;18(3):383-90.
113. Shibli JA, Martins MC, Nociti FH Jr, Garcia VG, Marcantonio E Jr. Treatment of ligature-induced peri-implantitis by lethal photosensitization and guided bone regeneration: a preliminary histologic study in dogs. J Periodontol. 2003;74(3):338-45.
114. Shibli JA, Martins MC, Ribeiro FS, Garcia VG, Nociti FH Jr, Marcantonio E Jr. Lethal photosensitization and guided bone regeneration in treatment of peri-implantitis: an experimental study in dogs. Clin Oral Implants Res. 2006;17(3):273-81.
115. Shibli JA, Martins MC, Theodoro LH, Lotufo RF, Garcia VG, Marcantonio EJ. Lethal photosensitization in microbiological treatment of ligature-induced peri-implantitis: a preliminary study in dogs. J Oral Sci. 2003;45(1):17-23.
116. Shibutani T, Inuduka A, Horiki I, Luan Q, Iwayama Y. Bisphosphonate inhibits alveolar bone resorption in experimentally-induced peri-implantitis in dogs. Clin Oral Implants Res. 2001 Apr;12(2):109-14.
117. Singh G, O'Neal RB, Brennan WA, Strong SL, Horner JA, Van Dyke TE. Surgical treatment of induced peri-implantitis in the micro pig: clinical and histological analysis. J Periodontol. 1993;64(10):984-9.
118. Stübinger S, Henke J, Donath K, Deppe H. Bone regeneration after peri-implant care with the CO2 laser: a fluorescence microscopy study. Int J Oral Maxillofac Implants. 2005;20(2):203-10.
119. Takamori Y, Atsuta I, Nakamura H, Sawase T, Koyano K, Hara Y. Histopathological comparison of the onset of peri-implantitis and periodontitis in rats. Clin Oral Implants Res. 2016 Jan 25. doi: 10.1111/clr.12777. [Epub ahead of print]
120. Takasaki AA, Aoki A, Mizutani K, Kikuchi S, Oda S, Ishikawa I. Er:YAG laser therapy for peri-implant infection: a histological study. Lasers Med Sci. 2007;22(3):143-57.
121. Tillmanns HW, Hermann JS, Cagna DR, Burgess AV, Meffert RM. Evaluation of three different dental implants in ligature-induced peri-implantitis in the beagle dog. Part I. Clinical evaluation. Int J Oral Maxillofac Implants. 1997;12(5):611-20.
122. Tillmanns HW, Hermann JS, Tiffee JC, Burgess AV, Meffert RM. Evaluation of three different dental implants in ligature-induced peri-implantitis in the beagle dog. Part II. Histology and microbiology. Int J Oral Maxillofac Implants. 1998;13(1):59-68.

123. Trejo PM, Bonaventura G, Weng D, Caffesse RG, Bragger U, Lang NP. Effect of mechanical and antiseptic therapy on peri-implant mucositis: an experimental study in monkeys. Clin Oral Implants Res. 2006;17(3):294-304.
124. Warrer K, Buser D, Lang NP, Karring T. Plaque-induced peri-implantitis in the presence or absence of keratinized mucosa. An experimental study in monkeys. Clin Oral Implants Res. 1995;6(3):131-8.
125. Watzak G, Zechner W, Tangl S, Vasak C, Donath K, Watzek G. Soft tissue around three different implant types after 1.5 years of functional loading without oral hygiene: a preliminary study in baboons. Clin Oral Implants Res. 2006;17(2):229-36.
126. Weber HP, Fiorellini JP, Paquette DW, Howell TH, Williams RC. Inhibition of peri-implant bone loss with the nonsteroidal anti-inflammatory drug flurbiprofen in beagle dogs. A preliminary study. Clin Oral Implants Res. 1994;5(3):148-53.
127. Wetzel AC, Vlassis J, Caffesse RG, Hämmerle CH, Lang NP. Attempts to obtain re-osseointegration following experimental peri-implantitis in dogs. Clin Oral Implants Res. 1999;10(2):111-9.
128. Wong RL, Hiyari S, Yaghsezian A, Davar M, Lin YL, Galvan M, Tetradis S, Camargo PM, Pirih FQ. Comparing the Healing Potential of Late-Stage Periodontitis and Peri-Implantitis. J Oral Implantol. 2017;43(6):437-445.
129. Xu L, Sun X, Bai J, Jiang L, Wang S, Zhao J, Xia L, Zhang X, Wen J, Li G, Jiang X. Reosseointegration Following Regenerative Therapy of Tissue-Engineered Bone in a Canine Model of Experimental Peri-Implantitis. Clin Implant Dent Relat Res. 2016;18(2):379-91.
130. You TM, Choi BH, Zhu SJ, Jung JH, Lee SH, Huh JY, Lee HJ, Li J. Treatment of experimental peri-implantitis using autogenous bone grafts and platelet-enriche fibrin glue in dogs. Oral Surg Oral Med Oral Pathol Oral Radiol Endod. 2007;103(1):34-7.
131. Yu X, Hu Y, Freire M, Yu P, Kawai T, Han X. Role of toll-like receptor 2 in inflammation and alveolar bone loss in experimental peri-implantitis versus periodontitis. J Periodontal Res. 2018;53(1):98-106.
132. Zechner W, Kneissel M, Kim S, Ulm C, Watzek G, Plenk H Jr. Histomorphometrical and clinical comparison of submerged and nonsubmerged implants subjected to experimental peri-implantitis in dogs. Clin Oral Implants Res. 2004;15(1):23-33.
133. Zitzmann NU, Berglundh T, Ericsson I, Lindhe J. Spontaneous progression of experimentally induced periimplantitis. J Clin Periodontol. 2004;31(10):845-9.

b. Publications excluded from the review

1. Alam J, Baek KJ, Choi YS, Kim YC, Choi Y. N-acetylcysteine and the human serum components that inhibit bacterial invasion of gingival epithelial cells prevent experimental periodontitis in mice. J Periodontal Implant Sci. 2014 Dec;44(6):266-73.
2. Bhattarai G, Poudel SB, Kook SH, Lee JC. Resveratrol prevents alveolar bone loss in an experimental rat model of periodontitis. Acta Biomater. 2016 Jan;29:398-408.
3. Chen H, Xu X, Liu M, Zhang W, Ke HZ, Qin A, Tang T, Lu E. Sclerostin antibody treatment causes greater alveolar crest height and bone mass in an ovariectomized rat model of localized periodontitis. Bone. 2015 Jul;76:141-8.
4. Do MJ, Kim K, Lee H, Cha S, Seo T, Park HJ, Lee JS, Kim TI. Development of animal experimental periodontitis models. J Periodontal Implant Sci. 2013 Aug;43(4):147-52.
5. Hanisch O, Tatakis DN, Boskovic MM, Rohrer MD, Wikesjö UM. Bone formation and reosseointegration in peri-implantitis defects following surgical implantation of rhBMP-2. Int J Oral Maxillofac Implants. 1997 Sep-Oct;12(5):604-10.
6. Klinge B, Kuvatanasuhati J, Attström R, Kalfas S, Edwardsson S. The effect of topical metronidazole therapy on experimentally-induced periodontitis in the beagle dog. J Clin Periodontol. 1992 Oct;19(9 Pt 2):702-7.
7. Lee D, Sohn B, Kim KH, Kim S, Koo KT, Kim TI, Seol YJ, Lee YM, Rhyu IC, Ku Y. Effects of Untreated Periodontitis on Osseointegration of Dental Implants in a Beagle Dog Model. J Periodontol. 2016 Oct;87(10):1141-8.
8. Misch CE, Suzuki JB, Misch-Dietsh FM, Bidez MW. A positive correlation between occlusal trauma and peri-implant bone loss: literature support. Implant Dent. 2005 Jun;14(2):108-16.
9. Papageorgiou A, Vouros I, Konstantinidis A. Treatment outcomes of ligature-induced recession in the dog model using guided tissue regeneration or coronally positioned flap procedures. J Int Acad Periodontol. 2009 Apr;11(2):177-87.

10. Pimentel SP, Sallum AW, Saldanha JB, Casati MZ, Nociti FH Jr, Sallum EA. Enamel matrix derivative versus guided tissue regeneration in the presence of nicotine: a histomorphometric study in dogs. J Clin Periodontol. 2006 Dec;33(12):900-7.
11. Pongnarisorn NJ, Gemmell E, Tan AE, Henry PJ, Marshall RI, Seymour GJ. Inflammation associated with implants with different surface types. Clin Oral Implants Res. 2007 Feb;18(1):114-25.
12. Schwarz F, Mihatovic I, Golubovic V, Bradu S, Sager M, Becker J. Impact of plaque accumulation on the osseointegration of titanium-zirconium alloy and titanium implants. A histological and immunohistochemical analysis. Clin Oral Implants Res. 2015 Nov;26(11):1281-7.
13. Sousa V, Mardas N, Spratt D, Boniface D, Dard M, Donos N. Experimental models for contamination of titanium surfaces and disinfection protocols. Clin Oral Implants Res. 2016 Oct;27(10):1233-1242.
14. Xu XC, Chen H, Zhang X, Zhai ZJ, Liu XQ, Qin A, Lu EY. Simvastatin prevents alveolar bone loss in an experimental rat model of periodontitis after ovariectomy. J Transl Med. 2014 Oct 1;12:284.
15. Zitzmann NU, Abrahamsson I, Berglundh T, Lindhe J. Soft tissue reactions to plaque formation at implant abutments with different surface topography. An experimental study in dogs. J Clin Periodontol. 2002 May;29(5):456-61.

Reference s2: list of 35 studies included in the quantitative synthesis

1. Lindhe J, Berglundh T, Ericsson I, Liljenberg B, Marinello C. Experimental breakdown of peri-implant and periodontal tissues. A study in the beagle dog. Clin Oral Implants Res. 1992;3(1):9-16.
2. Lang NP, Brägger U, Walther D, Beamer B, Kornman KS. Ligature-induced peri-implant infection in cynomolgus monkeys. I. Clinical and radiographic findings. Clin Oral Implants Res. 1993 Mar;4(1):2-11. Erratum in: Clin Oral Implants Res 1993;4(2):111.
3. Cook SD, Rust-Dawicki AM. In vivo evaluation of CSTi dental implants in the presence of ligature-induced peri-implantitis. J Oral Implantol. 1995;21(3):191-200.
4. Persson LG, Ericsson I, Berglundh T, Lindhe J. Guided bone regeneration in the treatment of periimplantitis. Clin Oral Implants Res. 1996;7(4):366-72.
5. Fritz ME, Braswell LD, Koth D, Jeffcoat M, Reddy M, Cotsonis G. Experimental peri-implantitis in consecutively placed, loaded root-form and plate-form implants in adult Macaca mulatta monkeys. J Periodontol. 1997;68(11):1131-5.
6. Hanisch O, Cortella CA, Boskovic MM, James RA, Slots J, Wikesjö UM. Experimental peri-implant tissue breakdown around hydroxyapatite-coated implants. J Periodontol. 1997;68(1):59-66.
7. Abrahamsson I, Berglundh T, Lindhe J. Soft tissue response to plaque formation at different implant systems. A comparative study in the dog. Clin Oral Implants Res. 1998;9(2):73-9.
8. Hürzeler MB, Quiñones CR, Kohal RJ, Rohde M, Strub JR, Teuscher U, Caffesse RG. Changes in peri-implant tissues subjected to orthodontic forces and ligature breakdown in monkeys. J Periodontol. 1998;69(3):396-404.
9. Tillmanns HW, Hermann JS, Tiffee JC, Burgess AV, Meffert RM. Evaluation of three different dental implants in ligature-induced peri-implantitis in the beagle dog. Part II. Histology and microbiology. Int J Oral Maxillofac Implants. 1998;13(1):59-68.
10. Persson LG, Araújo MG, Berglundh T, Gröndahl K, Lindhe J. Resolution of peri-implantitis following treatment. An experimental study in the dog. Clin Oral Implants Res. 1999;10(3):195-203.
11. Deppe H, Horch HH, Henke J, Donath K. Peri-implant care of ailing implants with the carbon dioxide laser. Int J Oral Maxillofac Implants. 2001;16(5):659-67.
12. Persson LG, Berglundh T, Lindhe J, Sennerby L. Re-osseointegration after treatment of peri-implantitis at different implant surfaces. An experimental study in the dog. Clin Oral Implants Res. 2001;12(6):595-603.
13. Shibutani T, Inuduka A, Horiki I, Luan Q, Iwayama Y. Bisphosphonate inhibits alveolar bone resorption in experimentally-induced peri-implantitis in dogs. Clin Oral Implants Res. 2001 Apr;12(2):109-14.
14. Shibli JA, Martins MC, Lotufo RF, Marcantonio E Jr. Microbiologic and radiographic analysis of ligature-induced peri-implantitis with different dental implant surfaces. Int J Oral Maxillofac Implants. 2003;18(3):383-90.
15. Deppe H, Wagenpfeil S, Donath K. Comparative value of attachment measurements in implant dentistry. Int J Oral Maxillofac Implants. 2004;19(2):208-15.
16. Martins MC, Abi-Rached RS, Shibli JA, Araujo MW, Marcantonio E Jr. Experimental peri-implant tissue breakdown around different dental implant surfaces: clinical and radiographic evaluation in dogs. Int J Oral Maxillofac Implants. 2004;19(6):839-48.
17. Martins MC, Shibli JA, Abi-Rached RS, Marcantonio E Jr. Progression of experimental chronic peri-implantitis in dogs: clinical and radiographic evaluation. J Periodontol. 2005;76(8):1367-73.
18. Berglundh T, Gotfredsen K, Zitzmann NU, Lang NP, Lindhe J. Spontaneous progression of ligature induced peri-implantitis at implants with different surface roughness: an experimental study in dogs. Clin Oral Implants Res. 2007;18(5):655-61.
19. Shibli JA, Martins MC, Ribeiro FS, Garcia VG, Nociti FH Jr, Marcantonio E Jr. Lethal photosensitization and guided bone regeneration in treatment of peri-implantitis: an experimental study in dogs. Clin Oral Implants Res. 2006;17(3):273-81.
20. Albouy JP, Abrahamsson I, Persson LG, Berglundh T. Spontaneous progression of peri-implantitis at different types of implants. An experimental study in dogs. I: clinical and radiographic observations. Clin Oral Implants Res. 2008;19(10):997-1002.
21. Albouy JP, Abrahamsson I, Berglundh T. Spontaneous progression of experimental peri-implantitis at implants with different surface characteristics: an experimental study in dogs. J Clin Periodontol. 2012;39(2):182-7.

22. López-Píriz R, Solá-Linares E, Granizo JJ, Díaz-Güemes I, Enciso S, Bartolomé JF, Cabal B, Esteban-Tejeda L, Torrecillas R, Moya JS. Radiologic evaluation of bone loss at implants with biocide coated titanium abutments: a study in the dog. PLoS One. 2012;7(12):e52861.
23. Carcuac O, Abrahamsson I, Albouy JP, Linder E, Larsson L, Berglundh T. Experimental periodontitis and peri-implantitis in dogs. Clin Oral Implants Res. 2013;24(4):363-71.
24. Madi M, Zakaria O, Noritake K, Fuji M, Kasugai S. Peri-implantitis progression around thin sputtered hydroxyapatite-coated implants: clinical and radiographic evaluation in dogs. Int J Oral Maxillofac Implants. 2013;28(3):701-9.
25. Martinez A, Guitián F, López-Píriz R, Bartolomé JF, Cabal B, Esteban-Tejeda L, Torrecillas R, Moya JS. Bone loss at implant with titanium abutments coated by soda lime glass containing silver nanoparticles: a histological study in the dog. PLoS One. 2014 Jan 22;9(1):e86926.
26. Carcuac O, Abrahamsson I, Charalampakis G, Berglundh T. The effect of the local use of chlorhexidine in surgical treatment of experimental peri-implantitis in dogs. J Clin Periodontol. 2015;42(2):196-203.
27. Huang B, Piao M, Zhang L, Wang X, Xu L, Zhu W, Meng H. Ligature-induced peri-implant infection in crestal and subcrestal implants: a clinical and radiographic study in dogs. PeerJ. 2015 Jul 30;3:e1139.
28. Namgoong H, Kim MD, Ku Y, Rhyu IC, Lee YM, Seol YJ, Gu HJ, Susin C, Wikesjö UM, Koo KT. Bone reconstruction after surgical treatment of experimental peri-implantitis defects at a sandblasted/acid-etched hydroxyapatite-coated implant: an experimental study in the dog. J Clin Periodontol. 2015;42(10):960-6.
29. López-Píriz R, Solá-Linares E, Rodriguez-Portugal M, Malpica B, Díaz-Güemes I, Enciso S, Esteban-Tejeda L, Cabal B, Granizo JJ, Moya JS, Torrecillas R. Evaluation in a Dog Model of Three Antimicrobial Glassy Coatings: Prevention of Bone Loss around Implants and Microbial Assessments. PLoS One. 2015 Oct 21;10(10):e0140374.
30. Pirih FQ, Hiyari S, Barroso AD, Jorge AC, Perussolo J, Atti E, Tetradis S, Camargo PM. Ligature-induced peri-implantitis in mice. J Periodontal Res. 2015;50(4):519-24.
31. Ishii K, Matsuo M, Hoshi N, Takahashi SS, Kawamata R, Kimoto K. Effect of Ultraviolet Irradiation of the Implant Surface on Progression of Periimplantitis-A Pilot Study in Dogs. Implant Dent. 2016;25(1):47-53.
32. Godoy-Gallardo M, Manzanares-Céspedes MC, Sevilla P, Nart J, Manzanares N, Manero JM, Gil FJ, Boyd SK, Rodríguez D. Evaluation of bone loss in antibacterial coated dental implants: An experimental study in dogs. Mater Sci Eng C Mater Biol Appl. 2016;69(1):538-45.
33. Park SY, Kim KH, Rhee SH, Lee JC, Shin SY, Lee YM, Seol YJ. An immediate peri-implantitis induction model to study regenerative peri-implantitis treatments. Clin Oral Implants Res. 2017;28(1):36-42.
34. Lin X, Liu T, Wu G, Zheng Y, Wismeijer D, Liu Y. Peri-implantitis Induced by Stainless Steel Ligature in Beagle Dogs. Int J Periodontics Restorative Dent. 2017;37(3):e170-e179.
35. Koutouzis T, Eastman C, Chukkapalli S, Larjava H, Kesavalu L. A Novel Rat Model of Polymicrobial Peri-Implantitis: A Preliminary Study. J Periodontol. 2017;88(2):e32-e41.
