# Supplementary material for: Ligature-Induced Experimental Peri-Implantitis—A Systematic Review
Source: J Clin Med. 2018 Nov 28;7(12):492. doi: 10.3390/jcm7120492 (PMC6306937; doi:10.3390/jcm7120492)
Supplement: Supplementary file 1 [file jcm-07-00492-s001.zip › Tables S1 S2 S3 and S4.pdf]

Table S1. Main characteristics of the studies included in the qualitative synthesis.

Author	Published	Species	Comparison (X vsY)	No. of animals	Jaw	No. of extracted teeth	Healing period (m=months, w=weeks)	Study design (split mouth, inter quadrant)	Type of implant					Surgical protocol					Pre- and postoperative care				
									Shape (cylindrical/ screw-type)	Size (witdh/ Length) (mm)	Material	Coating	Bone level/ Tissue level	Nr of implants/ animal	1 stage/ 2 stage surgery	Implant center to center distance (mm)	Time implant healing + abutment-PI induction	Randomisation	Loading	Antibiotic	Plaque control timing (before/ after induction)	Plaque control duration (months)	Plaque control method
1. Klinge	1991	Dog, Beagle	Implants vs teeth – Pilot studys	2	Mandible	All PMs on the right side	75d	Split mouth – implants vs teeth	Screw-type (Nobelpharma Company)	NA	Ti	NA (U?)	Bone level	7: 3 in one dog and 4 in the other	2-stage	NA	6m+4w	No	NA-no suprastructure	Na – perioperative drugs no specified	NA	NA	NA
2. Hickey et al	1991	Microswine	Microbiologic changes	2	Mandible	All mandibular PMs	>4m	Split mouth	Screw shaped	NA Bånemark	Ti	U	Bone level	6: 3 in each side of the mandible	2-stage	NA	2m+2w	Yes – Ligatures on randomised side in the first swine. Other side used in the second swine	NA (no suprastructure)	NA	(A) Only control side. 2w after abutment placement (day 0)	(A) 45d	(A) Scaled and cleaned free of plaque and calculus every 5d
3. Akagawa et al.	1992	Monkey, Macaca fuscata	Ligature vs no ligature on implants and teeth	9	Maxilla and mandible	6 (Maxillary lateral incisor, mandibular second premolar and first molar on both sides.)	3m	Split mouth with ligatures on one side and plaque control on the other. Also inter-quadrant comparison between teeth and implants.	Screw-type with integrated abutment for cementum retained restoration	NA	Sapphire	NA	Tissue level	4 (1 in each quadrant)	1 stage	NA	3m + 0	No	NA Implants were connected to both neighboring teeth with a fixed suprastructure 1 month after implant insertion	0,5 g/day cefaloligine intramuscularly for three days postoperatively	(1) 2 months after extraction (2) After implant installation (3) 2 weeks after implant installation	(1) 1m (2) 2w (3) 3m minus 2w	(1) Brush, interdental brush, electric pencil brush, CHX 0,2%. Interval NA. (2) CHX twice a week (3) same as (1)
4. Ericsson et al	1992	Dog, Beagle	Effects of plaque accumulation around implants vs teeth	5	Mandible, right side	Right premolars	3m	Split mouth	Screw shaped (Brånemark)	NA	Ti	NA	Bone level	3 on the right side	2-stage	NA	3m+4m	No	Na (No suprastructure used)	NA	After abutment connection	4m	Brush and dentifrice daily
5. Leonhardt et al	1992	Dog, Beagle	Bacteria around implants vs teeth	4	Mandible	PM1 PM2 PM3 PM4 On the right side	3m	Split mouth, implants on left side, teeth on the right	Screw shaped Brånemark	NA	Ti	Turned	Bone level	2 implants and 2 teeth. Third implant used in Lindhe 1992	2-stage	NA	3m+2m (at which plaque accumulation was initiated. Ligatures placed 49d later)	No	NA (No suprastructure)	NA	(A) After abutment connection	(A) 2m	(A) Brush and dentifrice daily
6. Lindhe et al	1992	Dog, Beagle		5	Mandible	PM1 PM2 PM3 PM4	3m	Split mouth, implants on left side, teeth on the right	Screw shaped Brånemark	NA	Ti	Turned	Bone level	3 on the right side of the mandible	2-stage	NA	3m+6m	No	NA (No suprastructure)	NA	(A) After abutment connection	(A) 6m	(A) Brush and dentifrice daily

						On the right side																	
7. Akagawa et al	1993	Monkey, Macaca fuscata	Ligature vs no ligature on implants and teeth	9	Maxilla and mandible	6 (Maxillary lateral incisor, mandibular second premolar and first molar on both sides.)	3m	Split mouth with ligatures on one side and plaque control on the other. Also inter-quadrant comparison between teeth and implants inter-quadrant.	Screw-type with integrated abutment for cementum retained restoration	NA	Sapphire	NA	Tissue level	4 (1 in each quadrant)	1 stage	NA	3m + 0	No	NA Implants were connected to both neighboring teeth with a fixed suprastructure 1 month after implant insertion	0,5 g/day cefaloligine intramuscularly for three days postoperatively	(1) 2 months after extraction (2) After implant installation (3) 2 weeks after implant installation	(1) 1m (2) 2w (3) 3m minus 2w	(1) Brush, interdental brush, electric pencil brush, CHX 0,2%. Interval NA. (2) CHX twice a week (3) same as (1)
8. Grunder et al	1993	Dog, Beagle	TREATMENT	10	Mandible, both sides	4: 2 in each side of the mandible (PM3, PM4)	6m	Split-mouth	Screw-shape (Screw-vent, Dentslply)	3,25x7	Ti	NA	Bone level	4: 2 on each side	2-stage	NA	2m+2m	Yes treatment: toss of a coin	NA (Ti-abutments, loading not mentioned)	NA	(A) after implantation & after abutment & after peri-implant surgery (B) 1w after abutment connection (D) 1w after peri-implant surgery	(A) 1w (B) 2m (C) 3m	(A) CHX daily (B) Brush, water, pumice daily (C) same as (B)
9. Jovanovic et al	1993	Dog, Beagle	TREATMENT STUDY new bone formation on 3 different surfaces	3	Mandible	M1, all PMs	12w	5 implants with mixed surfaces on each side of the mandible	Screw shaped	(A) Brånemark 4x10 mm (B) IMZ 4x8 mm (C) Integral4x 8 mm	Ti	(A) U (B) TPS (C) HA-implant	Bone level	10: 5 in each side of the mandible	1-stage	NA	0+0m (ligature placed at same time as implants)	No	No	NA	NA	NA	NA
10. Lang et al	1993	Monkey, cynomolgus	Plaque accumulation vs ligature	4	Mandible	6: PM2 M1 M2 On bot sides	90d	Interquadrant: One implant with ligature and one without in each quadrant	Cylindrical ITI type F	6,0x3,0 mm)	Ti	TPS	Tissue level	4: 2 on each side of the mandible	1-stage	NA	60d+0d	NA	No	NA	(A) After implant placement (B) From day 60-	(A) 60d (B) 30d	(A) Rubber cup and pumice 3 times/ w. (B) Suspended plaque control
11. Schou et al	1993	Monkey, Macaca Fascicularis	Marginal inflammation round implants vs ankylosed teeth – histologic study of ICT	8	Mandible	M1, M2 on both sides	12w	Split mouth, liagtures on one side (ligature vs no ligature). Upper jaw vs lower jaw	Cylindrical, custom made. Collar on ligature implants for ligature retention	2,5 x 10 mm	Ti-coated polycarbonate	Ti coated polycarbonate	Tissuelevel	4: 2 on each side	1 stage	-	12w + 0	YES	No	Streptomycin - benzylpenicillin (Streptocillin Vet., 200.000 IE/ml, 1 ml),	(1) 2w before and 2w after extractions (2) 2w before and 8w after implant surgery	(1) 4w (2) 10w	(1) Brush, 2% CHX solution 3 min, 3t/ w. (2) same as 1

								(implant, vs tooth)												2h preop. And once a day for 10 d. post op			
12. Schou et al	1993	Monkey, Macaca Fascicularis	Marginal inflammation round implants vs ankylosed teeth	8	Mandible	M1, M2 on both sides	12w	Split mouth, liagtures on one side (ligature vs no ligature). Upper jaw vs lower jaw (implant, vs tooth)	Cylindrical, custom made. Collar on ligature implants for ligature retention	2,5 x 10 mm	Ti-coated polycarbonate	Ti coated polycarbonate	Tissuelevel	4: 2 on each side	1 stage	-	12w + 0	YES	No	Streptomycin - benzylpenicillin (Streptocillin Vet., 200.000 IE/ml, 1 ml), 2h preop. And once a day for 10 d. post op	(1) 2w before and 2w after extractions (2) 2w before and 8w after implant surgery	(1) 4w (2) 10w	(1) Brush, 2% CHX solution 3 min, 3t/ w. (2) same as 1
13. Singh et al	1993	Micro Pig	TREATMENT STUDY	1 (originally 2, but 1 lost)	Mandible	NA	NA	Inter-quadrant	Root-form implant (Nobelpharma, USA, Chicago, IL).	NA	Ti	NA	NA	6: 3 on each side	NA	NA	NA	NA	NA	NA	NA	NA	NA
14. Lang et al	1994	Dog, Beagle		5	Mandible	6: PM2 PM3 PM4 On both sides	4m		Cylindrical ITI	2,8x6 mm	Ti	TPS	Tissue level	6: 3 on each side of the mandible	1-stage	4 mm adjacent to neighbouring teeth and 4 mm between the implants	0+2m	No	No	Penicillin for 5d after implant insertion	(A) After tooth extractions (B) 1w after implant insertion	(A) 4m (B) 2m	(A) Rubber cup and pumice once a week. Soft brush 3 times/ w. (B) same as (A) + CHX 0,12%.
15. Schüpbach et al	1994	Dog, Beagle	TREATMENT – Implant-tissue interfaces following PI-treatment with GBR	10	Mandible, both sides	4: 2 in each side of the mandible (PM3, PM4)	6m	Split-mouth	Screw-shape (Screw-vent, Dentslply)	3,25x7	Ti	NA	Bone level	4: 2 on each side	2-stage	NA	2m+2m	Yes treatment: toss of a coin	NA (Ti-abutments, loading not mentioned)	NA	(A) after implantation & after abutment & after peri-implant surgery (B) 1w after abutment connection (D) 1w after peri-implant surgery	(A) 1w (B) 2m (C) 3m	(A) CHX daily (B) Brush, water, pumise daily (C) same as (B)
16. Weber et al	1994	Dog, beagle	NSAID: Inhibition of peri-implant bone loss	2	Mandible	All PMs and M1 on both sides	3m	Comparison between animals	Cylindrical, tissue level (Straumann AG, Walderinburg, Switzerland)	2,05 x 8 mm	(1) Ti	TPS	Tissue level	10: 5 on each side	1-stage	NA	3m+o	No	No loading	Bicillin 600,000 units IM prior to implant insertion	(a) after surgery (b) 14 days after implant insertion	(a) 2w (b) 2,5m	(a) 0,12% CHX rinse daily (b) Brush + 2% CHX gel daily
17. Cook et al	1995	Dog, Mongrel	2 implant surfaces	14	Mandible, implants on both sides	4 premolars on each	2m	Interquadrant	Cylindrical (Calicitec implants)	4x10	Ti	A: Cancellous-structured titanium B: HA-coated	Bone level	6 (3 on each side. 2 implants were not	2-stage	NA	8w+0	NA	NA (although no suprastruct	5 days postoperatively	None at the test side. At the time of abutment	From abutment surgery to end of study	Weekly brushing

						side of the mandible								inserted due to anatomical reasons)					ure was used)		connection on the control side.		
18. Ericsson	1995	Dog, Labrador	Plaque control vs no plaque control	5	Mandible, both sides	8: 4 each side (1stM, 4thPM, 3rdPM, 2ndPM)	3m	Interquadrant	Screw shaped (Brånemark)	3,75x10	Ti	NA	Bone level	6: 3 on each side	2-stage	NA	3m+3m	No	Na (No suprastructure)	NA	After abutment connection	3m	Brush and dentifrice daily
19. Hürzeler et al	1995	Dog, Beagle	TREATMENT STUDY 6 different treatments	4	Mandible	All mandibular PMs and M1	3m	Ligatures around all implants	Screw Shaped	3,75x7 mm Brånemark	Ti	U	Bone level	6: 3 on each side of the mandible	2-stage	NA	3m+2w	Yes, according to treatment	No.	Yes, after ligature removal. systemic Metronidazole hydrochloride 250mg daily for 3w	(A) After ligature removal (B) After Peri-implantitis surgery	(A) 2w (B) 3w	(A) Daily brushing with flour of pumice mixed with 0,12% CHX + 0,12% CHX spray. (B) 0,12% CHX spray
20. Marinello et al	1995	Dog, Labrador	Bone loss at different time intervals from ligature removal	5	Mandible	PM3 PM4 M1 On both sides of the mandible	3m	Comparison between dogs at different time points from ligature removal	Screw shaped, Brånemark	3,75x10 mm	Ti	NA	Bone level	4: 2 on each side of the mandible	2-stage	NA	3m+6m	No	No	NA	(A) after abutment surgery	(A) 6m	(A) tooth and implant cleaning 1t/d
21. Warrer et al	1995	Monkey, Macaca Fascicularis	Bone level and clinical features with ligature or passive plaque accumulation in presence or absence of keratinized mucosa	5	Mandible	PM2, M1, M2 on both sides	3m	Split mouth	Cylindrical (ITI, Hollow cylinder)	3x 8 mm	Ti	TPS	Tissue level	6: 3 on each side 22 implants included in study, remaining implants reported elsewhere	1-stage	Equal distance between implants and adjacent teeth	3m and 10d + 0	Yes, 8 implants placed in keratinized mucosa and 8 placed in non-keratinized mucosta randomly chosen	No – all placed in infraocclusion	Penicillin, Procaine Vet. Rosco, 0,13 ml/kg the first day post op	(A) 10d after implant surgery	(A) 3m	(A) CHX 0,2% applied with soft bristle brusch 1t/w.
22. Ericsson et al	1996	Dog, Labrador	TREATMENT STUDY systemic antibiotics with/without local debridement	5	Mandible, both sides	6: 3 each side (1st M, 4th PM, 3rd PM)	3m	Split mouth	Screw shaped (Brånemark)	3,75x10	Ti	NA	Bone level	6: 3 on each side	2-stage	NA	3m+3m	No	NA (No suprastructure)	NA	Immediately following abutment connection	3m	Tooth and implant cleaning 3times/w
23. Isidor	1996	Monkey, Macaca Fascicularis	Overload vs ligature	4	Mandible	M1, all PMs, all Is	8m	Split mouth	Screw shaped	3,5x8 mm	Ti	2x TiO2 on one side and symfysis, 2x machined on the other	Bone level	5: 2 in each side and 1 in the symphysis	2-stage	NA	6m+0	No	Yes on the overload side	NA	(A) On overload side after fixing the prosthesis	(A) 18m	(A) Brush once/ week. Gentel mechanical cleaning

																							of pockets 1/ month
24. Persson et al.	1996	Dog, Labrador	TREATMENT STUDY	5	Mandible	PM3, PM4, M1 on both sides of the mandible	3m	Split mouth	Screw type, Brånemark, Nobel Biocare, Göteborg, Sweden	3,75x10 mm	Ti	U	Bonelevel	6: 3 on each side	2 stage	-	3m+3m	No	-	1 month after ligature removal: Amoxicillin 375mg x2 for 3w	(1) After abutment surgery	(1) 3m	(1) tooth and abutment cleaning 3t /w.
25. Schou et al	1996	Monkey, Macaca Fascicularis	Marginal inflammation round implants vs ankylosed teeth – Microbiologic study	8	Mandible	M1, M2 on both sides	12w	Split mouth, ligatures on one side (ligature vs no ligature). Upper jaw vs lower jaw (implant, vs tooth)	Cylindrical, custom made. Collar on ligature implants for ligature retention	2,5 x 10 mm	Ti-coated polycarbonate	Ti coated polycarbonate	Tissuelevel	4: 2 on each side	1 stage	-	12w + 0	YES	No	Streptomycin - benzylpenicillin (Streptocillin Vet., 200.000 IE/ml, 1 ml), 2h preop. And once a day for 10 d. post op	(1) 2w before and 2w after extractions (2) 2w before and 8w after implant surgery	(1) 4w (2) 10w	(1) Brush, 2% CHX solution 3 min, 3t/ w. (2) same as 1
26. Fritz et al	1997	Monkey (Macaca mulatta)	Plate-from vs root-form implants vs natural teeth	36	Mandible, both sides	All mandibular molars	6m	Comparison between animals	Plate-form Screw-shaped (Osseodent. Collagen Corporation, Palo Alto CA. According to Fritz 1994)	Screw-shaped: 3,75x7 Plate form: 14x7x1,6	Ti	Na	Bone level	12 natural teeth (2nd molar), 11 plate-form (2nd molar region, 12 root form implants (2nd molar egion)	2-stage	-	6m+12m	Yes.	Yes, Fixed bridge placed min 3 m after implantation	Penicillin G procaine, 300,000 post operatively	At the time of suprastructure connection, min 6 months after implant installation	12m	Monthly cleaning, polishing of implants and teeth and implant scaling with plastic scaler
27. Hanisch et al	1997	Monkey, Macaca Mulatta	Experimental peri-implantitis	4	Maxilla and mandible	All premolars	3m	Maxilla vs mandible	Cylindrical	NAx10 Bio-vent Dentsply	Ti	HA-coated	Bone level	8: 2 in each quadrant	2-stage	NA	12m+5m	No	NA (no suprastructure)	NA	(A) 2m after abutment connection (B) 11m after baseline (1m afterligature removal)	(A) 3m (B) 4w	(A) Brusch + 2% CHX daily (B) Brusch+ 2% CHX 3times/w
28. Hürzeler et al	1997	Dog, Beagle	TREATMENT STUDY 6 different treatments	7	Mandible	All mandibular PMs and M1	3m	Ligatures around all implants	Screw shaped	3,75x7 mm Brånemark	Ti	U	Bone Level	6: 3 on each side of the mandible	2-stage	NA	3m+2w	Yes, according to treatment	No	Yes, after ligature removal. systemic Metronidazole hydrochloride 250mg daily for 3w	(A) After ligature removal (B) After Peri-implantitis surgery	(A) 2w (B) 3w	(A) Daily brushing with flour of pumice mixed with 0,12% CHX + 0,12% CHX spray. (B) 0,12% CHX spray

29. Isidor	1997	Monkey, Macaca Fascicularis	Overload vs Ligature	4	Mandible	M1, all PMs, all Is	8m	Split mouth	Screw shaped	3,5x8 mm	Ti	2x TiO2 on one side and symfysis, 2x machined on the other	Bone level	5: 2 in each side and 1 in the symphysis	2-stage	NA	6m+0	No	Yes on the overload side	NA	(A) On overload side after fixing the prosthesis	(A) 18m	(A) Brush once/ week. Gentel mechanical cleaning of pockets 1/ month
30. Isidor	1997	Monkey, Macaca Fascicularis	Overload vs Ligature	4	Mandible	M1, all PMs, all Is	8m	Split mouth	Screw shaped	3,5x8 mm	Ti	2x TiO2 on one side and symfysis, 2x machined on the other	Bone level	5: 2 in each side and 1 in the symphysis	2-stage	NA	6m+0	No	Yes on the overload side	NA	(A) On overload side after fixing the prosthesis	(A) 18m	(A) Brush once/ week. Gentel mechanical cleaning of pockets 1/ month
31. Saito et al.	1997	Dog, Mongrel	Effects of long-term undisturbed plaque formation on peri-implant tissues. 2 implant systems vs teeth	4	Mandible	PM3, PM4 on both sides	3m	Split mouth	Screw type, (BR) Brånemark and Cylinder type, (IN) Integral	NA	Ti	NA	Bone level	4: 2 on each side	2 stage	-	2m+1m	No	Yes, functional loading by means of a bridge	Single dose of Mycillin at implant placement and abutment connection respectively	(1) after abutment connection	(1) 1m	(1) brush and 0,12 CHX 1t /w.
32. Tillmans et al	1997	Dog, Beagle	Bone loss around 3 differrent implant systems with and without ligatures	16	Mandible	PM2, PM3, PM4 On both sides	3m	Inter-quarant – 1 of each implant type on each side	(a) cylindrical (b) cylindrical (c) screw-form	(a)-(c) 4x 10 mm	(a)-(c) Ti-6A1-4V	(a) Calcitite HA coating (Sulzer Calcitek, Carlsbad, CA) (b) Commercially pure TPS coating (APS Materials, Dayton OH) (c) Machined Ti-alloy surface (Sulzer, Calcitek)	Bone level	6: 3 on each side	2-stage	NA	3m+4w	Yes, one implant of each type randomly placed on each side of the mandible<br>Ligature side also randomly selected	No – suprastructure on neitghbouring teeth used to prevent implant loading	NA	(A) After abutment surgery	(A) 1m on ligature side. 4m on control side (until sacrifice)	(A) Brushing, interproxmal brushing and scaling with graphite scaler 3t/w. With sedation every 2w when necessary
33. Abrahamsson et al.	1998	Dog, Beagle	3 implant systems: 1. Astra tech Implants, Dental System. 2. Nobel biocare, Brånemark System. 3. ITI dental implants system	5	Mandible	All mandibular premolars. (1st 2nd and 3rd maxillary molars also extracted to avoid occlusal	3m	One of each fixture in each quadrant in a randomized order	Screw-type	1: 3,5x8 2: 3,75x7 3: 4x8	Ti	1: NA (TiOblast?) 2: NA (TiUnite?) 3: TPS	1 and 2: Bonelevel 3: Tissuelevel	6	1-2: 2-stage (submerged) 3: 1-stage (tissuelevel)	NA	3m + 1m	Yes	No	NA	After implant installation	4m	Daily brushing and dentifrice

						interferen ce)																	
34. Eke et al	1998	Monkey, Macaca mulatta	Microbiota around teeth and implants (plate-form, root shape)	6	Mandible, both 1st molar regions	All mandibula r molars	6m	Comparison between animals	Plate-form Screw-shaped (Osseodent. Collagen Corporation, Palo Alto CA. According to Fritz 1994)	Screw-shaped: 3,75x7 Plate form: 14x7x1,6	Ti	NA	Bone level	1-2 (5 natural molars, 5 plate form and 2 screw-shaped totally in 6 monkeys)	2-stage	Only 1 implant per side, distal from the second premolar	6m+12m	NA	Yes, Ligated tooth/ implant used as distal end in suprastruct ure placed min. 6 months after implant insertion	As indicated	At the time of suprastructure connection, min 6 months after implant installation	12m	Monthly cleaning, polishing of implants and teeth and implant scaling with plastic scaler
35. Hürzeler et al	1998	Monkey, cynomol gus	Repetitive mechanical trauma ± ligature	5	Mandible	All mandibula r PM:s and M1, M2	12w	Ligatures on one side of the mandible	Screw shaped	3,75x7 mm Brånemar k	Ti	U	Bone level	8:4 on each side of the mandible	2-stage	NA	16w+4w	Yes ligature side chosen by flipping a coin	Yes, single crowns	-	(A) First week after abutment (B) Second week after abutment	(A) 1w (B) 3w	(A) 2% CHX swabbing 3 times/ w (B) Brush + flour of pumice and 2% CHX spray 3 times/ w.
36. Isidor	1998	Monkey, Macaca Fascicul aris	Overload vs Ligature	4	Mandible	M1, all PMs, all Is	8m	Split mouth	Screw shaped	3,5x8 mm	Ti	2x TiO2 on one side and symfysis, 2x machined on the other	Bone level	5: 2 in each side and 1 in the symphysis	2-stage	NA	6m+0	No	Yes on the overload	NA	(A) On overload side after fixing the prosthesis	(A) 18m	(A) Brush once/ week. Gentel mechanica l cleaning of pockets 1/ month
37. Miyata et al	1998	Monkey, Macaca fascicula ris	Overload during 0, 1, 2, 3, 4 and 5 weeks	5	Mandible, right side	PM2, M1 on the right side of the mandible	3m	Comparison between animals	Cylindrical (IMZ, Friatec, Mannheim, Germany)	2,8x8 mm	Ti	NA	Bone level	2 on the right side of the mandible	2-stage	NA	3m + 2w	No.	Yes – overload	NS	Hygiene start up point not specified	During the whole test phase	Cleaning 1/w under general anesthesia
38. Tillman ns et al	1998	Dog, Beagle	Bone loss around 3 differrent implant systems with and without ligatures	16	Mandible	PM2, PM3, PM4 On both sides	3m	Inter-quarant – 1 of each implant type on each side	(a) cylindrical (b) cylindrical (c) screw-form	(a)-(c) 4x 10 mm	(a)-(c) Ti-6A1-4V	(a) Calcitite HA coating (Sulzer Calcitek, Carlsbad, CA) (b) Commercially pure TPS coating (APS Materials, Dayton OH) (c) Machined Ti-alloy surface (Sulzer, Calcitek)	Bone level	6: 3 on each side	2-stage	NA	3m+4w	YES, one implant of each type randomly placed on each side of the mandible Ligature side also randomly selected	No – suprastruct ure on neithbourin g teeth used to prevent implant loading	NA	(A) After abutment surgery	(A) 1m on ligature side. 4m on control side (until sacrifice)	(A) Brushing, interprox mal brushin and scaling with graphite scaler 3t/w. With sedation every 2w

																							when necessary
39. Machad o et al	1999	Dog, Mongrel	TREATMEN T STUDY	4	Mandible	PM2, PM3, PM4 On both sides the mandible	3	Ligatures around all implants, then 4 different treatments	Screw shaped	8,5x3,75 mm	Ti	?	Bone level	4: 2 on each side of the mandible	2-stage	NA	3m+2w	(Yes but according to TREATMENT)	No	No (Metronidaz ole 250mg/day for 3w after ligature removal)	(A) After ligature removal before treatment (B) After PI treatment	(A) 2w (B) 5m	(A) Brusch and CHX 0,12% daily (B) CHX daily
40. Persson et al.	1999	Dog, Beagle	TREATMEN T STUDY	4	Mandible	PM1, PM2, PM3, PM4 on each side	4m	Different positions (inter-quadrant)	Screw type, Brånemark, Nobel Biocare, Göteborg, Sweden	7 mm leangth	Ti	U	Bonelevel	6: 3 on each side	2 stage	-	4m+2m	No	-	1m after ligature removal,(Ima cillin 250 mg x2) for 3w	(1) After abutment surgery	(1) 2m	Tooth and abutment cleaning every 2nd day
41. Wetzel et al	1999	Dog, Beagle	TREATMEN T STUDY Guided tissue regeneration with 4 differeent surfaces	7	Mandible	PM2, PM3, PM4	5m	Interquadra nt distribution of 4 different surfaces	Hollow cylinder (ITI,	2,8 x6 mm	Ti	(1) TPS (2) SLA (3) U (4) TPS with perforation to mimic a furcation	Tissue level	41 implants in total	1-stage	NA	3m+0	No	NA – no suprastruct ure	During treatment	(a) after tooth extraction until induction of PI	(a) 8m	(a) tooth polish with rubber cup and pumice + 0,12% CHX 3t/w.
42. Comut et al	2000	Dog, Beagle	3 implant surfaces A-C	4	Mandible, implants on both sides.	4 premolars and 1 molar on each side of mandible	3m	Inter-quadrant	Screw shaped ITI	8x3 mm (top 2 mm was a trasmucos al part)	Ti	Identical surfaces except the apical 1 mm of the transmucosal part: A: Machined B: HA-plasma sprayed C: HA-ion beam assisted deposition	Tissue level	9-10 (39 total in all 4 animals, 4-5 per quadrant)	1-stage	NA	3+0	Yes	NA (although no suprastruct ure was used)	NA	2w after implant installation	3m minus 2w	CHX rinse (0,12%) for 2 weeks after implant insertion. Daily brushing with 0,2% CHX gel thereafter.
43. Machad o et al	2000	Dog, Mongrel	TREATMEN T STUDY	5	Mandible	PM2, PM3, PM4 On both sides the mandible	3	Ligatures around all implants, then 4 different treatments	Screw shaped	8,5x3,75 mm	Ti	?	Bone level	4: 2 on each side of the mandible	2-stage	NA	3m+2w	(Yes but according to TREATMENT)	No	No (Metronidaz ole 250mg/day for 3w after ligature removal)	(A) After ligature removal before treatment (B) After PI treatment	(A) 2w (B) 5m	(A) Brush and CHX 0,12% daily (B) CHX daily
44. Miyata et al	2000	Monkey, Macaca fascicula ris	Overload supraocclusio n 100 µm 180 µm 250 µm	4	Mandible, right side	PM2, M1 on the right side of the mandible	3m	Comparison between animals	Cylindrical (IMZ, Friatec, Mannheim, Germany)	2,8x8 mm	Ti	NA	Bone level	2 on the right side of the mandible	2-stage	NA	3m + 2w	No.	Yes – overload	NS	Hygiene start up point not specified	During the whole test phase	Cleaning 1/w under general anesthesia
45. Nocitiet al.	2000	Dog, Mongrel	TREATMEN T STUDY GBR, bone substitute or both	5	Mandible	PM2, PM3, PM4 one both sides	3m	Each defect randomly allocated to one of 4 treatments	Screw-type (Napio System, Napio, Bauru, Brazil)	3.75x8.5	Ti	AE	Bonelevel	4: 2 on each side	2 stage	NA	3m+2w	Yes – according to treatment	No	After ligature removal: Metronidazol e 250mg/ d for 3w	(1) after ligature removal (2) After peri-implantitis surgery	(1)2w (2) 5m	(1)Brush + 0,12% CHX daily (2) 0,12 CHX

																							spray topically 2t/d.
46. Shibutani et al	2000	Dog, Beagle	Ligature induced bone loss with-without IV Pamidronate	10	Mandible	PM2, PM3 on the left side	6m	Comparison between animals	Screw shape (TiOblast, Astra Tech AB, Mölndal, Sweden)	3,5x 11 mm	Ti	TiOblast	Bone-level	2 per animal	2-stage	NA	4,5m+3w	NA	No	NA	(A) After abutment connection	(A) 3w	(A) Brush and 0,12% CHX rinse 1t/ w.
47. Deppe et al	2001	Dog, Beagle	TREATMENT STUDY	6	Mandible, both sides	NS	NA	1 of 3 treatment methods in each hemimandible	Cylindrical (Frialit 2)	3,8x11	Ti	Ti plasma spray coated	Bone Level	10 5 on each side)	2-stage	NA	3m+4w	No	NA (no suprastructure	NA	At abutment connection	4w	Oral hygiene
48. McCracken et al	2001	Monkey, Macaca Mulatta	Plate-from vs root-form implants vs natural teeth	36	Mandible, both sides	All mandibular molars	6m	Comparison between animals	Plate-form Screw-shaped (Osseodent. Collagen Corporation, Palo Alto CA. According to Fritz 1994)	Screw-shaped: 3,75x7 Plate form: 14x7x1,6	Ti	Na	Tissue level	24 natural teeth (2nd molar), 24 plate-form (2nd molar region, 24 root form implants (2nd molar egion)	1-stage	Abutment center 12 mm distal to the 2nd PM	Ligatures placed after 3m healing + 2y loading	Yes.	Yes, Fixed bridge placed min 3 m after implantation	Penicillin G procaine, 300,000 post operatively	At the time of suprastructure connection, 3m after implant installation	24m	Monthly cleaning, polishing of implants and teeth and implant scaling with plastic scaler
49. Nociti et al.	2001a	Mongrel dogs	Absorbable vs. nonabsorbable membrane in GBR	5	Mandible	8: 4 each side (1ºPM, 2ºPM, 3ºPM, 4ºPM)	12w	Split-mouth	Screw-type (Napio System, Napio, Bauru, Brazil)	3.75x8.5	Ti	AE	Tissuelevel (bonelevel?)	6	2 stage	NA	12w + 2w	Yes	No	At implant surgery (one time), after ligatures removal (3w), after GBR (1w)	(1) After abutment, (2) after removal of ligatures, (3) after GBR	(1) 2w (2) 3w (3) 5m	(1) NA (2) daily brushing + CHX (3) CHX 2x/day
50. Nociti et al.	2001b	Mongrel dogs	Teeth vs. implants	5	Mandible	6: 3 each side (2ºPM, 3ºPM, 4ºPM)	12w	Split-mouth	Screw-type (Napio System, Napio, Bauru, Brazil)	3.75x8.5	Ti	NA	Tissuelevel (bonelevel?)	4	2 stage	NA	12w + 2w	No	No	NA	After abutment	2w	daily brushing + CHX
51. Nociti et al.	2001c	Mongrel dogs	Absorbable vs. nonabsorbable membrane in GBR	5	Mandible	8: 4 each side (1ºPM, 2ºPM, 3ºPM, 4ºPM)	12w	Split-mouth	Screw-type (Napio System, Napio, Bauru, Brazil)	3.75x8.5	Ti	AE	Tissuelevel (bonelevel?)	6	2 stage	NA	12w + 2w	Yes	No	after ligature removal (3w), after GBR (1w)	(1) After abutment, (2) after GBR	(1) 2w (2) 5m	(1) daily brushing + CHX (2) CHX 2x/day
52. Nocitiet al	2001d	Dog, Mongrel	TREATMENT STUDY of 4 treatments	5	Mandible	All PMs on both sides	3m	Each defect randomly allocated to one of 4 treatments	Screw-type (Napio System, Napio, Bauru, Brazil)	3.75x8.5	Ti	AE	Bonelevel	4: 2 on each side	2 stage	NA	3m+2w	Yes – according to treatment	No	After ligature removal: Metronidazole 250mg/ d for 3w	(1) after ligature removal (2) After peri-implantitis surgery	(1)2w (2) 5m	(1)Brush + 0,12% CHX daily (2) 0,12 CHX spray topically 2t/d.

53. Persson et al.	2001a	Beague dogs	Turned x SLA surface	4	Mandible	8: 4 each side (1ºM, 2ºPM, 3ºPM, 4ºPM)	12m	Split-mouth	Screw-type (ITI Straumann, Waldenburg, Switzerland)	3.3x8.0	Ti	U (left side) SA (right side)	Tissuelevel	6	1 stage	-	3m + 0	No	-	5 weeks after removal of ligatures, for 17 days	(1) After implant installation; (2) 1 month after treatment	(1) 3m (2x/week); (2) 6m	(1) and (2) Tooth and implant cleaning
54. Persson et al.	2001b	Labrador dogs	1 part x 2 parts implant	2	Mandible	10: 5 each side (1ºPM, 2ºPM, 3ºPM, 4ºPM, 1ºM)	3m	Different positions	Screw-type (Brånemark, Nobel Biocare, Göteborg, Sweden)	10 mm	Ti	U	Bonelevel	8	2 stage	-	4m + 5m	No	-	15 months after ligature, for 3 weeks	NP	NP	NP
55. Schou et al	2001	Monkey, Macaca Fascicul aris	Probe tip position around implants vs teeth at health, mucositis and peri-implantitis	8	Mandible and Maxilla	M1 OR PM1& PM2	3m	Split mouth	Screw type, Astra tech with customized transmuosal part	7x4 mm + 4 mm transmuco sal part.	Ti	U	Tissuelevel	4: 1 in each quadrant	1 stage	-	3m+0	YES and blinded observation	No	Streptomycin - benzylpenicil lin (Streptocillin Vet., 200.000 IE/ml, 1 ml), 2h preop. And once a day for 10 d. post op	(1) 2w before and 2w after extractions (2) 2w before implant placement until 3m after	(1) 4w (2) 2w+3m	(1) and (2) Brush, curretage, 1% CHX solution 3 min, 3t/ w.
56. Deppe et al	2002	Dog, Beagle	TREATMEN T STUDY Ti release related to CO_2 laser treatment	6	Mandible, both sides	NS	NA	Split mouth	Cylindrical (Frialit 2)	3,8x11	Ti	Ti plasma spray coated	Bone level	10 (5 on each side)	2-stage	NA	3m+4w	No	NA (although no suprastruct ure was used)	NA	At abutment connection	4w	Oral hygiene
57. Gotfreds en et al	2002	Dog, beagle	Loading vs non-loading. SLA vs turned surface	5	Mandible, both sides	8: 4 in each side of the mandible (PM1, PM2, PM3, PM4)	12w	Split-mouth	Screw-shape (Straumann tissue level)	3,3x8	Ti	(SLA) sandblasted and acid-etched<br><br>(TURNED)	Tissue level	6: 3 on each side	1-stage	NA	0+12w	No	Yes	NA	2w after implantation	10w	Brush and CHX gel 1% daily
58. Miyata et al	2002	Monkey, Macaca fascicula ris	Overload	4	Mandible, right side	PM2, M1 on the right side of the mandible	3m	Comparison between animals	Cylindrical (IMZ, Friatec, Mannheim, Germany)	2,8x8 mm	Ti	NA	Bone level	2 on the right side of the mandible	2-stage	NA	3m + 2w	No.	Yes – overload	NS	Hygiene start up point not specified	During the whole test phase but varying for different implant. See table 2	Cleaning 1/w under general anesthesia
59. Schou et al	2002	Monkey, Macaca Fascicul aris	TREATMEN T STUDY	8	Mandible and Maxilla	Some Ms and PMs	3m	Different treatments in all quadrants	Screw type, Straumann Tissue level, custom type	2.8 x 8 mm + 3 mm transmuco sal part	Ti	TPS at bone part and smooth transmucosal part	Tissuelevel	8: 2 in each quadrant	1 stage	-	3m+0	YES, according to treatment	No	Metronidazol e 13 mg/ kg x3/ d and ampicillin 17mg/ kg x3/ d. From 2d before surgery to 10d after.	Yes, but not further specified. Terminated at ligature placement	NA	NA

60. Schou et al	2002	Monkey, Macaca Fascicularis	TREATMENT STUDY	8	Mandible and Maxilla	Some Ms and PMs	3m	Different treatments in all quadrants	Screw type, Straumann Tissue level, custom type	2.8 x 8 mm + 3 mm transmucosal part	Ti	TPS at bone part and smooth transmucosal part	Tissuelevel	8: 2 in each quadrant	1 stage	-	3m+0	YES, according to treatment	No	Metronidazole 13 mg/ kg x3/ d and ampicillin 17mg/ kg x3/ d. From 2d before surgery to 10d after.	After implant surgery	3m	Cleaning 3t/ w.
61. Schou et al	2002	Monkey, Macaca Fascicularis	TREATMENT STUDY: Autogenous bone graft and ePTFE	8	Mandible and Maxilla	PM1, PM2 and M2 in each quadrant	3m	Different treatments in all quadrants	Screw type, Straumann Tissue level, custom type	2.8 x 8 mm + 3 mm transmucosal part	Ti	TPS at bone part and smooth transmucosal part	Tissuelevel	8: 2 in each quadrant	1 stage	-	3m + 0	YES	No	Yes, Metronidazole during PI TREATMENT	(1) 2w before and until 3m after extractions	(1) 4w	(1) Brush, 2 % CHX solution 3 min, 3t/ w.
62. Schou et al	2002	Monkey, Macaca Fascicularis	TREATMENT STUDY: Autogenous bone graft and ePTFE	8	Mandible and Maxilla	PM1, PM2 and M2 in each quadrant	3m	Different treatments in all quadrants	Screw type, Straumann Tissue level, custom type	2.8 x 8 mm + 3 mm transmucosal part	Ti	TPS at bone part and smooth transmucosal part	Tissuelevel	8: 2 in each quadrant	1 stage	-	3m + 0	YES	No	Yes, Metronidazole during PI TREATMENT	(1) 2w before and until 3m after extractions	(1) 4w	(1) Brush, 2 % CHX solution 3 min, 3t/ w.
63. Shibli et al	2003	Dog, Mongrel	Microbiology and bone loss after ligatures around 4 different implants	6	Mandible	All mandibular and maxillary PMs	3m	Inter-quadrant	Screw-shape (1) Commercially pure Ti (3i) (2) TPS (ITI/ Straumann, Esthetic plus (3) HA-coated (Calcitek) (4) Hybrid: first 3 threades machined, the rest acid etched (Osseotite, 3i)	(1), (3), (4): 3,75 x 10 mm<br>(2) 4,1 x 10 mm	Ti	(1) Turned (3i) (2) TPS (ITI/ Straumann, Esthetic plus (3) HA-coated (Calcitek) (4) Hybrid: first 3 threades machined, the rest acid etched (Osseotite, 3i)	Bone-level	6: 3 on each side	2-stage	NA	90d + 45d	Yes, at least one implant of each kind in each animal	No	Potassium and sodium benzyl penicillin once/w for 2w postoperatively	(A) 2w before extraction<br>(B) After tooth extraction	(A) at one time<br>(B) 225d (until 45 days after abutment connection)	(A) Oral hygiene<br>(B) Scrubbing with 0,12 % CHX daily and scaling and root planning 1t/m.
64. Shibli et al	2003	Dog, Mongrel	TREATMENT STUDY	6	Mandible	All PMs on both sides	3m	Inter-quadrant	Screw-shape (1) Commercially pure Ti (3i) (2) TPS (ITI/ Straumann, Esthetic plus (3) HA-coated (Calcitek) (4) Hybrid: first 3 threades machined, the rest acid etched (Osseotite, 3i)	NA	Ti	(1) Turned (3i) (2) TPS (ITI/ Straumann, Esthetic plus (3) HA-coated (Calcitek) (4) Hybrid: first 3 threades machined, the rest acid etched (Osseotite, 3i)	Bonelevel	19 implants in this study but 36 implants installed	2-stage	NA	3m+2m	NA	NA – although no suprastructure	NA (peri-operative drug administration not specified)	(A) after abutment connection (B) After ligature removal	(A) 2m (B) 12m	(A) not specified (B) daily scrubbing with 0,12% CHX
65. Shibli et al	2003	Dog, Mongrel	TREATMENT STUDY: Lethal photosensitization and GBR	6	Mandible	All mandibular and maxillary PMs	3m	No control group – all implants received the same treatment	Screw-shape (1) Commercially pure Ti (3i) (2) TPS (ITI/ Straumann, Esthetic plus	(1), (3), (4): 3,75 x 10 mm<br>(2) 4,1 x 10 mm	Ti	(1) Turned (3i) (2) TPS (ITI/ Straumann, Esthetic plus (3) HA-coated (Calcitek)	Bone-level	6: 3 on each side	2-stage	NA	90d + 45d	Yes, at least one implant of each kind in each animal	No – upper PMs extracted to avoid loading	Potassium and sodium benzyl penicillin once/w for 2w	(A) 2w before extraction<br>(B) After tooth extraction	(A) at one time<br>(B) 225d (until 45 days after	(A) Oral hygiene<br>(B) Scrubbing with 0,12 % CHX

									(3) HA-coated (Calcitek)<br>(4) Hybrid: first 3 threades machined, the rest acid etched (Osseotite, 3i)			(4) Hybrid: first 3 threades machined, the rest acid etched (Osseotite, 3i)								postoperatively	(C) After ligature removal	abutment connection)<br><br>(C) 12m	daily and scaling and root planning 1t/m.<br><br>(C) same as (B)
66. Zechner	2003	Dog, Mongrel	(A) One stage and two stage surgery compared<br>(1) 1-stage with integrated abutment<br>(2) 1-stage with screw retained abutment<br>(3) 2-stage with screw retained abutment<br>(B) Ligature vs no ligature	8	Mandible	All PMs and M1 on both sides of the mandible	3m	Interquadrant (implants and technique) and split mouth (ligatures on one side)	Cylinder type – Custom made by Friatec, Friedrichsfeld, Germany	All 3,5 x10 mm	Ti	TPS and 1mm polished collar	(1) Tissue level<br>(2) and (3) Bone level	48 in total, but only 23 reported in this study	(1) and (2) 1-stage<br>(3) 2-stage	NA	(3) 3m+1m<br>(1) & (2) 4m+0	Yes, random placement of 3 different implant types	Functional loading by means of high plastic healing caps	NA	(A) after abutment surgery	(A) 1m (2-stage) and 4m (1-stage)	(A) Brush with 0,2% CHX gel 3-4t/ w and 0,12% CHX rinse every 2w.
67. Deppe et al	2004	Dog, Beagle	Assessment of reliability of 3 bone attachment measurement methods	6	Mandible, both sides	NS	3m	Reliability of 2 X-ray methods and pressure-forced probing,	Cylindrical (Frialit 2)	3,8x11	Ti	Ti plasma spray coated	Bone level	10 (5 on each side)	2-stage	NA	12w+4w	NA	NA (no suprastructure)	NA	At abutment connection	4w	Oral hygiene
68. Martins et al.	2004	Dog, Mongrel	4 surfaces	6	Mandible	8 premolars (4/side). (maxillary also extracted to avoid occlusal trauma interference)	3m	≥ one of each implant in each animal	Screw-type	3,75x10<br>4,1x10	Ti	TPS, HA, U + AE (U in 3 first threads and AE in other threads), U	Bone level	6: 3 on each side of the mandible	2 stage (submerged)	10	3m + 45d	No	No	24000 IU/kg benzylpenicillum 1x/week for 2 weeks after implant placement	Before PI	9,5 (2 weeks before extraction to ligature placement.)	Daily chlorhexidine scrubbing + Scaling and root planning 1x/month
69. Persson et al.	2004	Beague dogs	Turned x SLA surface	4	Mandible	10: 5 each side (1^oPM, 2^oPM, 3^oPM, 4^oPM, 1^oM)	3m	Split-mouth	Screw-type (ITI Straumann, Waldenburg, Switzerland)	3.3x8.0	Ti	U (left side)<br>SA (right side)	Tissuelevel	6	1 stage	-	3m + 0	No	-	1 month after removal of ligatures, for 17 days	(1) After implant installation; (2) 5 weeks after treatment	(1) 3m (2x/week); (2) 5m	(1) and (2) Tooth and implant cleaning

70. Zitzmann et al	2004	Dog, Labrador	Progression av PI after ligature removal	5	Mandible	All PMs snd M1 on both sides	3m	Ligatures around all implants	Screw type (Brpnemark, Nobel biocare)	3,75 x10 mm	Ti	NA	Bone level	22 implants in 5 dogs	2-stage	NA	4m+5m	No	NA-no suprastructure	NA	(A) after abutment surgery	(A) 5m	(A) Brusch and dentifrice 5t/w
71. Hayek et al	2005	Dog, Labrador Retriever	TREATMENT – Photodynamic - vs conventional therapy	9	Mandible	PM3 on both sides	0(immediate installation)	Split mouth	Screw-shaped	NA Conexao System, Sao Paolo Brazil	Ti	AE	Bone level	2: 1 in each side of the mandible	2-stage	-	3m+0m (PI induction and abutment connection at same time)	Yes-according to treatment	NA (no suprastructure)	NA	None (only submerged healing)	-	-
72. Martins et al	2005	Dog, Mongrel	4 surfaces	6	Mandible	8 premolars (4/side). (maxillary also extracted to avoid occlusal trauma interference)	3m	≥ one of each implant in each animal	Screw-type	3,75x10 4,1x10	Ti	TPS, HA, U + AE (U in 3 first threads and AE in other threads), U	Bone level	6: 3 on each side of the mandible	2 stage (submerged)	10	3m + 45d	No	No	24000 IU/kg benzylpenicillum 1x/week for 2 weeks post op (after implant placement – OTHERWISE, POST-OP. OF WHICH SURGERY? THIS MIGHT BE CONFUSING FOR THE READER IF NOT INDICATED	(A) Before PI (B) After PI	(A) 9,5 (2 weeks before extraction to ligature placement.) (B) 12m	(A) Daily chlorhexidine scrubbing + Scaling and root planning 1x/month (B) same as A
73. Sennerby et al.	2005	Dog, Beagle	Implant stability during initiation and resolution of exp. Periimplantitis	4	Mandible	8: PM1, PM2, PM3, PM4 on both sides	12m	Split mouth	Screw type (ITI Dental implant system, Straumann AG, Waldenburg, Switzerland)	3,3x8	Ti	Sand-blasted, acid etched (SLA) or U	Tissue level	6: 3 on each side	1-stage	NA	3m+0	No, SLA on right side. U on left side.	NA – no suprastructure used	NA – No specification of perioperative drugs	(A) After implant insertion	(A) 3m	(A) Implants and teeth cleaned 2t /w.
74. Stübinger et al	2005	Dog, Bealge	TREATMENT STUDY CO2 LASER	6	Mandible	NA (molar and premolar region)	NA	1 of 3 different treatments per hemimandible	Cylindrical (stepped) Frialit-2 (Friadent, Mannheim, Germany)	3,8 x11 mm	Ti	TPS	Bonelevel	10: 5 on each side	2-stage	NA	3m+0	No	NA	NA	(A) After implant surgery	(A)3m	(A) oral hygiene, not further specified
75. Trejo et al	2005	Monkey, Cynomolgus	Effect of different hygiene techniques on mucositis and CAL. 3 maintenace protocols after ligature removal	9	Mandible	PM2, M1, M2 on both sides	4m	Comparison between animals	Screw-shape (ITI, tissue level)	3,3x 8 mm	Ti	Ti plasma coated	Tissue level	4: 2 on each side	1-stage	At least 3 mm between implants and adjacent teeth	90d+0d	Yes, monkeys randomly distributed to different treatments	No – cover screws not used in order to avoid contact	NA	(A) After implant surgery	(A) 90d	(A) 0,12% CHX spray and brushing every 2d

76. Watzak et al	2005	Monkey, baboon (Papio ursinus)	Bone loss around 3 different implant types after 18m functional loading without oral hygiene	9	Maxilla and mandible	M1, M2 on both sides of maxilla and mandible	7m	Split mouth: 1-implant type per quadrant. All implant designs used in all animals	(1) Screw shaped (Brånemark MKII, Nobel Biocare Göteborg, Sweden) (2) Screw shaped (Frialen 20-0340, Friatec Mannheim, Germany) (3) Cylindrical (Frialen 20-0140, Friatec)	All 3,75 mm diameter.<br>All 10-13 mm leangth	(1) Ti (2) Ti (3)	(1) U (Ra 0,53 micrometer) (2) Sandblasted + AE (Ra 2,1 micrometer) (3) TPS	Bone level	12: 3 in each quadrant	2-stage	NA	8m+1m (induction by plaque accumulation only)	No	Yes – functional loading assured by fixted partial denture splinting all 3 implants in each quadrant	No	NA	NA	NA
77. Berglundh et al	2006	Dog, Beagle	2 surfaces	5	Mandible, both premolar regions	All mandibular premolars	3m	Split mouth	Screw shaped. Straumann SP	3,3x8	Ti	Sandblasted Acid Eched Vs Polished	Tissue level	6	1-stage	NA	3 months and 2 weeks + 0	No	No (Not specifically stated, no extraktion of antagonizing teeth reported)	NA	2w after implant installation	3m minus 2w	Daily cleaning of implants and teeth with toothbrush and 0,12% CHX gel.
78. Kozlovsky et al	2006	Dog, Beagle	Overloading in healthy or inflamed peri-implant tissue	4	Mandible	8: PM1 PM2 PM3 PM4 On both sides	3m	Ligatures on one side. 2 posterior implants loaded and 2 anterior implants unloaded on each side	Screw shaped Hi-Tec Implant Ltd., Herzliya, Israel	3,75x10 mm	Ti	U	Bone level	8: 4 in each side of the mandible	2-stage	NA	3m+3w	No	Yes	20mg/kg Benzanthine Penicillin G (2% chanazine) as pre-medication before implant placement.	(A) After abutment surgery (B) On control side after ligature placement on other side	(A) 3w (B) 12m	(A) 0,2% CHX swabbing 3 times/w (B) 0,2% CHX brushing 3 times/w
79. Schwarz et al	2006	Dog, Beagle	TREATMENT STUDY: Submerged vs non-submerged healing	5	Mandible, both sides	PM2, PM3, PM4, M1 on both sides	4m	Split mouth	Screw-shape (Straumann SP, NN, SLA,	3,3x10	Ti	SLA	Tissue level	6: 3 on each side	1-stage	8 mm apart	3m+0	Yes according to treatment, computer generated protocol	No	No	(A) after implant surgery	(A) 3m	(A) Brush daily
80. Shibli et al	2006	Dog, Mongrel	TREATMENT STUDY: Lethal photosensitization and GBR	5	Mandible	All PMs and M1 on both sides of the mandible	3m	Split mouth	Screw shape (1) CpTi Sterngold Implantmed, Attleboro, MA, USA) (2) TPS, Sterngold… (3) Hybrid – machined in first 3 screws and then acid etched (3i	3,75x10 mm	Ti	(1)Turned<br>(2) TPS,<br>(3) Hybrid – machined in first 3 screws and then acid etched<br>(4) Sandblasted with ti oxide	Bone level	8: 4 on each side	2-stage	NA	3m+2m	Yes according to treatment	No – upper PMs extracted to avoid loading	otassium and sodium benzyl penicillin once/w for 2w postoperatively	(A) After tooth extraction	(A) until ligature placement	(A) Scrubbing with 0,12 % CHX daily and scaling and root planning 1t/m.

									Osseotite, Implants Innovations (4) Sandblasted with ti oxide Porous, Conexao Implants…														
81. Schwarz et al	2007	Dog, beagle	Human vs dog: Defect size and configuration of natural vs ligature-induced PI	5 dogs and 24 human patients	Mandible, both sides	PM2, PM3, PM4, M1, On both sides	4m	Ligatures around all implants	Screw-shape (ITI, tissue level)	3,3 x 10 mm	Ti	Sand-blasted, acid etched (SLA)	Tissue level	6: 3 on each side	1-stage	NA	3m+0	-	No	Clindamycine 11mg/kg during tooth extraction surgery and for 10d after	(A) after implant surgery	(A) 3m	(A) Brush daily
82. Takasaki et al	2007	Dog, Beagle	TREATMENT STUDY Er:YAG vs curettage	4	Mandible	PM1, PM2, PM3, PM4 on both sides	3m	Split mouth	Screw-form (Standard plus, ITI dental Implant System, Straumann AG, Waldenburg, Switzerland)	3,3x 10 mm	Ti	Sand-blasted Large grit Acid-etched (SLA)	Tissue level	4:2 on each side	1-stage	NA	3m+0	No	NA (no suprastructure)	Penicillin G, 200.000 U/dayfollowing implant installation	(A) After implant insertion	(A) 3m	(A) Clinical plaque control with 2% CHX 3t/w.
83. You et al	2007	Dog, Mongrel	TREATMENT STUDY	6	Mandible	All PMs	3m	One of 3 different treatments per quadrant	Screw type (Osstem, Seoul, Kora)	4,1x 10 mm	Ti	AE	Tissue level	6: 3 on each side	1-stage	NA	3m+0	No	NA-no suprastructure	During treatment	NA	NA	NA
84. Albouy et al	2008	Dog, Labrador (same animals as study 6 in this table)	4 implants/ -surfaces (A, B, C, D)	6	Mandible, right side	12 (All mandibular premolars and the three anterior premolars in the maxilla on both sides)	3m	Interquadrant: One of each implant type in the same quadrant	Screw type A: Biomet 3i ICE Micro miniimplant B: Astra Tech MicroThreaded C: Straumann SP NN D: Nobel Biocare MKIII NP	A: 2,35x10 B: 3,5x11 C: 3,3x10 D: 3,3x10	Ti	A: U B: TiOblast C: Sandblasted AE D: TiUnite	A,B,D: Bonelevel C: Tissuelevel	4 (all in 1 quadrant)	1-stage	NA	3m + 0	Yes	No	NA	2w after implant installation	3m minus 2w	Daily cleaning of implants
85. Martines et al	2008	Dog, Beagle	2 surfaces – Implant mobility and clinical reaction to exp. PI	5	Mandible	PM2 PM3 PM4 On both sides of the mandible	90d	Random mesio-distal order	Screw shaped Turned Brånemark and Sandblastid acid/ etched (SLA) Straumann	4x11 mm (Brånemark) 4,1x10 mm (ITI, Straumann)	Ti	Brånemark: Turned Straumann: SLA (Sandblasted, acid-etched)	Coating level/ shoulder coinciding with the bone crest.	4: 2 on each side of the mandible	2-stage (?)	NA	120d+0d	YES Implants placed in randomized order	No	NA	(A) after tooth extraction	(A)210d	(A) Brushing with 0,12% CHX 3t/w
86. Albouy et al	2009	Dog, Labrador (same animals as study 7 in this table)	4 implants/ -surfaces (A, B, C, D)	6	Mandible, right side (4 implants on the left side presented in a separate study)	12 (All mandibular premolars and the three anterior premolars	3m	Interquadrant: One of each implant type in the same quadrant	Screw type A: Biomet 3i ICE Micro miniimplant B: Astra Tech MicroThreaded A: Straumann SP NN	A 2,35x10 B 3,5x11 C 3,3x10 D 3,3x10	Ti	A: U B: TiOblast C: Sandblasted AE D: TiUnite	A,B,D Bonelevel C Tissuelevel	4 (all in 1 quadrant)	1-stage	NA	3m + 0	Yes	No	NA	2w after implant installation	3m minus 2w	Daily cleaning of implants

						in the maxilla on both sides.)			B: Nobel Biocare MKIII NP C: Straumann SP NN D: Nobel Biocare MKIII NP														
87. Parlar et al.	2009	Mongrel dogs	Plaque control (intermediary piece of the implant)	9	Mandible	8: 4 each side (2ºPM, 3ºPM, 4ºPM, 1ºM)	3m	Split-mouth	Screw-type	4.1x13.0	Ti	SA	Tissuelevel	6	1 stage (however, with 3m healing)	NA	8w + 0	No	No	No	(1) Pre-experiment, (2) just before ligature removal, (3) after GBR	(1) 3w, (2) only once, (3) ?	(1) scaling, polishing, (2) brushing, local debrideme nt, CHX, (3) CHX spraying
88. Albouy et al	2011	Dog, Labrador	TREATMEN T STUDY 4 implants/ - surfaces (A, B, C, D)	6	Mandible, left side	12 (All mandibula r premolars and the 3 anterior premolars in the maxilla on both sides.)	3m	Interquadra nt: One of each implant type in the same quadrant	Screw type A Biomet 3i ICE Micro miniplant B Astra Tech MicroThread C Straumann SP NN D Nobel Biocare MKIII NP	A 3,25x10 B 3,5x11 C 3,3x10 D 3,3x10	Ti	A: U B: TiOblast C: Sandblasted AE D: TiUnite	A,B,D Bonelevel C Tissuelevel	4 (all in 1 quadrant)	1-stage	NA	3m + 0	Yes	No	NA	2w after implant installation	3m minus 2w	Daily cleaning of implants
89. Levin et al	2011	Dog, Mongrel	Reiimplantatio n of failed implants and new implants in failed sites	2	Mandible	All PMs on both sides of the mandible	12w		Screw shaped SEVEN, MIS implants	NA	Ti	?	Bone level	4: 2 on each side of the mandible	1-stage	NA (placed at PM1 and PM3 sites)	0+45d	No	NA (No suprastruct ure)	NA	NA	NA	NA
90. Schwarz et al	2011	Dog, beagle	TREATMEN T STUDY: Radiographica l and histological bone level after PI-srugery	6	Mandible, both sides	All PM1, PM2, PM3, PM4, M1, M2 In maxilla and mandible	10w	Split mouth according to treatment	Screw- shaped	3,8 x 11 mm (Camlog Screw line Implant, PromotePl us, Camlog Biotechno logies AG, Basel)	Ti	NA	Bone level (Implabnt shoulder 0,4 mm over the crest).	8: 4 on each side of the mandible	1-stage	10 mm apart	6w+0	Yes, according to treatment	No – upper teeth also extracted.	Clindamycin e 11mg/kg during tooth extraction surgery and for 10d after	(a) 7d after implant surgery (B) after ligature removal	(A) 6w (B) 4w	(A) brush 2t/ w. (B) Brush and dentifrice
91. Albouy et al	2012	Dog, Labrador	2 surfaces	5 according to abstract, 6 according	Mandible, one side	6 (All mandibula r premolars	3m	Interquadra nt: One of each in one side of the mandible	Screw type (Brånemark MKIII narrow platform)	3,3x10	Ti	Turned, TiUnite	Bone level	2	1-stage	NA	3m + 0	Yes	No	NA	2w after implant installation	3m minus 2w	Daily cleaning of implants

				to material and method		and the 3 anterior premolars in the maxilla on one side.)																	
92. Golubovic et al	2012	Dog, Beagle	CBCT vs histometric measurement of peri-implant bone loss	3 (6 in the original material)	Mandible, both sides	20:10 in each jaw. all PM1, PM2, PM3, PM4, M1, M2	10w	Evaluation of bone loss around 9 compromised sites in a material published in study 104 (Schwartz et al 2011)	Screw-shaped (Camlog Screw-line implant, Promote plus)	3,8x11	Ti	Promote plus (abrasive blasted, acid etched)	Bone level (implant shoulder exceeded the buccal aspect of the alveolar crest with 0,4 mm)	8; 4 on each side (However, 9 implants in the current study)	1-stage	10 mm	0+6w	Yes. According to treatment modality	No	?	(A) 1w after implantation (B) After ligature removal	(A) 5w (B) 4w	(A)Brush twice a week (B) Brush and dentifrice
93. López-Piriz et al	2012	Dog, Beagle	Soda-lime-glass/ Ag abutment coating vs control	5	Mandible	PM1 PM2 PM3 PM4 M1 On both sides of the mandible	3m	Interquadrant: Test implants at all central and distal sites.	Screw shaped Phibo dental solutions	NA	Ti	?	Bone level	6: 3 on each side of the mandible	2-stage	NA	2m+	No	NA (no suprastructure)	No	(A) After abutment connection	(A)4w	(A) Toothbrush and dentifrice 5d/w
94. Becker et al	2013	Mice	Ligature/ no ligature	8	Hard palate	None	-	Animals divided in 2 groups: A: Ligature group B: No ligature	Membrane tack (Friadent)	NA	Ti	NA	Tissue level	1	1-stage	-	0+0	No	No	NA	None	-	-
95. Carcuac et al	2013	Dog, Labrador (same animals as study 13 in this table)	Teeth vs implants/ 2 implant surfaces	6 according to material and method, 5 according to absctract	Mandible, implants on the right side, teeth on the left	Right mandibular premolars and first molar. Three right anterior maxillary premolars	3m	Split mouth (IMPLANTS VS TEETH) Interquadrant (2 SURFACES)	Screw shaped Nobel Biocare MKIII, NP	3,3x10	Ti	A: Turned B: TiUnite	Bone level	4 (On the right side of the mandible)	1-stage	NA	3+0	Yes – Pairwise placement	No	NA	2w after implant installation	3m minus 2w	Tooth and abutment cleaning 3 times a week
96. Fan et al	2013	Dog, Beagle	TREATMENT Different immunisation protocols: (A) plasmid vecor-rgpA (B) heat-killed P. gingivalis (C) Plasmid-vector (control)	15	Mandible, both sides	4: 2 each side (2ndPM, 3rdPM)	0 (simultanious extraction and implant installation)	3 animal groups (A), (B), (C): 5 animals in each	Screw shaped, Beijing Leidon Biomaterial limited (similar to Straumann SP)	3,5x12	Ti	NA	Tissue level	4: 2 on each side	1-stage	NA	4m and 2w.	Yes, dogs randomly divided into groups	NA (No susprastructure used)	NA	2 weeks after implantation	4m and 2w	Brush daily

97. Madi et al	2013	Dog, Beagle	Experimental PI at different implant surfaces	6	Mandible	PM1 PM2 PM3 PM4 On both sides of the mandible	3m	Interquadrant, anterior-posterior randomized fashion	3 screw shaped and 1 cylinrical - the (4) HA plasma-sprayed+pressurized	3,3x10 mm (screw shaped) and 3,25x10 mm (cylindrical)	Ti	(1) Machined (2) Sandblasted, acid-etched (3) Sputter HA-coat (4) HA plasma-sprayed+pressurized	Bone level	8: 4 on each side of the mandible	1-stage	NA	0+3m	YES, implants placed in a randomized order	No	Metronidazole 11mg/kg postoperatively for three days	(A) 2w after implant placement	(A) 3m	(A) 2% CHX rinse 3t/w+scaling 1t/m
98. Charalampakis et al	2014	Dog, Labrador (same animals as study 12 in this table)	Teeth vs implants/ 2 implant surfaces	6 according to material and method, 5 according to absctract.	Mandible, implants on the right side, teeth on the left	Right mandibular premolars and first molar. Three right anterior maxillary premolars	3m	Split mouth (CHX vs SALINE) Inter-quadrant (4 surfaces)	Screw shaped Nobel Biocare MKIII, NP	3,3x10	Ti	A: Turned B: TiUnite	Bone level	4 (On the right side of the mandible)	1-stage	NA	3+0	Yes- Pairwise placement	No	NA	2w after implant installation	3m minus 2w	Tooth and abutment cleaning 3 times a week
99. Guo et al	2014	Dog, Beagle	TREATMENT – DNA vaccines for preventing peri-implantitis and retarding bone loss	16	Mandible, both sides	4: 2 on each side (PM2, PM3)	0 – immediate implantation	Animals divided in 4 groups: (A) pVAX1-kpg, (B) pVAX1-rgpA, (C) pVAX1-rgpB, (D) pVAX1	Screw-shape	3,5x10	Ti	NA	Tissue level	4: 2 on each side	1-stage	NA	3,5m+0	NA	NA (no suprastructure)	NA	NA	NA	NA
100. Madi et	2014	Dog, Beagle	Experimental PI at different implant surfaces	4	Mandible	PM1 PM2 PM3 PM4 On both sides of the mandible	3m	Interquadrant, anterior-posterior randomized fashion	3 screw shaped and 1 cylinrical - the (4) HA plasma-sprayed+pressurized	3,3x10 mm (screw shaped) and 3,25x10 mm (cylindrical)	Ti	(1) Machined (2) Sandblasted, acid-etched (3) Sputter HA-coat (4) HA plasma-sprayed+pressurized	Bone level	8: 4 on each side of the mandible	1-stage	NA	0+3m	YES, implants placed in a randomized order	No	Metronidazole 11mg/kg postoperatively for three days	(A) 2w after implant placement	(A) 3m	(A) 2% CHX rinse 3t/w+scaling 1t/m
101. Martinez et al	2014	Dog, Beagle	Soda lime glass coated abutments with silver Nano-particles	5	Mandible	PM1 PM2 PM3 PM4 M1 On both sides of the mandible	12w	Inter-qudrant: Mesial implant as control, 2 distal ones as test	Screw shaped (Phibo dental solutions, Barcelona, Spain)	3,75x11,5	Ti	NA	Bone level	6: 3 on each side of the mandible	2-stage	NA	2m+4w	No	No	No	(A) After abutment connection	(A) 4w	(A) brush and dentifrice 5d/w
102. Pârvu et al.	2014	Dogs	Placebo and Ibuprofen	6	Maxilla	1 (2ºPM)	0	-	Screw-type (OT medical GmbH, Bremen, Germany)	2.0x8.0	Ti	NA	Tissuelevel	1	1 stage (however, with 5w healing)	-	5w + 0	Yes	-	-	-	-	-

103. Battula et al	2015	Dog, Hound	2 implants (A, B) with/ without ligature	8	Mandible	8 (Premolars 3+4 and molars 1+2)	0 (immediate post extraktion placement in each respective socket)	Animals divided in 2 groups: Experimental peri-implantitis vs. Control. 4 of each implant in all animals	A: Screw shaped – Tapered Screw-Vent MTX, Zimmer Dental B: Screw shaped cervically and apically, central porous structure.	4,1x13 mm	A: Ti B: Ti with central Tantalum portion	-	Bone level.	8	2-stage (Allograft bone material and collagen membrane used for all implants)	NA	3m + 0	Yes	No (Not specifically stated, no extraktion of antagonizing teeth reported)	NA	None (ligatures were placed during the abutment surgery in the experimental group).	-	-
104. Carcuac et al	2015	Dog, labrador	TREATMENT STUDY 4 implants/ surfaces. CHX vs Saline	6	Mandible, both sides	All mandibular premolars. 1st, 2nd and 3rd maxillary premolars	3m	Split mouth (CHX vs SALINE) Inter-quadrant (4 surfaces)	Screw shaped. A, B, and C: Astra Tech Implant system. D: Nobel Biocare.	A,B,C: 3,5x11 D: 3,3x11,5	Ti	A: TiOblast B: Osseospeed C: AT-I D: TiUnite	Bone level	8 (4 on each side of the mandible)	1-stage	NA	3+0	Yes – identical placementsequence on both sides but randomized sequence between dogs	No	NA	2w after implant installation	3m minus 2w	Plaque control 3 times a week
105. Fickl et al	2015	Dog, Beagle	Experimental peri-implantitis around 3 different implants	5	Mandible, both sides	8: 4 each side (1stM, 2ndPM, 3rdPM, 4thPM)	8w	Inter-quadrant	Screw-type: (B) BIOMET 3i T3 (N) Nobel Replace Tapered (S) Straumann bone level	(B) 3,25x8,5 (N) 3,5x8) (S) 3,3x8)	Ti	(B) OsseoTite (N) TiUnite (S) SLActive (from manufacturers info, not specified in article)	Bone level, although placed in a 2 mm epi-crestal position	10: 5 on each side	1-stage	NA	0+2m	Yes, implants randomly inserted	NA (No suprastructure used)	For 1w after surgery: Amoxicillin 500mg x2 IV	(1) After implantation (2) After ligature removal	(1) 2m (2) 8w	(1) 0,12% CHX rinse (interval?) (2) Daily brush with pumice+0, 12% CHX followed by 0,12% CHX spray
106. Huang et al	2015	Dog, beagle	Crestal vs subcrestal position. Screwed in vs tapped implant-abutment-interface	6	Mandible	8: M1, PM4, PM3, PM2 on one side of the mandible	8w	Ligatures around all implants	Screw-shaped	(A) Astra teck OsseoSpeed 3,5x8 mm (B) Bicon Dental Implants, Integra-CP 3,5x8 mm	TI	(A) Fluoride-modified TiO2 grit blasted surfaces (OsseoSpeed) (B) Plasma-sprayed calcium-phosphate	(A) 1 crestal and 1 subcrestal (1,5 mm) in each animal (B) 1 crestal and 1 subcrestal (1,5 mm) in each animal	4 on one side of each mandible	2-stage	10 mm	12w+4w	NA	No	Penicillin G 40.000IU/kg intramuscularly once/ day in 1 week	(A) 1 week before implant surgery (B) After abutment surgery (C) from 10d post abutment surgery to ligature placement	(A) at one occasion (B) 10d (C) Approx 18d	(A) Scaling to remove supragingival calculus (B) CHX rinse every second day 0,12% (C) Brush with 0,2% chx gel every other day
107. Ikumi et al	2015	Dog, Beagle	Overload – various magnitudes of static compressive stress (0-180 MPa)	2	Tibia	-	2m	Compressive stress in different magnitudes	Screw shaped	4,0x? custom made implant	Ti	NA	Bone Level	8: 4 in each tibia	2-stage	NA	2m+2m	NA	Yes	Single dose ampicillin 50mg/kg after implant surgery	-	-	-

108. López-Piriz et al	2015	Dog, Beagle	3 different antimicrobial glassy coated abutments effect on bacteria, biofilm and bone loss	5	Mandible	All Ms and PMs on both sides of the mandible	3m	Inter-qudrant: (1)control (2) ZnO-glassy (3) G3 glassy coating (4) n-Ag coating	Screw shaped SEVEN, MIS	NA	Ti	?	Bone level	8; 4 on each side of the mandible	2-stage	NA	8w+	No	NA (No suprastructure)	NA	(A) After abutment connection	(A)4w	(A) Toothbrush and dentifrice 5d/w
109. Namgoong et al.	2015	Beagle dogs	3 surfaces: U, SA, SA/HA	5	Mandible	10: 5 each side (1ºPM, 2ºPM, 3ºPM, 4ºPM, 1ºM)	12w	Split-mouth	Screw-type (Osstem, Busan, Korea)	3.0x8.5	Ti	U, SA, SA/HA	Bonelevel (1 mm subcrestally)	3	2 stage (submerged)	NA	12w + 2w	Yes	No	NP	During implant healing: toothbrush 2x/w for 12w. Post-op.: NP	12w pre-op.	toothbrush
110. Park et al.	2015a	Beagle dogs	Different plaque control methods	6	Mandible	10: 5 each side (1ºPM, 2ºPM, 3ºPM, 4ºPM, 1ºM)	3m	Split-mouth	Screw-type (GS II RBM fixture; Osstem, Seoul, Republic of Korea)	3.5x8.5	Ti	NA	Tissuelevel	4	2 stage	NA	3m + 0	No	No	After GBR (2d)	1m after ligature removal	Only once	3 groups: (G1) manual irrigation-syringe; (G2) dental water jet; (G3) dental water jet + flossing
111. Park et al.	2015b	Beagle dogs	Different GBR methods	6	Mandible	10: 5 each side (1ºPM, 2ºPM, 3ºPM, 4ºPM, 1ºM)	3m	Split-mouth	Screw-type (TSIII SA fixture; Osstem, Seoul, Republic of Korea)	3.5x8.5	Ti	SB/AE	Tissuelevel	4	2 stage	NA	3m + 0	No	No	No	1m after ligature removal	Only once	Dental water jet + flossing
112. Pirih et al	2015	Mice, C57BL/6 J male	Ligature (10 mice) vs control (8 mice)	18	Maxilla	3: M1, M2, M3 on the left side	8w	Comparison between animals	Screw type (G. Hartzell and Son, Concord, CA, USA)	0,5x1	Ti	U	Tissuelevel	1	1 stage	-	4w+0	Yes, toss of a coin	No	For 4w aft tooth extractions/ implant surgery respectively. Diluted in drinking water	No	No	No
113. Pirih et al	2015	Mouse, C57BL/6 J	Local P. gingivalis lipopolysaccharide injection vs No injection and vehicle injection		Maxilla	M1, M2, M3 on the left side of the maxilla	8w		Screw type, custom made.	0,5 x 1 mm	Ti	U	Bonelevel	1	1 stage	-	4w+0	No	-	Sulfamethoxazole and trimethoprim oral suspension 850 micro g/ 170 micro g/mL in drinking	-	-	-

																				water for 4w after extraction and 4w after implant surgery			
114. Shi et al	2015	Dog, Beagle	TREATMENT STUDY non-equilibrium plasma vs conventional treatment	6	Mandible	PM4 on both sides of the mandible		Split mouth (treatment)	Screw-shape (Anthofit, OIIM, 35100, Anthogyr)	3,5 x 10	Ti	A BCP sandblasted surface consisting of a mixture of hydroxyapatite and beta-tricalcium phosphate with Ra ranging from 1,5 to 2,0 micro-m.	Bone level	2: 1 on each side	1-stage	-	0+3m	Yes, according to treatment	No	NA	(a) From start of study	(A) NA	(A) Toothbrush and sahline every 3rd day
115. Takamori et al	2015	Rat, Lewis	Onset of peri-implantitis vs periodontitis	25	Maxilla	Maxillary right M1	0 – implant placed directly	Comparison between animals	Screw-form (Sky Blue, Fukuoka, Japan)	2x 4,5 mm	Ti-6AL-4V	NA	Tissue level	1: 1 on the right side	1-stage	-	0+	No	NA	No	None	None	None
116. Htet et al	2016	Dog, Beagle	TREATMENT Er:YAG VS Photodynamic therapy and titanium bur with/ without citric acid	5	Mandible	All mandibular PMs	6w	Ligatures around all implants	Screw shaped	3,3x10 mm Brånemark	Ti	Ti-unite	Bone level	6: 3 in each side of the mandible	1-stage	NA	8w	Yes – acoording to treatment	NA (no suprastructure)	NA	(A) After implant surgery (B) After ligature removal	(A) 8w (B) 3w	(A)+(B) CHX 0,12% 3 times a week. Scaling once a month
117. Ishii et al	2016	Dog, Beagle	UV-light irradiated SLA-surface vs conventional SLA-surface	3	Mandible	PM2, PM3, PM4 on both sides	0m immediate post extraction implantation	Split mouth	Screw shaped	3,3x8 mm Straumann SP	Ti	SLA (sandblasted + AE)	Bone level	4: 2 on each side of the mandible	1-stage	NA	90d+0d	No	No	-	(A) After implant surgery	(A) 90d	(A) 0,12% CHX
118. Machtei et al	2016	Dog, American foxhound	TREATMENT STUDY	5	Mandible	All PMs on both sides of the mandible	12w	Ligatures around all implants	Screw shaped Seven, MIS	3,75x10 mm		Sand blasted, acid etched	Bone level	6: 3 on each side of the mandible	2-stage	NA	2m+0	No	No	No	(A) after implant installation	(A) 2m – until abutment connection	(A) 0,2% CHX rinse daily
119. Madi et al	2016	Dog, beagle	Experimental PI at different implant surfaces	4 (3 according to number, but 4 according to the amount of implants 8 per animal total 32)	Mandible	PM1 PM2 PM3 PM4 On both sides of the mandible	3m	Inter-quadrant, randomized placement	Screw shaped	3,3x13 mm	Ti	(1) Machined (2) Sandblasted, acid-etched (3) Sputter HA-coat (4) HA plasma-sprayed+pressurized	Bone level	8: 4 on each side of the mandible	1-stage	NA	0+3m	YES, implants placed in a randomized order	No	Metronidazole 11mg/kg postoperatively for three days	(A) 2w after implant placement	(A) 3m	(A) 2% CHX rinse 3t/ w
120. Nguyen Vo et al.	2016	Mice	Different follow-up times	60	Maxilla	1 (1ºM right side)	8w	One quadrant	Screw-type	0.8x1.5	Ti	U	Tissuelevel (bonelevel?)	1	1 stage	-	4w + 0	No	No	NP	-	-	-
121. Xu et al	2016	Dog, Beagle	TREATMENT STUDY – bone fill with addition of	6	Mandible	PM1, PM2, PM3, PM4	3m	Interquadrant and split mouth	Screw type	3,8 x 10 mm	Ti	NA	Bone level	4: 2 on each side	2-stage	NA	2m+1m	No	NA- no suprastructure	During treatment	NA	NA	NA

			stem cells and BMP-2 in 4 different groups																				
122. Godoy-Gallardo et al	2016	Dog, Beagle	Implants with 3 surfaces: Ti, Ti_Ag Ti-TSP	5	Mandible	All PMs on both sides of the mandible	3m	Inter-quadrant	Screw-type (Soadco S.A., Escaldes-Engordany, Andorra)	3,5x8 mm	Ti	Ti-group: Sandblasted and acid etched Ti-Ag: same + silverelectrodepositi on Ti-Tsp: same + TESPA sinlanisation	Tissue level	6: 3 on each side	1-stage	Min 3 mm between implants	2m+0	Yes, random implant placement	NA-no suprastruct ure	Amoxicillin post op.	(A) After implant insertion. (B) 10d after implant insertion	(A) 10d (B) 2m	(A) cleanin with CHX on gauze. (B) Brusch with CHX 3t/ w.
123. Park et al.	2017	Beagle dogs	Immediate vs. delayed implantation	4	Mandible	6: 3 each side (3ºPM, 4ºPM, 1ºM)	0 (3ºPM) and 3m (4ºPM)	Split-mouth	Screw-type (TSIII SA fixture; Osstem, Seoul, Republic of Korea)	3.5x8.5	Ti	SB/AE	Tissuelevel	4	1 stage (3ºPM) 2 stage (4ºPM)	NA	0 + 0 (3ºPM) 3m + 0 (4ºPM)	No	No	After GBR (3d)	2w after ligature removal	Only once	Dental water jet + flossing
124. Lin et al	2017	Dog, Beagle	Stainless steel ligature investigated	6	Mandible	All PMs on both sides of the mandible	4w	Ligatures around all implants	Screw-type (Straumann tissue levele)	3,3x8 mm	Ti	Sand blasted and acid etched (SLA)	Tissue level	6: 3 on each side, tot 36	1-stage	NA	12w+0	No	NA-no suprastruct ure used	30 min before all surgical treatment	(A) after abutment connection	(A) 12w	(A) brusch 2t/w
125. Koutouz is et al	2017	Rat, Wistar	Polymicrobial inocula by gingival lavage vs sham	12 (5 were analyzed)	Maxilla	M1, on both sides	1m	Comparison between animals	Screw-type custom made	1,5x2	Ti	Turned	Bone level	2: one on each side of the maxilla	2-stage	-	2m+1w	No	No	Yes, before for 4 consecutive days prior to inoculation	(A) Prior to inoculation	(A) 4d	(A) CHX swabbing
126. Moest et al	2017	Dog, Beagle	TREATMEN T STUDY Influenze of abutment material on tissue regeneration after PI treatment	8	Maxilla+M andible	All PM2, PM3, PM4	8w	Inter-quadrant	Screw-type (Straumann Bone Level Roxolid)	3,3x10	Ti-alloy (Roxolid)	SLActive	Bone level	51 in total in 8 animals	1-stage	Min. 10 mm center to center	2m+0	Yes, according to abutment type after surgical treatment	No	No	(A) After implant insertion	(A) 2m	(A) Brushing of teeth and implants
127. Ramos et al	2017	Dog, Beagle	TREATMEN T STUDY	8	Mandible	PM1, PM2, PM3, PM4, M1 on both sides of the mandible	8w	Inter-quadrant	Screw-typ (Straumann Bone Level)	3,3x10	Ti	SLA	Bone level	8: 4 on each side	1-stage	NA	2m+0	Yes, according to treatment	No	Yes, for 3d after implant insertion	(A) 4w after extraction (B) After implant insertion	(A) 4w (B) 8w	(A) Dental prophylax is (B) Dental prophylax is every 4w
128. Rodrigu ez et al	2017	Mini-pig	Influence of microtextured implant surfaces on PI-induction (5 animals) on PI-treatment (3 animals)	6	Mandible	PM2, PM3, PM4	12w	Split-mouth	Screw-type (BioHorizons, tapered internal system)	3,4x9 mm	Ti	(RBT) Resorbable blast textured And (LM) Laser-microtextured	Bone level	6: 3 on each side	1-stage	PI group (3 animals) 3,451 ± 1,03 mm Treatment group (3 animals): 3,385 ± 1,445 mm	12w+0	Yes, according to implant type	No	No	NA	NA	NA

129. Wong et al	2017	Mouse	Spontaneous healing potential after ligature removal at implants and teeth	35	Maxilla	Left maxilla molars	8w	Split mouth – implant on left side and tooth on right side and comparison between animals	Screw-type (DP Machining Inc, La Verne, Calif)	0,5x1	Ti	Turned	Tissue level	1: 1 on the left side	1-stage	-	4w+0	Yes	No	NA	NA	NA	NA
130. Yu et al	2017	Mouse	Experimental periodontitis and peri-implantitis in toll-like receptor 2 vs wild type mice	62	Maxilla	Left maxillary molars	6w	Split mouth – implant on the left side and tooth on the right side	Screw-type (DP Machining Inc, La Verne, Calif)	0,5x1 mm	Ti	Turned	Tissue level	1: 1 on the left side	1-stage	-	4w+0	No	No	Yes – for 2w after tooth extractions and 4w after implant insertion	NA	NA	NA
131. Hiyari et al	2018	Mouse	Ligature induced PI in different mouse strains	65	Maxilla	Left maxillary molars	8w	Comparison of 3 mouse strains (1) C57BL/6J (n=22) (2) C3H/HeJ (n=22) (3) A/J (n=21)	Screw-type (DP Machining Inc, La Verne, Calif)	0,5x1	Ti	Turned	Tissue level	1: 1 on the left side	1-stage	-	4w+0	Yes, to ligature or control (no ligature)	No	For 4w aft tooth extractions/ implant surgery respectively. Diluted in drinking water	NA	NA	NA
132. Hiyari et al	2018	Mouse	Progression of ligature induced periodontitis vs peri-implantitis	85	Maxilla	Left maxillary molars (M1, M2, M3)	8w	Comparison between animals + split mouth with implant on one side and tooth on the other	Screw-type (DP Machining Inc, La Verne, Calif)	0,5x1	Ti	Turned	Tissue level	1: 1 on the left side	1-stage	-	4w+0	Yes, to ligature or control (no ligature)	No	For 4w aft tooth extractions/ implant surgery respectively. Diluted in drinking water	NA	NA	NA
133. Huang et al	2018	Dog, Beagle	Bone defect configuration depending on implant placement depth and implant type	6	Mandible	PM2, PM3, PM4, M1 on both sides of the mandible	8w	Interquadrant: 2 of each implant type. 1 of each implant type placed 1,5 mm sub-crestally and the other 2 at bone level	Screw-type (OsseoSpeed, (A) Astra, Mölndal, Sweden and (B) Integra-CP. Bicon, Boston, USA I	(A)&(B) 3,5x8	Ti	(A) Fluoride modified TiOblast (B) Plasma-sprayed calcium-phosphate	Bone level and 1,5 mm sub-crestal level	4 on the left side, total of 24 implant in the study	2-stage	NA	12w+4w	Yes	No	For 7d after extractions.	(1) After tooth extraction (2) After implant insertion (3) 10d after abutment surgery	(1) 1w (2) 1w (3) 4w minus 10d	(1) & (2) Cleaning with 0,12% CHX solution (3) 0,12% CHX irrigation every 2nd day initially, then brush every 2nd day

Ti = titanium; TPS = titanium plasma sprayed; HA = hydroxyapatite, U = ~~uncoated~~ turned/machined, AE – acid-etched, Before PI = prior to induction of experimental peri implantitis; After PI = following induction of experimental peri-implantitis; NA = Not available; CHX = Chlorhexidine

Table S2. Induction and outcome of experimentally induced peri-implant bone loss in studies included in the qualitative synthesis.

Induction of peri-implant bone loss								Diagnostic markers								Peri-implant bone defect					
Author	Published	Method (ligature/ overload/other)	Ligature material	Ligature size	Ligature exchange	Duration	Control side protocol	Clinical measurements (BoP/ PPD etc)	Mobility	Microbiological sampling (cultivation/ PCR)	X-ray	Histometric measurements	Histologic evaluation	Other	Registratio n (days from baseline/ ligature placement)	Vertical Bone loss Mean ± SD (mm)	Horizontal Bone loss Mean ± SD (mm)	Measuring method	Development after ligature removal (progression/ healing)	Bone loss ± SD (mm) at control side	Lost implants
1. Klinge	1990	Ligature	Silk	NA	NA	5m	Ligatures around correspon ding PMs on the contralater al side	-	-	-	Periapical radiographs monthly	Marginal bone breakdown measured	Ground sections	-	150	Implants: 1 mm on average<br>Teeth 5 mm on average	NA	Periapical radiographs	-	-	No losses reported
2. Hickey et al	1991	Ligature (submarginal, wrapped around abutment 15 times)	Silk	4-0	No	45d	Scaling and cleaning every 5d.	CAL, PPD, GI, PI,	-	Subgingival samples/ cultivation from all implants. At day 0, 14 and 45.	Periapical x-rays	-	-	-	45	NA, only CAL was measured	-	-	-	Control side: 0,08 mm	-
3. Akagawa et al	1992	Ligature (1 mm above gingival margin)	Silk floss	NA	No	12m (monthly clinical examinati ons)	Brush, interdental brush, electric pencil brush. Interval NA	Pi, Gingival index according to Löe & Silness -63, PPD at 25g pressure	-	-	-	-	-	-	365	-	-	-	-	-	None reported
4. Ericsson et al	1992	Spontanious plaqueaccumulalti on	-	-	-	90d	-	Gross examination of plaque and inflammation	“non mobile” at end of experimen t. Method NA	-	-	Decalcified blocks sectioned for light microscopy. Size and composition of ICT etc.	Composition of plaque associated ICT and abutment ICT tissues and cells. Some differences teeth and implants	-	90	Measurements in pocket depth, not in bone loss	-	-	-	-	-
5. Leonhard t et al	1992	Ligature, subgingival around 2 implants and 2 teeth	Cotton	NA	No	42d	None	-	-	At baseline, atd21 plaque accumulation and 30d after ligature removal	-	Middle implant reported in Lindhe 1992	-	-	42	-	-	-	-	-	-
6. Lindhe et al	1992	Ligature, submarginal Followed by 1 month plaque accumulation after removal	Silk	NA	Replaced after 3w	6w	Ligatures around 2 teeth	PI, BoP,	All teeth and implantscl inically stable at end of study	In Leonhardt et al, 1992	Periapical radiographs	Size and content of ICT reported in Morphometric measurements	Size and content of ICT reported in Morphometric measurements	-	32 with ligature + 30 with spontaneou s accumulati on	Implant 3,2±0,3 Tooth 1,1±0,6	-	X-rays, periapical with eggen holder. Not specified whehter a mean- or max value was used for each implant	-	-	None
7. Akagawa et al	1993	Ligature (1 mm above gingival margin)	Silk	NA	No	12m (Bacterial sampling	Brush, interdental brush,	-	-	Types and number of bacteria classified	-	-	-	-	365	-	-	-	-	-	None reported

						at baseline and 1, 3, 6, 9, and 12 months after ligature placement)	electric pencil brush. Interval NA			according to morphology by phase contrast microscopy. No difference between implants and teeth. Motile rods and spirchetes significantly increased between 1 and 6 monts after ligature placement											
8. Grunder et al	1993	Ligature	Cotton floss	NA	Yes every 1m	5m	Ligatures around all implants	PI, GI, PPD, CAL	Periodontometer of Muhlemann, 500g force	-	Periapical, long cone, monthly	Ground sections. New bone after surgery measured	-	-	150	-	-	-	-	-	-
9. Jovanovic et al	1993	Ligature placed around the coronal 3-4 mm of the implant and placed in contact with pre-made bony defect.	Silk	NA	-	12w	7 control implants left without ligature	Defects measured during peri implantitis-surgery	-	-	-	Ground sections	-	-	84	NA since defects were created at implant surgery	Increase in horizoantal defect in mm: Brånemark 0,75 IMZ 0,96 Integral 0,91	Clinical measurement during peri-implantitis surgery	-	-	1 Brånemark implant lost during ligature phase
10. Lang et al	1993	(A) Ligature implant (B) Spontaneous plaque accumulation implant (C) Natural tooth (M3)	Silk	NA	New ligatures on top of old ones at 3m and 6m	8m	None	PI, GI, CAL	-	-	Periapical radiographs	Supposed to have benn reported in subsequent study that couldn't be retrieved.	Supposed to have benn reported in subsequent study that couldn't be retrieved.	-	240 (baseline at ligature placement after 30d plaque accumulation)	Radiographic loss (240 days) (A) 1,01 ±0,74 (B) 0,36 ±0,31 (C) 0,64 ±0,67 Data from 30, 60, 150, 180 days also available in table 2 on page 8	-	Periapical x-rays, standardized with oral bite block. Mean of mesial and distal values.	-	Registred in vertical bone loss box	None reported
11. Schou et al	1993	Ligature – subgingival and supragingival with lingual knot	Silk, braided (Ethicon)	3-0	no	7w	(1) Implant +- ligature (2) Ankylosed tooth +- ligature (3) normal tooth +- ligature	BoP, PPD, CAL. Electronic probe 25g, 0,5 mm diam.	-	-	-	-	Size and cell composition of ICT	-	49	-	-	-	-	-	1 implant and 2 replanted teeth planned to remain ligated.
12. Schou et al	1993	Ligature – subgingival and supragingival with lingual knot	Silk, braided (Ethicon)	3-0	no	7w	(1) Implant +- ligature (2) Ankylosed tooth +- ligature (3) normal tooth +- ligature	BoP, PPD, CAL. Electronic probe 25g, 0,5 mm diam.	-	-	IO long cone, 2, 4, 6 and 7w.	-	-	-	49	Diagrams on bone loss and attachment loss in the paper. Significant bone loss around ligated implant and ankylosed teeth.	-	X-rays, probing	-	-	1 implant and 2 replanted teeth planned to remain ligated.

13. Singh et al	1993	Ligature	Silk	NA	NA	NA	Ligatures around all implants	Clinical evalutation of defect before and after treatment	-	-	-	Ground sections SEM	-	-	NA	CTRL: 3,75±0,46 TEST 1: 2,94±0,49 TEST 2: 3,00±0,92	-	-	-	-	-
14. Lang et al	1994	(A) Ligature (B) Spontaneous plaque accumulation (C) Control	Silk	NA	New ligatures on top of old every 4w	(A) 4m (B) 2m (C) 2m	Rubber cup and pumice once a week + Soft brush 3 times/ w.	PI, GI, BoP, PPD with Florida proble set at 0,2N, CAL	-	-	-	Ground sections with fixed probe tip on mesial and distal sides: histologic probing depth, alveolar bone to probe tip distance, histologic attachment level	-	-	60, 240	NA, only CAL was measured	-	-	-	Registered in vertical bone loss box	Proble tip closer to bone crest in ligature group, similar in the 2 other groups
15. Schüpbach et al	1994	Ligature	Cotton floss	NA	Yes every 1m	5m	Ligatures around all implants	PI, GI, PPD, CAL	Periodontometer of Muhlemann, 500g force	-	Periapical, long cone, monthly	Ground sections. New bone after surgery measured	-	-	150	NA, 30-50% bone loss, not further specified	-	-	-	-	-
16. Weber et al	1994	Ligature, submarginal	Silk	4-0	No	60d	Ligatures around all implants, one dog received NSAID treatment and the other Placebo	Increasing GI and probing depths during ligature phase<br>TC-Sn-MDP given IV, uptakte then measured with a radiation probe	All implants firmly anchored at start of ligature phase	-	IO at postsurgery, after 3m healing and after 60d ligature phase.	-	-	-	60	NSAID (bone loss %/ m) 0,866±0,351%<br>Placebo 5.729±0,384%	-	X-ray, periapical. Stents fabricated for each site	-	-	All implants integrated at baseline (after 3m healing)
17. Cook et al	1995	Ligature (tightened between fixture and abutment, suture ends extended to oral cavity)	Silk	4-0	No	4w, 8w, 16w and 26w	Weekly brushing. No ligature	-	-	-	Routine dental radiographs	Ground sections - Bone and tissue apposition, porosity of coating on experimental implants, HA thickness on contral implants	Degree of inflammatory response (0-5): Minimal in both CSTi and HA at all time periods examined.	-	28+56+112+182	Histology results at 182d (5 animals): 14x CSTi: 1,96±0,94 28x HA: 2,69±1,27	-	Histo. Mean + SD	-		2 lost: One prior to abutment surgery, the other removed at 2nd surgery due to infection
18. Ericsson et al	1995	Spontanious plaque accumulation around the most distal implant on both sides	-	-	-	9m	Daily brushing and dentifrice around the 2 medial implants on each side	Gross examination of plaque and inflammation	-	-	-	Decalcified blocks sectioned prepared for light microscopy according to method described by Berglundh in 1994 (EPON embedded sections). Size and composition of ICT etc.	Composition of plaque associated ICT and abutment ICT tissues and cells. No significant differences between groups	-	270	Measurements in pocket depth, not in bone loss	-	Clinical	-	-	-
19. Hürzeler et al	1995	Ligature	Silk	4-0	Additional ligatures over old ones every 2w	3m	Ligatures around all implants	-	-	-	-	-	-	-	90	30-50% bone loss. No further deails provided.	-	-	-	-	-

20. Marinello et al	1995	Ligature, submarginal position	Cotton	NA	No	4-6w (at approx. 25% destruction of original bone height) + 1m (2 dogs) and 3 m (3 dogs)spontaneous plaque accumulation	Ligatures around all implants	-	-	-	-	Various measures of mucosa and plaque associated infiltrate	Various measures of mucosa and cell+tissue composition of plaque associated infiltrate.	-	150+90	NA – At 3m from ligature removal, scar tissue healing without evidence of ongoing tissue destruction was noticed compared to the 1m group.	-	-	-	-	-
21. Warrer et al	1995	Ligature – supramarginal position without attempt to place them submarginally	Cotton-wool	NA	Every 3m with the same supramarginal technique	9m	4 groups:<br>1-2: Keratinized mucosa with- (1) and without ligature (2)<br>3-4: Non-keratinized mucosa with- (3) and without ligature (4)	PI, BoP, PPD, CAL and recession on 4 surfaces per implant (later pooled) every 3m<br><br>Data in table 1 of paper	-	-	-	Ground sections	Remaining bone height on mesial and distal sides	-	270	Bone loss calculated from fig 7:<br><br>(1) 0,96 mm<br>(2) 0,8 mm<br>(3) 1,65 mm<br>(4) 1,05 mm<br><br>Bone-implant contact leangth in % of implant leangth (table 1):<br><br>(1) 63±7,5<br>(2) 65±9,01<br>(3) 54,38<br>(4) 61,88		Histo - mean of mesial and distal aspects of implants			All implants integrated
22. Ericsson et al	1996	Ligature (submarginal)	Cotton (floss)	NA	No	6-8w (about 20% bone loss) + 1m spontanoius plaqueaccumulation followe d by amoxicillin for 3w	Ligatures around all implants	-	-	-	Before ligature and at 6-8w	Ground sections	-	-	5m + 1w+10d atfter ligature removal. (3w amoxicillin starting 1m after ligature removal)	No specific measurement at ligature removal	-	X-ray	-	-	-
23. Isidor	1996	(1) Overload on one side, (2) Ligature on other side and symphysial region (marginal position)	Cotton cord	NA	-	18m	(1) Brush once/ week. Debridement once/ month. (2)	BoP, PPD, CAL	Periotestand manual mobility every 3m	-	IO. Parallelling technique	Ground sections<br>Marginal bone loss<br>BIC	-	-	540	(1) Periotest: 2 implants removed due to mobility. 6 remaining with values 6, 18, 45, 25, -7, -7. Almost complete loss of integration in all but 2	-	Light microscopic analysis of ground sections	-	-	2 implants removed at abutment surgery<br>2 lost during overload phase

							No hygiene									implants with 1,8 and 1,9 mm bone loss resp. CAL loss: 0,6 mm (2) Periotest: -6, -5, -6, -7, -2, -5, -5, -4, -6, -5 Marginal bone loss (mm): 2,4 (0,8-4) CAL loss: 1,1 mm					
24. Persson et al	1996	Ligature, submarginal	Cotton	NA	-	About 6w/ 20% radiographic bone loss	Ligatures on both sides	Bone loss measured with probe during PI-surgery.	-	-	IO	Marginal bone loss after GBR.	Cell composition and localisation of ICT	-	42	Implant shoulder to bottom of defect: 1,8±0,45 (30 days after ligature removal)	-	Periodontal probe, mean value of 4 sides per implant	-	-	-
25. Schou et al	1996	Ligature – subgingival and supragingival with lingual knot	Silk, braided (Ethicon)	3-0	no	7w	(1) Implant +- ligature (2) Ankylosed tooth +- ligature (3) normal tooth +- ligature	BoP, PPD, CAL. Electronic probe 25g, 0,5 mm diam.	-	Phase contrast microscopy for presence and amount of various bacteria	-	-	-	-	49	-	-	-	-	-	1 implant and 2 replanted teeth planned to remain ligated.
26. Fritz et al	1997	Ligature	Silk (braided)	NA	No	6m	Ligatures around all implants, contralateral 2nd molar and non-ligated second premolar (anterior abutment)	Plaque score, redness, PPD	NA	-	Periapical with "quantitative subtraction method"	-	-	-	90+180	Root form implants: 90d: 1,35±0,24 180d: 1,72±0,28 Plate form: 90d: 1,59±0,44 180d: 1,89±0,45	-	"X-ray periapical with standardized technique Quantitative subtraction method"	-	-	-
27. Hanisch et al	1997	Ligature (submarginal)	Cotton	NA	Yes, every 1m	10m + 1m additional plaque accumulation	Ligatures around all implants	Modified PI, GI, BOP, PPD, CAL. Calibrated probe	Periotest, Siemens	Submucosal samples from 1 randomly chosen implant in maxilla and mandible respectively. Morphotype, culture and DNA-probe	-	-	-	-	300	Maxilla: 3,3±1,2 Mandible: 3,4±1,4 Overall: 3,3±1,3 (2 months after ligature removal)	Maxilla: 2,0±0,5 Mandible: 2,0±0,4 Overall: 2,0±0,5	Clinical measurement at abutment connection and at reconstructive surgery after removal of soft tissue	-	-	1
28. Hürzeler et al	1997	Ligature	Silk	4-0	Additional ligatures over old ones every 2w	3m	Ligatures around all implants	--	-	-	-	-	-	-	90	-	-	-	-	-	-
29. Isidor	1998	(1) Overload on one side, (2) Ligature on other side and	Cotton cord	NA	-	18m	(1) Brush once/ week. Debridem	-	Periotest and manual mobility every 3m	-	IO. Parallelling technique	Ground sections Marginal bone loss BIC	-	-	540	Fig. 2 in article X-rays (540 d) Overload: 4,6 Ligature: : 1,8	-	"X-rays Periapical with	-	-	2 implants removed at abutment surgery

		symphysial region (marginal position)					ent once/ month. (2) No hygiene									Histology (540 d) Overload: 4,8 Ligature: 2,4		parallelling technique Not specified whehter a mean- or max value was used for each implant Histo. Mean of 2 mid-implant sections from the buccal aspect of each implant "			2 lost during overload phase
30. Isidor	1997	(1) Overload on one side, (2) Ligature on other side and symphysial region (marginal position)	Cotton cord	NA	-	18m	(1) Brush once/ week. Debridem ent once/ month. (2) No hygiene	-	Periotesta nd manual mobility every 3m	-	IO. Parallelling technique	Ground sections Marginal bone loss BIC	-	-	540	(1) Periotest: 2 implants removed due to mobility. 6 remaining with values 6, 18, 45, 25, -7, -7. Almost complete bone loss in all but 2 implants with 1,8 and 1,9 mm bone loss resp. (2) Periotest: -6, -5, -6, -7, -2, -5, -5, -4, -6, -5 Marginal bone loss (mm): 2,4 (0,8-4)	-	Light microscopic analysis of ground sections	-	-	2 implants removed at abutment surgery 2 lost during overload phase
31. Saito et al	1996	Spontaneous plaque accumulation around functionally loaded implants. Soft food.	None	-	-	180d	2 different implant types vs control teeth, plaque accumulat ion around all implants	At baseline, 90d and 180d: PI, PPD, BoP,	At baseline 90d and 180d: Periotest	Yes, baseline, 90d and 180d: detection of black pigmented rods and actinobacillus actinomycetemco mitans respectively	IOs with semi-standardized film holder at implant installation, abutment connection, 90d and 180d.	-	-	-	180	No obvious bone resorption in any group	-	-	-	-	-
32. Tillmann s et al	1997	Ligature, submarginal	Cotton, braided retraction cord (GingiBrai d, VanR Dental Products, Oxnard, CA)	NA (although type and brand mentione d)	Only if necessary at plaque control appointmen ts	3m 6 dogs<br>6m 8 dogs	Ligatures on one side, continued plaque control on the other side of the mandible	CAL, PPD at baseline and then monthly	Periotest at baseline and then monthly	No (reported in separate paper)	No	No (reported in separate study)	No (reported in separate study)	-	90 (n=14) and 180 (n=8) respectivel y after ligature placement	NA, only CAL measures provided	-	CAL measured with probe	-	-	3 implants lost, 1 of each kind.
33. Abraham sson et al	1998	Plaque accumulation	-	-	-	5m	-	"redness, swelling and bleeding on gentle probing in all systems"	-	-	-	LM analysis of ground sections. Localisation, vertical extension and area of ICT. Marginal bone position with abutment/fixture junction as reference	Fractions of ICT infiltrated with Collagen, vascular structures, fibroblasts, macrophages, lymphocytes, plasma cells, PMN cells and residual tissue.	-	150	NO BASELINE RECORDED Distance from abutment-fixture (Polished-TPS in ITI-implants) junction to marginal bone:	-	Histo. Mean + sd	-	-	None reported

																1: 0,64 ± 0,44<br>2: 0,64 ± 0,72<br>3: 0,67 ± 0,25					
34. Eke et al	1998	Ligature	Silk (braided)	NA	No	6m	Ligatures around implants and natural tooth	PPD, Pi, Redness, BoP monthly	-	Dark-ffield microscopy and culture of various bacteria. Correlation with clinical findings and bone loss	X-rays at 3m, 6m	-	-	-	180	-	-	-	-	-	-
35. Hürzeler et al	1998	(A) Ligature<br>(B) Ligature + repeated overload<br>(C) Repeated overload<br>(D) Control	Silk, Overload	NA	New ligatures every 4w	Ligature: 32w Overloading: 16w (initiated 16w after ligature placement	Brush with pumice mixed with 2% CHX 3 times/w	-	-	-	-	Ground sections in bucco-lingual direction. Distance fromimplant top to first mineralised bone-implant contact. Mean value of buccal and lingual side. Percentage of mineralized bone-implant contact leangth	-	-	224	Ligature: 2,3±0,6,<br>Ligature+overload: 2,6±0,7,<br>Overload: 1,3±0,4,<br>Control: 1,1±0,5.<br>(224d ligature + 112d overload)	-	Histo.<br>Mean value of buccal and lingual aspect of each implant	-	1,1±0,5	None
36. Isidor	1998	(1) Overload on one side, (2) Ligature on other side and symphysial region (marginal position)	Cotton cord	NA	-	18m	(1) Brush once/ week. Debridement once/ month. (2) No hygiene	-	Periotestand manual mobility every 3m	-	IO. Parallelling technique	Ground sections<br>Marginal bone loss<br>BIC	-	-	540	(1) Periotest: 2 implants removed due to mobility. 6 remaining with values 6, 18, 45, 25, -7, -7. Almost complete bone loss in all but 2 implants with 1,8 and 1,9 mm bone loss resp.<br>(2) Periotest: -6, -5, -6, -7, -2, -5, -5, -4, -6, -5	-	Light microscopic analysis of ground sections	-	-	2 implants removed at abutment surgery<br>2 lost during overload phase
37. Miyata et al	1998	Overload (about 100 µm) 1, 2, 3, 4, weeks vs control	-	-	-	1. 2. 3 and 4w	No overload	No inflammatory responses at end of study	-	-	-	Marginal bone loss measured	Inflammatory cell infiltrate assessed	-	7, 14, 21, 28	Marginal bone loss (mm): 2,4 (0,8-4)<br>Lingual loss = L<br>Buccal loss = B<br>Control: L 1,62 B 1,15<br>7d: L 1,90 B 1,20<br>14d: L 1,76 B 1,21<br>21d: L 1,67 B 0,86<br>28d: L 1,66 B 1,18	-	Histo.<br>Buccal and lingual surfaces reported separately.	-	-	
38. Tillmanns et al	1998	Ligature, submarginal	Cotton, braided retraction cord (GingiBraid, VanR Dental Products, Oxnard, CA)	NA (although type and brand mentioned)	Only if necessary at plaque control appointments	3m 6 dogs<br><br>6m 8 dogs	Ligatures on one side, continued plaque control on the other side of the mandible	No (reported in separate paper)	No (reported in separate paper)	DNA from deepest pocket of each implant at baseline and then monthky. Different thresholds used for extent of microbes. P.gingivalis P. intermedia Actinobacillus actinomycetemcomitans	Periapical at baseline and monthly	Ground sections	Bone height and subsequent vertical or horizontal bone loss.<br><br>Thickness of HA coating		90 (n=6) and 180 (n=8)) respectively after ligature placement	90 days - 6 dogs<br>HA ctrl: 0,87±0,44. Test: 2,64±2,76<br>TPS ctrl: 1,41±0,98. Test: 2,53±1,02<br>Ti-A ctrl: 1,57±0,75. Test: 2,90±1,41<br><br>180 days - 8 dogs<br>HA ctrl: 1,72±1,18. Test: 2,85±1,76<br>TPS ctrl: 1,46±0,90. Test: 2,21±0,94<br>Ti-A ctrl: 1,84±0,43. Test: 2,18±0,42	Fig 11, 12 in paper	Histo.<br>Mesiodistal sections. Not specified whether an average of mesial and distal/ or greatest value was used.	-	-	3 implants lost, 1 of each kind.

39. Machado et al	1999	Lugatures, submarginal technique	Cotton	NA	No	1m	Ligatures around all implants	-	-	-	-	-	-	-	30	Bone loss not specifically measured	-	-	-	-	-
40. Persson et al	1999	Ligature	Cotton	NA	New ligatures after 1m and 2m	3m	-	-	-	-	IO		-	-	90	Baseline<br>Average of 1,33±0,91, 1,60±0,47 and 1,42±0,76<br><br>30 days after ligature removal<br>Average of 1,47±0,91, 3,37±0,20 and 3,54±0,67	-	Periapical x-rays with individual film holders prepared for each animal and site. Not specified whehter a mean- or max value was used for each implant.	-	-	-
41. Wetzel et al	1999	Ligature around implants	Silk	NA	NA	4m	Ligatures around all implants	-	-	-	IO at different intervcals	Saw sections of plastic embedded specimens	After treatment: Original defect and bone fill after GTR	-	120	(1) TPS: 3,22 mm<br>(1), (4): SLA: 2,77<br>(3) U: 3,18		Histo. Mesial and distal aspects investigated. Not specified whether an average of mesial and distal/ or greatest value was used.	-	-	2 lost during ligature phase. 6 lost after membrane phase and 1 during histological sectioning
42. Comut et al	2000	Ligature (2 of 4 dogs in the study. Other 2 euthanized before peri-implantitis induction)	Silk	NA	No	4w	Daily brushing	PPD (0,2 N standardized force, average depth from 4 measuring sites). No significant increase between groups	-	-	-	Ground sections.	Inflammatory cell count. Percentage of epithelium, connective tissue and bone apposed to the area of interest (the apical 1 mm of the transmucosal part ot the implant). Orientation of collagen fibers in the gingival connective tissue. More inflammatory cells in ligature, but no meaningful differences.	-	28	-	-	-	-	-	None
43. Machado et al	2000	Lugatures, submarginal technique	Cotton	NA	No	1m	Ligatures around all implants	-	-	-	-	-	-	-	30	Bone loss not specifically measured	-	-	-	-	-
44. Miyata et al	2000	Overload<br>100 µm<br>180 µm<br>250 µm<br>during 4w	-	-	-	4w	No overload	No inflammatory responses at end of study but increase PPD with increasing overload<br>(PPD, BoP)	Yes assessed clinically	-	-	Marginal bone loss measured	Inflammatory cell infiltrate assessed	-	28	No specific values provided<br>Control: No bone loss<br>100 µm: None<br>180 µm: To half the implant<br>250 µm: Almost to the apical end of the implant		Histological measrement			
45. Nociti et al	2000	Ligature, submarginal	Cotton	NA	No	1m	Ligatures around all implants	-	-	-	-	-	-	-	30	Amount of bone loss not reported	-	Histo	-	-	No implants lost

46. Shibutani et al	2000	Ligature, submarginal technique	Silk	NA	NA	12w	Ligaures around all implants. IM bisphosph onate injection (Pamidron ate 0,6 mg/ kg IM every 3 days) vs control	-	-	-	Pariapical	-	-	-	84	Clinical bone loss (mm) in:<br><br>Pamidronate group 1,59±0,55<br><br>Control group 2,41±0,48	NA	Clinical measurement with periodontal probe on the buccal side at center of the implant	-	-	-
47. Deppe et al	2001	Ligature	Cotton floss	NA	No	3m	Ligatures around all implants	-	-	-	Periapical X-rays Long cone technique with custom made film holder	Ground sections – computer assisted histometry: Size of former bone defect and reappositioned bone	-	-	90	Group 1: 1,7±0,8 min 0,00 max 2,90<br>Group 2: 1,7±0,9 min 0,1 max 3,70<br>Group 3: 1,7±0,5 min 0,9 max 3,30<br>(all 3 groups identical in PI but differing in subsequent treatment)	-	X-ray peripical, customized holder. Mean value of mesial and distal aspect of each implant.	-	-	-
48. McCracken et al	2001	2 years of normal loading (partial god denture) followed by Silk suture around 20 randomly selected implants	Silk suture	NA	No	6m	NA – Continued monthly plaque control?	-	-	-	Quantitative substraction technique	Plastic embedded ground sections – Bone to implant contact. Vertical marginal bone loss	-	-	180	Root form with ligature: 1.33 ± 0,30<br>Root form control: 0,71 ± 0,32<br>Plate form with ligature: 1,72 ± 0,47<br>Plate form control: 0,28 ± 0,13	-	X-ray, periapical, Quantitative subtraction technique. Average of mesial and distal bone loss for each implant	-	-	1 root form and 4 plate form implants failed to integrate
49. Nociti et al.	2001a	Ligature	Cotton	NA	NP	4w	-	-	-	-	-	BIC	-	-	182	Only BIC was measured	-	Histo	-	-	None reported
50. Nociti et al.	2001b	Ligature	Cotton	NA	NP	30d	-	Attachment level	-	A lot of bacterial species	-	-	-	-	30	Loss of attachment level 3.70±0.54	-	X-ray and histo	-	-	None reported
51. Nociti et al.	2001c	Ligature	Cotton	NA	NP	4w	-	Vertical bone fill	-	-	-	-	-	-	28	Bone loss was not measured, only vertical bone fill after different GBR approaches	-	Clinical	-	-	None reported
52. Nociti et al	2001d	Ligature, submarginal	Cotton	NA	No	4w	Ligatures around all implants	Boneloss measured before and after treatment	-	-	-	-	-	-	28	Bone loss was not measured, only vertical bone fill after different GBR approaches	-	Clinical	-	-	None reported
53. Persson et al.	2001a	Ligature	Cotton	NA	(1) 1 mo; (2) 2 mo	3m	-	-	-	-	IO	IC-BDc (mm), IC-PM (mm), PM-aJE (mm), aJE-CBI (mm), IC-CBI (mm), IC-BDh (mm)<br>Legends: implant shoulder (IC), bottom of the bone defect (BDc), the marginal level of bone in	-	-	90	Turned: 3.1±0.5 SLA: 3.2±0.3 (120 days)	-	X-ray	-	-	-

												contact with the implant (CBI), the marginal portion of the periimplant mucosa (PM), the apical termination of the barrier epithelium (aJE), bottom of the defect (BDh)									
54. Persson et al.	2001b	Ligature	Cotton	NA	-	3-4 mo	-	-	-	-	IO	BIC (%)	-	-	90	Approx. 50% of bone was lost after 150 days. Measurements of re-osseointegration	-	Histo	-	-	-
55. Schou et al	2001	**4 first monkeys:** (1) Healthy group: Maintained hygiene VS (2) Mild mucositis/ gingivitis group: Discontinued hygiene and special cover screw **4 last monkeys:** (3) Severe mucositis/ gingivitis group: Discontinued hygiene and special cover screw FOR 8M + ligature during last 3w before sacrifice VS (4) Periimplantitis/ periodontitis group: Liagture for 7m + inoculation of P. Gingivalis 3t/ w for 2w starting 1m after ligature placement	Silk, braided (Ethicon)	3-0	Ligatures pushed further down the pocket or changed 1t /m	3w and 7m respective ly for groups (3) and (4)	See the method box: 4 groups	Width of keratinized gingiva, PI, BoP, PPD, CAL with Peri-probe (40 g at 3 mm and 30g at 9 mm. (Peri Probe; Samhall Pile Dental, Sweden)	-	-	IO, long cone	Probe tip glued to specimen at sacrifice. Histologic probing depth and Probe tip to alveolar bone was assessed.	Cutting and grinding technique – general description of the different groups	-	210	See figure 7 in the paper, result presented in diagram, no numbers provided. 2-4 mm bone loss generally in the 7m ligature group, no bone loss in the other groups.	-	Probing. (Probe tip closer to bone around implants than teeth in all groups except (1) healthy controls	-	-	-
56. Deppe et al	2002	Ligature	Cotton floss	NA	No	3m	Ligatures around all implants	-	-	-	-	-	Determination of Ti deposition in oral mucosa, regional lymph nodes, spleen, liver, lung and kidney after 3 different treatments	-	90	-	-	-	-	-	-
57. Gotfredsen et al	2002	Ligature (submarginal) and loading	Cotton	NA	Yes, every 4w	16w+8w continued plaque accumulat	Inter-quadrant. 3 groups: (M+L)	-	-	-	Peri-apical X-rays. Custom made film	Ground sections	Fluorochrome labels (Calcein green 15mg/kg injected iv at w50 and w52; Alizarin Complexone 25mg/kg injected iv w56 and w58)	-	112	2,5 (at 112d/ ligature removal)	-	X-ray peripical, customized holder. Mean	-	-	-

						ion followed by 12 weeks of lateral loading	mucositis +load (P) Peri-implantitis Loading (P+L) Peri-implantitis +load				holder. Every 2 weeks between w24 and w60		Bone density measured: Higher in loaded implants. No significant differences between TURNED and SLA					value of mesial and distal aspect of each implant.			
58. Miyata et al	2002	**Test (2 monkeys):** Overload 250 µm + no hygiene control for 4w, then hygiene and no overload for an additional 4w. **Neg. control (1 monkey):** Hygiene and no overload for 8w **Pos. control (1 monkey):** Overload and no hygiene for 8w	-	-	-	4 and 8 w respectively	No overload. Weekly hygiene.	No mobility and no BoP in test and neg. control monkeys. BoP and moderate pus discharge in Pos. control.	-	-	-	Marginal bone loss measured	Inflammatory cell infiltrate assessed	-	28, 56	Test monkeys: Bone resorption reaching the apical third Neg. control: No bone resorption Pos. control: Bone resorption reaching the apical third	-	Histo	-	-	-
59. Schou et al	2002	Submarginal ligature and supragingival cross elastics and inoculation of P. Gingivalis	Silk, braided (Ethicon)	3-0	NA	9-18 m until 4-6 mm marginal bone loss	Ligatures around all implants	Witdh of keratinized mucosa, PI, BoP, PPD, CAL	-	-	IO, long cone	-	-	-	270-540	NA, since ligature period differed among animals	-	X-ray, periapical, quantitative digital subtraction Not specified whehter a mean- or max value was used for each implant	-	-	2 maxillary implants lost due to mobility during PI-induction
60. Schou et al	2002	ubmarginal ligature and supragingival cross elastics and inoculation of P. Gingivalis	Silk, braided (Ethicon)	3-0	NA	9-17 m until 4-6 mm marginal bone loss	Ligatures around all implants	PI, BoP, PPD, CAL	-	-	IO, long cone	-	-	-	270-510	NA, since ligature period differed among animals	-	X-ray, periapical, quantitative digital subtraction Not specified whehter a mean- or max value was used for each implant	-	-	1 mandibular implant removed before PI-induction due to mobility
61. Schou et al	2002	1-2 submarginal ligatures + 2-4 supragingival orthodontic elastics tied around implants. + Inoculation of	Silk, braided (Ethicon)	3-0	Ligatures pushed further down the pocket or changed 1t /m	14-22 m to achieve 4-6 mm bone-loss	Ligatures around all implants	-	-	-	-	-	-	-	420-660	NA, since ligature period differed among animals	-	-	-	-	3 maxillary implants removed during PI induction

62. Schou et al	2002	P. gingivalis 3t /w for 2w + 1w after 1w interuption starting 1m after ligature placement. 1-2 submarginal ligatures + 2-4 supragingival orthodontic elastics tied around implants. + Inoculation of P. gingivalis 3t /w for 2w + 1w after 1w interuption starting 1m after ligature placement.	Silk, braided (Ethicon)	3-0	Ligatures pushed further down the pocket or changed 1t /m	14-22 m to achieve 4-6 mm bone-loss	Ligatures around all implants	-	-	-	-	-	-	-	420-660	NA, since ligature period differed among animals	-	-	-	-	3 maxillary implants removed during PI induction
63. Shibli et al	2003	Ligature, submarginal and sutured in the peri-implant mucosa for retention	Cotton floss	NA	Further ligatures on top of old every 20d	60d	Ligatures around all implants	-	-	Before ligature placement and at 20d, 40d and 60d after ligature placement. Cultivation of various bacteria	Periapical, long cone technique at ligature placement and 20d, 40d and 60d after ligature placement	-	-	-	60	(1) cpTi 2,09±1,70<br>(2) TPS 1,70±1,52<br>(3) HA-coated 1,94±1,59<br>(4) hybrid turned+acid etched 1,62±1,32	NA	X-ray, periapical. Average of mesial and distal aspect of each implant	-	-	NA
64. Shibli et al.	2003	Ligature, Cotton floss, submarginal and sutured to gingiva. Followed by a 12m plaque control phase	Cotton floss	NA	Further ligatures on top of old every 20d	60d	Ligatures around all implants	-	-	Before and after treatment	-	-	-	-	60	NA (approx. 40%, not further specified)	-	-	-	-	Only 19 of 36 implants still integrated after ligature pahse + 12m hygiene phase
65. Shibli et al	2003	Ligature, submarginal and sutured in the peri-implant mucosa for retention	Cotton floss	NA	Further ligatures on top of old every 20d	60d	Ligatures around all implants	-	-	Before ligature placement and at 20d, 40d and 60d after ligature placement. Cultivation of various bacteria	Periapical, long cone technique at ligature placement and 20d, 40d and 60d after ligature placement	-	-	-	60d with ligature + 260d hygiene program	NA since measurement was carried out 12m after ligature removal. Approx 40% bone loss att removal. Not further specified	NA	Histomorpho metrics	-	-	17 implants lost after 60d ligature + 12m plaque control phase
66. Zechner	2003	Ligature – polyfiber polyester sutures (Mersilene) under 2.0 stainless steel wire ligatures	Polyester and stainless-steel wire	Mersilene : 2-0<br>Stainless-steel: 2-0	No	4m	No ligatures on contrl side. Continued oral	GI and PPD before ligature placement and monthly	-	-	IOs monthly from 2-nd stage surgery until end of study	4 dogs, 23 implants: Ground sections<br>4 dogs, 24 implants: light microscopic immunochemistry and	Active and previous bone resorption. Active bone formation and presence of fluorochrome labels.	-	120	See fig 4 in paper. Bone loss measured as a radiologic score	-	X-ray, concentional periapical technique. A score from -3 (severe bone	-	-	1 implant lost. Signs of injury after previous gingival probing around non-

		under the abutments					hygiene until end of experiment.					electron microscopy: Reported elsewhere						bone loss) to +3 (extensive bone apposition) was used.			ligated implants. (p 27)
67. Deppe et al	2004	Ligature	Cotton (floss)	NA	No	3m	Ligatures around all implants	Pressure-froced pocket probing in anesthesized animals	-	-	Periapical X-rays, long cone technique. Conventional film VS digital	Ground sections – computer assisted histometry	-	-	90	Conventional X-ray: 2,92±0,51 Digital X-ray: 2,97±0,44 Histology: 3,29±0,50	-	Conventional X-ray with indiviaual template Digital X-ray with individual template Not specified whether a mean value or max value has been used for each implant	-	-	-
68. Martins et al	2004	Ligature (submarginal position) followed by 1 year of supragingival plaque control	Cotton floss	NA	Further ligatures at 20 day intervals	60 days or at 40% bone loss	Ligatures around all implants	Pi, GR, BoP, PPD, CAL,	Periotest	-	IO for vertical and horizontal bone loss	-	-	-	Baseline to 20d 40d 60d	Baseline TPS: 2,50 ± 0,61 HA: 2,01 ± 0,46 AE: 2,36 ± 0,54 cpTi: 2,40 ± 0,51 20 days TPS: 3,85 ± 0,95 HA: 3,62 ± 0,29 AE: 3,64 ± 0,17 cpTi: 4,12 ± 0,72 40 days TPS: 4,62 ± 0,90 HA: 4,65 ± 0,84 AE: 5,19 ± 0,51 cpTi: 5,20 ± 0,71 60 days TPS: 6,00 ± 0,70 HA: 6,22 ± 0,50 AE: 6,06 ± 0,27 cpTi: 6,32 ± 0,00	Available in paper	Periapical x-rays, standardized with individual bite-blocks. Average of mesial and distal aspects of each implant. Baseline value greater than 0 due to ref. point above the bone crest. Subtraction of baseline value from reseptive measurement values will give amount of bone loss	-	-	None reported
69. Persson et al.	2004	Ligature	Cotton	NA	-	3m	-	-	-	-	IO	BIC (%), IC-PM (mm), PM-aJE (mm), aJE-CBI (mm), BC-BD (mm), re-osseointegration (mm), re-osseointegration (% of bone defect), defect area (mm^2), bone fill (mm^2), bone fill (%), regenerated bone (%),	-	-	90	Measurements of re-osseointegration, not bone loss	-	Histo	-	-	None reported

												old bone (%). Legends: the implant shoulder (IC), the marginal level of bone in contact with the implant (CBI), the marginal portion of the periimplant mucosa (PM), the apical termination of the barrier epithelium (aJE), and the marginal position of the lateral bone wall of the defect (BC)									
70. Zitzmann et al	2004	Ligature, submarginal + 12 m spontaneous plaque accumulation	Cotton	NA	Every 2w	2m (+12 m spontaneous plaque accumulation)	Ligatures around all implants				IO at baseline, ligature removal (2m) and after spontaneous accumulation (14m)	Ground sections of resin blocks – various measurements of peri-implant bone and soft tissues	ICT, plaque and pus areas measured	-	60+365	Bone loss after ligature removal: 2,58±0,39 mm Additional bone loss after 12m accumulation: 1,02±1,62	-	X-ray, periapical, mean of mesial and distal aspect	-	-	1 implant lost during ligature phase. 2 implants lost during spontaneous accumulation phase
71. Hayek et al	2005	Ligature	NA	NA	No	120d + 120d additional plaque accumulation	Ligatures around all implants	Clinical status and radiographs	-	-	-	One implant in 1 animal removed and investigated with SEM to validate implant contamination	One implant in 1 animal removed and investigated with SEM to validate implant contamination	-	120+120	-	-	-	-	-	1
72. Martins et al	2005	Ligature (Submarginal position) + 1 year passive phase with daily cleaning	Cotton floss	NA	Further ligatures at 20 day intervals	60 days	Ligatures around all implants	PD, CAL	Periotest	-	IO for vertical and horizontal bone loss	-	-	-	Baseline to 20d 40d 60d 425d	Baseline cpTi: 2,32 ± 0,53 TPS: 2,50 ± 0,61 HA: 2,01 ± 0,46 Acid: 2,36 ± 0,54 20 days cpTI: 4,12 ± 0,72 TPS: 3,85 ± 0,95 HA: 3,62 ± 0,29 Acid: 3,64 ± 0,17 40 days cpTI: 5,20 ± 0,71 TPS: 4,61 ± 0,90 HA: 4,65 ± 0,84 Acid: 5,19 ± 0,51 60 days cpTI: 6,32 ± 0,33 TPS: 6,00 ± 0,70 HA: 6,22 ± 0,50 Acid: 6,06 ± 0,27	Available in paper	Periapical x-rays, standardized with individual bite-blocks. Average of mesial and distal aspects of each implant. Baseline value greater than 0 due to ref. point above the bone crest. Subtraction of baseline value from reseptive measurement values will give amount of bone loss	-	-	None during ligature phase, 17 during the 1 year follow up
73. Sennerby et al.	2005	Ligature, submarginal	Cotton	NA	Every 3w	3m	Ligatures around all implants	-	Resonance frequency	-	Standardized IOs with individualiz	Re-osseointegration after treatment measured	-		90	SLA group 3,1±1,2 U group	-	X-ray, periapical, standardized	-	-	NA

									analysis (RFA) at baseline, after ligature phase and after treatment		ed film holders					3,4±0,3		with individual film holder			
74. Stübinger et al	2005	Ligature	Cotton	NA	No	3m	Ligatures around all implants	-	-	-	-	-	-	-	90	Noticable circumferential peri-implant bone defects. Not further specified	NA	NA	NA	NA	NA
75. Trejo et al	2005	Ligature 1w + plaque accumulation 2w + ligature 3w + 2m with different hygiene protocols:<br>(a) mechanical cleaning<br>(b) mechanical cleaning + 0,2% CHX gel + 0,12% CHX irrigation<br>(c) No hygiene	Silk	NA	Ligature 1w + plaque accumulation 2w + ligature 3w	Ligature 1w + plaque accumulation 2w + ligature 3w	Ligatures around all implants	PI, GI, PPD, CAL, recession, BoP<br>PPD, CAL, recession from 6 ponts around the implant to nearest mm	-	-	-	Saw sectioned plastic embedded specimens	Bone level and various other measurements	-	7+14+21	NA, only CAL measures provided	-	-	-	-	1 implant not inserted due to lack of stability at implant surgery.
76. Watzak et al	2005	Spontaneous plaque accumulation	-	-	-	18m	3 implant systems, all treated in the same way	All implants had signs of peri-implant inflammation after 18m	-	-	-	Ground sections – severe inflammation with comparatively little crestal bone loss around all implants. Lymphocytes, plasmacells and macrophages dominated inflammatory infiltrates. Minor difference between implant types	Mucosal margin<br>apical extension of sulcus and junctional epithelium,<br>implant-abutment connection, first bone-implant contact	-	540	Maxilla:<br>All implant types: 0,9 mm (0,5/1,6 CI)<br>CpTi screw: 0,9 (0,5/1,6)<br>TPS cylinder: 0,9 (0,5/1,5)<br>GBAE screw: 0,9 (0,5/1,6)<br>Mandible:<br>All implant types: 0,6 mm (0,4/0,9) to 0,8 mm (0,5/1,2)<br>CpTi screw: 0,8 (0,5/1,2)<br>TPS cylinder: 0,6 (0,4/0,9)<br>GBAE screw: 0,7 (0,5/1,6)	-	Histo - each implant measured on 3 sites: buccal and lingual + mesial or distal	-	-	No implants and no suprastructures lost
77. Berglundh et al	2006	Ligature (sub-marginal position) +spontaneous accumulation	Cotton	NA	Every 2 weeks	4m with ligature and 5m with additional plaque accumulation	-	-	-	-	Periapical X-rays	LM analysis of ground sections. Greater amount of bone loss at the SLA sites than at Polished sites. (7,34 vs 5,95 mm)	-	-	120	SLA: 2,51± 0,55<br>Polished: 2,27±1,05		X-ray periapical, customized film holder. Mean values for each variable	Additional loss 5 months after ligature removal:<br>SLA: 1,12±1,07<br>Polished: 0,07±0,72	-	None reported

78. Kozlovsky et al	2006	(A) No ligature, no overload (B) No ligature, overload (C) Ligature no overload (D) Ligature and overload	Cotton floss	NA	Replaced every 4w	12m	Brushing with 2% CHX 3 times/w	PI, GI, PPD monthly	Periotest monthly	-	Periapical radiographs every 3m	Ground sections – BIC, Vertical bone level from implant top and bony crest, horizontal bone level	-	-	365	Buccal implant margin to bottom of bone defect: (A) 0,37 ± 0,54 (B) 0,12 ± 0,61 (C) -2,53 ± 0,77 (D) 3,08 ± 0,89 Lingual implant margin to bottom of bone defect: (A) 0,80 ± 0,68 (B) 0,50 ± 0,64 (C) -2,50 ± 0,65 (D) -3,28 ± 0,31	Buccal intrabony defect horizontal distance: (A) 0,39 ± 0,23 (B) 0,41 ± 0,34 (C) 1,20 ± 0,35 (D) 1,69 ± 0,72 Lingual intrabony defect horizontal distance: (A) 0,72 ± 0,57 (B) 0,53 ± 0,19 (C) 2,20 ± 0,07 (D) 2,35 ± 0,33	Histo. Bucco-lingual sections, both measurements reported separately	-	Registered in vertical and horizontal bone loss boxes respectively	All implants stable throughout the study (-0,76 mean periotest value)
79. Schwarz et al	2006	Ligature, submarginal	Cotton	NA	Every 3w	At approximately.. 40% bone loss (around 3m)	Ligatures around all implants	BOP, PPD, Gingival recession, CAL at 5 aspects per implant	-	-	Periapical before and 3m after treatment	Ground sections. New bone after surgery	Various measurements on both soft and hard tissues	-	NA	NA, ligatures removed at approx 40% bone loss at approx 3m	-	-	-	-	No losses
80. Shibli et al	2006	Ligature, submarginal and sutured in the peri-implant mucosa for retention	Cotton floss	NA	Further ligatures on top of old every 20d	90d	Ligatures around all implants	-	-	-	-	-	-	-	90	(1) Turned 2,43±0,96 (2) TPS 4,55±1,77 (3) Hybride 2,60±0,96 (4) Sandblasted with Ti-oxide 2,8±0,41	-	Intraoperative measurement with periodontal probe, after reflection of soft tissues. Mean value of 4 sites around each implant.	-	-	2 implants in 2 animals lost during ligature phase: (1) cpTi (Turned) test group (4) (Sanblasted Ti-oxide) control group
81. Schwarz et al	2007	Ligature, submarginal vs naturally occurring PI in humans	Cotton	NA	Every 3w	At approximately.. 30% bone loss (around 3m)	Ligatures around all implants	Measurements of bone defect during flap surgery	-	-	-	-	-	-	Approximately 90	Distribution, configuration and size of defects in table 2 of paper 79% configuration between natural vs experimental PI. Generally a combined supracrestal and intrabony defect.	Distribution, configuration and size of defects in table 2 of paper	Open flap surgery	-	-	
82. Takasaki et al	2007	Man made buccal defect with silicone later exchanged for stainless steel mesh + ligature	Silk	NA	No	Defect + silicone 3m. Steel mesh + ligature 4w	Same for all implants			PCR – samples from ligature at time of removal for various bacteria	-	-	Area of new bone, % of new bone height, 5 of new bone to implant contact	-	90+28	Not relevant since defect was partly man made	Not relevant since defect was partly man made	-	-	-	NA
83. You et al	2007	Ligature around the implants	Gauze and wire	NA	NA	2-4m / animaldue to different	Ligatures around all implans	-	-	-	IO	-	-	-	60-120	4-6 mm bone loss	-	IO radiographs	-	-	-

84. Albouy et al	2008	Ligature (sub-marginal position) +spontaneous accumulation	Cotton	NA	At weeks 3, 6, and 9	rates of bone loss 12w (40-50% boneloss) Plaque accumulation continued for 24 weeks after ligature removal	-				IO before ligature placement. At ligature removal. At 6, 14 and 24 weeks after ligature removal.	-	-	-	84	Baseline at ligature placement. Bone loss at ligature removal:<br>A: 3.53 ± 1,04<br>B: 4.10±0,63<br>C: 4,69±0,52<br>D: 3,58±0,37	-	X-ray peripical, customized holder. Mean value of mesial and distal aspect of each implant.	ADDITIONAL bone loss 24 weeks after ligature removal:<br>A: 1,84±1,41<br>B: 1,72±1,25<br>C: 1,55±0,68<br>D: 2,78± 1,91	-	A: 1 implant lost at week 26.<br>D: 1 lost at week 35.
85. Martines et al	2008	Ligature, submarginal position	Cotton, (GN Injecta, Diadema, Brazil)	NA	No	90d	Ligatures around all implants	CAL, PPD,	Periotestuntil same value twice	-	-	-	-	-	90	Only tables – no specific figures. More bone loss around Straumann implants after 90d. Main loss of CAL during the first 30 days of ligature phase. Mobility increased in both groups	-	-	-	-	3 Brånemark and 3 Straumann implants lost during implant healing phase
86. Albouy et al	2009	Ligature (sub-marginal position) +spontaneous accumulation	Cotton	NA	At weeks 3, 6 and 9	12w (40-50% boneloss) Plaque accumulation continued for 24 weeks after ligature removal	-	-	-	-	-	LM analysis of ground sections. Localisation, vertical extension and area of ICT. Marginal bone position with abutment/fixture junction as reference	Discussed in general terms	-	84	NA	-	Light microscopy	-	-	A: 1 implant lost at week 26.<br>D: 1 lost at week 35.
87. Parlar et al.	2009	Ligature	Cotton	NA	1m	3m	-	-	-	-	IO	(1) linear measurement from the coronal-most BIC level to the base of the implant body; (2) linear measurement from the alveolar bone crest level to the base of the implant body; and (3) percentage of BIC on the surface of the basal implant body	-	-	90	-	-	Histo	-	-	None reported
88. Albouy et al	2011	Ligature (sub-marginal position)	Cotton	NA	At weeks 3, 6 and 9	12w (40-50% boneloss)	-	-	-	-	-	-	-	-	84	3.87 (12 weeks)	-	X-ray peripical, customized holder. Mean value of mesial and distal aspect of each implant.	-	-	D: 1 lost 2 months after placement.

89. Levin et al	2011	Ligature retained with cyanoacrylate around all implants	Silk	NA	No	45d	None. Ligatures around all implants	-	-	-	-	-	-	Implants removed and reimplanted.	45	-	-	-	-	-	Removed implants reimplanted at fresh sites, wider implants att old sites. 10 succesful, 6 lost.
90. Schwarz et al	2011	Ligature – submarginal technique	Cotton	4-0	Every 4w.	4 months approx. (until 60% bone loss)	Ligatures around all implants	Intra-operative assessment of defects during PI surgery	-	-	CBCT	Ground sections – Implant shoulder to bottom of bone defect and the most coronal level of bone in contact with the implant surface. Percentage linear histological bone fill calculated on buccal and lingual aspect.	-	-	NA	NA since ligature period was not specified	In table 1	Intra-operative measurement	-	-	No losses
91. Albouy et al	2012	Ligature (sub-marginal position) +spontaneous accumulation	Cotton	NA	Every 3 weeks	10w (12 according to Fig 1 text) Plaque accumulation continued for 26 weeks after ligature removal	-	-	-	-	IO periapical X-rays	LM analysis of ground sections. Localisation, vertical extension and area of ICT. Marginal bone position with abutment/fixture junction as reference	-	-	70	Marginal bone loss from IO X-rays:<br>At ligature removal (10 weeks):<br>A: 3,00 ± 0,44<br>B: 3,47 ± 0,45	-	X-ray peripical, customized holder. Mean value of mesial and distal aspect of each implant.	Additional bone loss 16 weeks after ligature removal:<br>A: 0,03 ± 0,50<br>B: 1,47 ± 0,65	-	None reported
92. Golubovic et al	2012	Ligature (submarginal position) + failed regenerative surgery	Cotton	4-0	Yes, every 4w	4m	-Ligatures around all implants	-	-	-	CBCT	Ground sections.	-	-	120	NA	-	-	-	-	-
93. López-Piriz et al	2012	Ligatures, submarginal technique	Cotton	NA	Replaced every 3w	3m	Ligatures around all implants	Assessment of plaque and inflammation weekly	-	-	Periapical radiographs	-	-	-	90	(Redcued variable) test<br>Distal test 1,33±1,56<br>Distal ctrl 3,47±3,01<br>Mesial test 2,04±3,72<br>Mesial ctrl 3,73±4,55	-	"X-rays, periapical with holder that allowed easy and predictable allignment. Mean of mesial and distal aspect"	-	-	1 case implant lost at abutment connection
94. Becker et al	2013	Ligature	Vicryl	4-0	No	9w	Same, no ligature.	-	-	-	Micro-CT	LM analysis of ground sections	-	-	63	NA, only vertical measurements provided	-	Micro-CT	-	Bone defect size 0,15±0,015	2 from the ligature group; 1 from the control group
95. Carcuac et al	2013	Ligature (Sub-marginal position) at implants and 2nd, 3rd and 4th	Cotton	NA	At weeks 3 and 6	10w + 26w continued plaque accumulation	Ligatures around all implants and teeth	-	-	-	Periapical X-rays with customized film-holder	LM analysis of groundsections,	Immunohistochemical analysis of paraffin sections.	-	70	"Turned: 2,69±0,57<br>Ti-Unite 3,14±0,69	-	X-ray peripical, customized holder. Mean value of mesial and	Additional loss 26 weeks after ligature removal:<br>Teeth: 0,00±0,53<br>A: -0,02±0,66<br>B: -1,34±1,19	-	None reported

		premolars in left mandible																distal aspect of each implant.			
96. Fan et al	2013	Ligature	Cotton infiltrated with P. gingivalis	NA	Yes, every 2w	1,5m	Ligatures around all implants	PPD + Bop 3m post implantation and after 1,5m ligature time. Greater PPD in groups B and C	NA	-	X-rays every 2w during ligature period	Ground sections. 3-buccolingual sections from each implant.	-	IgG and IgA antibody response	45	Boneloss: Group A<B, C Group B<C Successful immunization in groups A and B.	-	X-rays and histometrics	-	-	-
97. Madi et al	2013	Ligature, submarginal position	Silk	NA	Replaced every 3w	4m with ligature + 5m spontaneous plaque accumulation after ligature removal	Ligatures around all implants	PPD, CAL, modified GI at 1,2,3,4,5, and 9 m with periodontal probe at fixed points marked on the abutments	-	-	Yes, long cone technique at baseline, 4m and 9m	-	-	-	120+150	Radiograph mesial: (1) 1±0,6 (2) 1,1±0,7 (3) 1,1±0,5 (4) 1,7±0,6 Radiograph distal: (1) 0,9±0,6 (2) 0,92±0,6 (3) 0,83±0,5 (4) 1,6±0,6	-	X-rays, periapical with custom-made long cone parallelling device. Mesial and distal aspects reported separately	Minor changes in CAL and PPD after 5 months spontaneous accumulation following ligature removal	-	none
98. Charalampakis et al	2014	Ligature (Sub-marginal position) at implants and 2nd, 3rd and 4th premolars in left mandible	Cotton	NA	At weeks 3 and 6	10w + 26w continued plaque accumulation	Ligatures around all implants and teeth	-	-	DNA-DNA hybridization for 16 bacterial species (14 dog strains and 2 human strains). At baseline, ligature removal (10 weeks) and study end (36 weeks)	-	-	-	-	70	Radiogprahic parameteris published in Carcuac et al 2013	-	-	-	-	-
99. Guo et al	2014	Ligature (Tied around neck)	Cotton infiltrated with Pg ATCC 33277	NA	Yes, every 2w	1,5m	Ligatures around all implants	Clinical indexes, BOP	-	-	-	Ground sections. Qualitative assessment of bone-loss and peri-implantitis	Qualitative assessment of bone-loss and peri-implantitis since bone level at ligature placement was not definite	-	45	Bone loss in all groups, but mostly in groups C&D	-	-	-	-	-
100. Madi et al	2014	Ligature, submarginal position	Cotton	NA	Replaced every 3w	4m with ligature + 5m spontaneous plaque accumulation after ligature removal	Ligatures around all implants	Configuration of bone defects assessed with periodontal probe after removal of soft tissue	-	-	Periapical x-rays before removal of ligatures. No specific measurements	-	-	-	120+150	Figure 4a+b, page 667	-	Clinical measurement during surgery (soft tissue removed)	-	-	none
101. Martinez et al	2014	Ligature (submarginal position)	Cotton	NA	Replaced every 3w	3m and 13w both mentioned	Ligatures around all implants	-	-	-	-	Plastic embedded ground sections Abutment fixture junction, Gingival margin, crestal bone margina, coronal position of bone to implant contact	See paper	-	90	Uncoated controls: Lingual 3,2 ± 0,71 Buccal 2,8 ± 0,20 Coated mesial implant: Lingual 2,8 ± 0,53 Buccal 2,5 ± 0,27 Coated distal implant:	-	Histo. 2 central bucco-lingual sections from each implant	-	-	One distal coated implant lost

																Lingual 2,7 ± 0,56<br>Buccal 2,6 ± 0,19					
102. Pârvu et al.	2014	Ligature	Cotton	NA	-	3w	-	-	-	-	IO	-	Counting of neutrophils and mononuclear cells	Blood samples, fractal analysis, serum nitro-oxidative stress evaluation	21	NA. Bone loss not specifically measured	-	-	-	-	None reported
103. Battula et al	2015	Ligature (Sub-marginal position, placed during abutment surgery.)	Silk	3-0	No	12w and 26w	Daily brushing and rinsing with 0,12% CHX.	PPD	-	-	Periapical X-ray at implant installation.	Blinded LM analysis of ground sections.	Acute inflammation, chronic inflammation and fibrosis evaluated on a 5-point scale. 0=not present, 1=minimal, 2=mild, 3=moderate, 4=marked (accoarding to Loe and Silness index)	-	84+182	Measurements in pocket depth, not in bone loss	-	Clinical	-	-	None reported
104. Carcuac et al	2015	Ligature (sub-marginal position)	Cotton	NA	At weeks 3 and 6.	9w	Ligatures around all implants	-	-	4 weeks after ligature removal (hygiene reinstated at ligature removal) DNA-DNA hybridization tech	Periapical X-rays	-	-	-	63	TiOblast: 3,58±0,76<br>OsseoSpeed: 3,72±0,65<br>At-I: 3,73±0,47<br>TiUnite: 3,57±0,63	-	X-ray peripical, customized holder. Mean value of mesial and distal aspect of each implant.	-	-	None during the breakdown phase
105. Fickl et al	2015	Ligature (submarginal position)	Silk	NA	Additional ligatures every 2w until totally 4 ligatures per implant	8w followe by 3w of daily hygiene accordnig to table one (2), plaque control method	Ligatures around all implants	PPD. BoP. Mucosal recession. Depth and width of intrasurgical marginal bone defect to nearest 0,5 mm.	NA	-	Periapical with customized film-holder. Mesial and distal bone defects measured.	-	-	-	56	Table 1, page e665. Only specifies difference between implants. Radiographic bone level and intra-surgical defect depth displayed in fig. 6 but no numbers are provided	-	X-rays, periapical with customized film holder. Mean value of mesial and distal aspect of each implant. Clinical measurement of 4 surfaces. Not specified whehter a mean- or max value was used for each implant.	-	-	One implant (S) mobile and removed before ligature placement
106. Huang et al	2015	Ligature (submarginal position)	Cotton floss	NA	No	12w	Ligatures around all implants	CAL, PPD, PI, BoP at baseline 6w and 12w	-	-	Standardized periapical radiographs	-	-	-	84	"0,39±0.39 (crestal screwed-in)	Crestal implants:<br>(A) 0,59±0,55<br>(B) 0,55±0,45	X-rays periapical with	-	-	None

																0,24±0,36 (crestal tapped-in) 0.97±0.85 (subcrestal screwed-in) 0.73±0.47 (subcrestal tapped-in)	Subcrestal implants: (A) 0,84±0,91 (B) 0,92±0,57	templates, average of mesial and distal value for each implant			
107. Ikumi et al	2015	Static compressive load at 0-180 MPa	-	-	-	7d	-	-	-	-	-	Ground sections	-	-	7	No bone loss in any if the implants	-	Light microscopy	-	No bone loss	None
108. López-Piriz et al	2015	12w spontaneous accumulation followed by 10w of submarginal ligatures	Cotton	NA	Replaced every 3w	10w (preceded by 12 w spont. Accumulation)	Same procedure on both sides.	-	-	CFU/ml and cultivation	Periapical radiographs	-	-	-	84+70	See fig 8 After spontaneous accumulation (84d): Control 0,5±0,44 G3 0,45±0,39 ZnO35 0,8±0,77 G1n-Ag 1,2±0,47 Total bone loss after ligature (84d+70d): Control 2,21 ± 0,46 G3 1,64±0,43 ZnO35 1,42±0,40 G1n-Ag 1,45±0,56 Bone loss during ligature phase total bone loss - spontaneous accumulation loss	-	X-rays, periapical with holder that allowed easy and predictable allignment. Mean of mesial and distal aspect	-	-	1 G1n-Ag lost at sec surgery
109. Namgoong et al.	2015	Ligature	Stainless steel	NA	NP	23w	-	-	-	-	IO (5, 8, 12, 18, 23w), after GBR (4, 8, 12w)	PPDD, BRH, BRA, BIC	-	-	161	Turned: 2.4 ± 1.0 (XR) 2.7±1.2 (H) SA/HA: 2.7 ± 0.6 (XR) 3.5±1.2 (H) SA: 2.3 ± 1.0 (XR) 3.2±1.4 (H) (23 weeks)	-	IO X-ray and histometric analysis	-	-	None reported
110. Park et al.	2015a	Ligature	Orthodontic ligature wire + 3-0 silk	0.254 mm + 3-0	Additional ligature every 4 weeks (no exchange)	4m	-	-	-	-	IO	(a) linear measurement from the alveolar crest to the apex of the implant, (b) linear measurement from the most coronal bone-to-implant contact (first BIC) level to the apex of the implant, (c) linear measurement from the most coronal BIC to the bottom of the defect (height of reosseointegration), (d) linear measurement from the implant shoulder to the bottom of the bone defect (defect depths), (e) vertical bone fill, (f) BIC ratio	-	SEM, EDS	120	NA, but approx. 40% of bone was lost after 120 days.	-	x-rays	-	-	None reported
111. Park et al.	2015b	Ligature	Braided cotton	1.58 mm	No	4m	-	-	-	-	-	(1) mineralized tissue area (mm2) including the newly formed bone and bone graft material; (2) alveolar bone height (mm) measured from the apex to the most coronal	-	-	120	NA, but approx. 40% of bone was lost after 120 days.	-	Histometric analysis	-	-	None reported

												position of the alveolar bone; (3) first bone-to-implant contact (first BIC) height (mm) measured from the apex of the implants to the first BIC position; (4) re-osseointegration height (mm) as the first BIC height from the bottom of the defect; (5) ratio of linear re-osseointegration calculated as the ratio of re-osseointegration height to defect depth; (6) BIC (%) within re-osseointegrated bone as the ratio of total perimeter of bone contact to the whole thread perimeter (mm) between the bottom of the defect and the first BIC position; and (7) BIC ratio (%) in the residual bone as the ratio of the total perimeter of bone contact to the whole thread perimeter (mm) below the bottom of the defect.									
112. Pirih et al	2015	Ligature	Silk	6-0	No	12w	Control animals without ligature	Gingival swelling	Bucco-lingual wiggling forces applied to implants after 4w	-	Micro-ct	Ground sections.	Ground sections	-	84	Distance from apical point of implant head to first bone contact, (4 lost ligature implants counted as 1 mm bone loss = total implant leangth):<br><br>Ligature group = n10 0,579±0,0490<br><br>Control group = n8 0,226±0,016	NA	Micro CT	-	-	4 ligature implants lost but included in the statistics.
113. Pirih et al	2015	Injcetion of P. gingivalis LPS. (1) No injected control (2) vehicle injected control (3) LPS-injected exp. Group. Injection in distopalatal peri-implant mucosa twice/w	-	-	-	6w	No injection. Vecicle injection	Exposed implant head area.		-	Micro ct	Cutting and grinding sections	See histology section	-	42	Implant shoulder to bottom of defect:<br><br>Non injected: 217 micro m<br>Vehicle injected: 230 micro m<br>LPS-injected: 320 micro m	-	Micro-Ct	-	-	none

114. Shi et al	2015	Ligature, submarginal	Cotton with intertwined stainless steel wire of 0,25 mm diameter	NA	Every 3w	At approximately 40% bone loss	Ligatures around all implants	At baseline (ligature removal)+ 1, 2, 3 m after ligature removal: BOP, PPD	-	At baseline (ligature removal) and 1, 2, 3 m: samples from 4 sites per implant. 1 positive site = positive implant	CT and micro-CT at baseline (ligature removal) and 3m	Ground sections. 4 buccal-lingual sections per implant. Distance rom first bone-implant contact to implant apex	-	-	NA	NA, ligatures removed at different time points	-	-	-	-	No losses
115. Takamori et al	2017	Immunization (I) and then palatal injection of Lippolysaccharide (LPS) in I vs non-immunized (nI) rats vs PBS injection in I rats vs PBS injection in nI rats	-	-	-	34d total	5 implant groups + 5 tooth groups<br>(1) Baseline group<br>(2) nI-PBS<br>(3) I-PBS<br>(4) NI-LPS<br>(5) I-LPS	-	-	-	-	No of inflammatory cells, osteoclasts etc	Distance from first implant thread to 1. gingival crest, 2. Apical end of junctional epithelium and 3. Bone crest.	-	34	See fig 9 in paper. Significantly longer distance from first thread to bone crest in immunized LPS group compared to other groups. No attachement loss around teeth in any group.	-	-	-	-	-
116. Htet	2016	Ligature (submarginal position) 3X3 MM BUCCAL DEFECT MADE ATT IMPLANT INSERTION	Cotton with silk	NA	New ligatures inserted every 2w	4w (until approx. 40% bone resorption)	-	-	-	-	Long cone with custom made paralleling device	-	-	-	28	NA, not measured	-	-	-	-	4 implants lost after 3w of experimental periimplantitis
117. Ishii et al	2016	Ligature	Dental floss	NA	-	90d	Same as experimental side	-	-	-	(1) Standardized dental radiographs (70kv, 15mA, 0,25s). (2) Micro-CT	Ground sections	-	-	90	(1)<br>UV-group: 2,0±0,5 mm<br>Controls: 2,7±0,4 mm	(2)<br>Area of bone resorption mm2<br>UV-group: 45,7±9,6<br>Controls: 64,4±10,6	(1) Dental x-rays Dental X-rays, standardized technique with silicone bite block mean of mesial and distal aspect of each implant<br>(2) Micro-CT	-	(1) 2,7±0,4	-
118. Machtei et al	2016	Ligature, submarginal retained with cyanoacrylate around all implants	NA (retraction cords)	NA	No	2m	Ligatures around all implats	-	-	-	-	-	-	-	90	NA. not measured	2-4 mm wide (not further specified)	-	-	-	-
119. Madi et al	2016	Ligature, submarginal position	Silk	NA	No	4m	Ligatures around all implants	-	-	Yes – during ligature phase to confirm presence of	-	Yes, implant shoulder to first bone contact. BIC etc.	SEM to investigate residual HA coating.	-	120	Marginal bone loss extending to the coronal or middle third of all implats. Not further specified.	-	-	-	-	-

										periodontophatic bacteria.											
120. Nguyen Vo et al.	2016	Ligature	Silk	5-0	NP	0, 1, 2, 3, 4w	-	-	-	IL-1<br>TNF-α<br>GAPDH	Micro-CT scans	Bone level	-	-	0, 7, 14, 21, 28	***Bone height (Micro-CT)***<br>0: 0.81±0.04 (mesial)<br>0: 0.84±0.03 (distal)<br>4w: 0.37±0.03 (mesial)<br>4w: 0.37±0.07 (distal)<br>***Bone height (histo)***<br>0: 0.9±0.06 (buccal)<br>0: 0.84±0.09 (palatal)<br>4w: 0.53±0.03 (buccal)<br>4w: 0.45±0.04 (palatal)	-	Micro-CT scans and histometric measurements	-	-	None reported
121. Xu et al	2016	Ligature – submarginal	Cotton	NA	Ligatures repositioned monthly	3m (at approx. 40% bone loss)	Ligatures around all implants	PD, CAL	-	-	IO at baseline, ligature				90	4.6 mm mean bone loss	-	X-ray, paralleling technique	-	-	2 lost during ligature phase
122. Godoy-Gallardo et al	2016	Ligature, submarginal	Silk	4-0	NA	2m	Ligatures around all implants	PPD, mucosal recession, keratinized ginigiva, CAL, PI, BoP	-	-	Periapical X-rays, micro-ct	Bone and tissue resorption with light microscopy. SEM also used.	Ground sections	-	30, 60	At ligature placement<br>Ti_Ag: 2,9±0,6<br>Ti_TSP: 2,8±0,5<br>Ti: 3,0±0,6<br><br>30 days<br>Ti_Ag: 3,5±0,4<br>Ti_TSP: 3,6±0,5<br>Ti: 3,9±0,7<br><br>60 days<br>Ti_Ag: 4,1±0,5<br>Ti_TSP: 4,0±0,5<br>Ti: 4,6±0,7		X-ray, periapical, standardized mean of mesial and distal aspect	-	-	No losses
123. Park et al.	2017	Ligature	Braided cotton	1.58 mm	Additional ligature every 4 weeks (no exchange)	4m	-	IS-BD	-	-	IO	(1) the mineralized tissue area (mm2), including the newly formed bone and bone graft material; (2) coronal bone loss (IS-BD) from the implant shoulder (IS) to the bottom of the defect (BD); (3) defect depth (BC-BD) from the bone crest (BC) to BD; (4) the re-osseointegration height (mm) as the most coronal BIC level from BD; (5) vertical bone fill (%) as the ratio of the re-osseointegration height to IS-BD; (6) total perimeter of BIC (mm) within the re-osseointegrated bone, and (7) the BIC ratio (%) within re-osseointegrated bone as the ratio of (1) the mineralized tissue area (mm2), including the newly formed bone and	-	-	120	***IS-BD (clinical)***<br>Immediate model<br>3.88±0.99 (mesial)<br>3.88±0.99 (distal)<br>5.38±1.30 (buccal)<br>3.38±0.52 (lingual)<br>Conventional model<br>3.50±0.53 (mesial)<br>3.13±0.35 (distal)<br>4.00±0.53 (buccal)<br>3.13±0.35 (lingual)<br>***IS-BD (histological)***<br>Immediate model<br>6.02±1.20 (buccal)<br>4.41±1.07 (lingual)<br>Conventional model<br>4.34±0.86 (buccal)<br>3.81±0.61 (lingual)<br>***BC-BD (histological)***<br>Immediate model<br>0.01±0.00 (buccal)<br>1.06±0.73 (lingual)<br>Conventional model<br>0.11±0.20 (buccal)	-	Histometric analysis	-	-	None reported

												bone graft material; (2) IS-BD from the IS to the BD; (3) BC-BD from the BC to BD; (4) the re-osseointegration height (mm) as the most coronal BIC level from BD; (5) vertical bone fill (%) as the ratio of the re-osseointegration height to IS-BD; (6) total perimeter of BIC (mm) within the re-osseointegrated bone, and (7) the BIC ratio (%) within re-osseointegrated bone as the ratio of the total perimeter of the bone contact to the whole thread perimeter (mm) between BD and the first BIC position				1.20±0.58 (lingual)					
124. Lin et al	2017	Ligature – Stainless steel ligature wrapped 6 turns around the implant neck and forced as deeply into the pocket as possible	Stainless steel	0,010 inch	No	12w	Ligatures around all implants	PPD at 4 aspects per tooth baseline, ligature removal and 4w after ligature removal	-	-	Standardized periapical radiographs at 3w and 12w	-	-	-	21, 84	21d (mm)<br>1,6±0,6<br><br>84d<br>4,0±0,8	Width of bone defect mm (linear distance between the 2 peaks of the saucer-shaped defect, minus the diameter of the implant):<br><br>3w: 4,9±1,2<br><br>12w: 6,8±0,8	X-ray, periapical, film holder connected to tube. Average of mesial and distal values	-	-	No loose or lost implants at ligature removal time piont
125. Koutouzis et al	2017	Bacterial inoculation with P. gingivalis, Treponema Denticola, Tannerella forsythia by means of gingival lavage	-	-	-	24 inoculations during a 6w period	6/12 animals were sham inoculated	-	-	Yes, 16S ribosomal RNA	Micro-ct	-	-	-	42	Infected (n= 4 implants): 0,80±0,72<br>+2 infected rats had both implants failed<br>Sham-infected (n= 3 implants): 0,48±0,13		Micro-CT. Distance between implant platform to first BIC.			majority of implants in both groups were excluded before study end, for reasons outside the treatment, such as aspiration: Three rats with 4 implants in the infection group and two rats with 3 implants in the sham-infection group were analyzed

126. Moest et al	2017	Ligature, sub-marginal	Cotton	NA	Every 3w	2m	Ligatures around all implants	-	-	-	-	-	-	-	60	Not measured	-	-	-	-	No implants lost
127. Ramos et al	2017	Ligature, around healing abutment	Silk, Ethicon	3-0	Only replaced when missing at weekly controls	8w	Ligatures around all implants	-	-	-	-	-	-	-	56	Not measured at ligature removal, but first after 6w of additional plaque accumulation	-	-	-	-	3 implants failed to osseointegrate, 5 lost during PI induction, 1 lost during histologial processing
128. Rodrigue z et al	2017	Ligature, sub-marginal	Metal wire	NA	No	12w	Ligatures around all implants	-	-	-	-	Crestal bone loss and histological pocket depth	Ground sections. Light microscopy. Inflammatory infiltrate, CT fiber attachment	-	84	Laser-microtextured implants: 1,95 Resorbable blast textured implants: 2,72	-	Histo. Mean of mesial and distal measurement s	-	-	3 RBT and 1 LM implants lost due to instability at abutment placement
129. Wong et al	2017	Ligature, sub-marginal	Silk (P:B:N: Medical, Steløse, Denmark)	6-0	No	4w	No ligature	General description of degree of soft tissue edema.	-	-	Micro-CT	-	Decalcified sections. Osteoclasts counted, collagen assessed	-	14	"ligature Implants: 0,431±0,019 non-ligature implant: 0,218±0,012"	-	Micro-ct. Average of 4 measurement s per implant	-	-	NA
130. Yu et al	2017	Ligature, sub-marginal	Silk (Fischer Scentific, Waltham, MA, USA)	7-0	No	2w	5 wild type mice killed directly after implantati on, another 5 after 4w healing and 5 + 2 Tlr2-knock out after 6w post implantati on	-	-	-	Micro-CT	-	Decalcified sections	PCR analys is of palatal soft tissues	14	"Wild type (n = 41 implants) lost: 15 Loose: 4 Integrated: 22 see Fig 2b bone loss vol. Toll like 2 receptor knock out (n=21 implants) Lost: 3 Loose: 0 Integrated: 18 see fig 2b."	-	Micro-CT	-	-	See vertical bone loss
131. Hiyari et al	2018	Ligature, sub-marginal	Silk	6-0	No	1w and 4w resepctive ly	No ligature	Presence or absence of implants, mobility. No difference between groups	-	-	Micro-CT	-	Undecalcified ground sections and decalcified sections	-	7 and 28	Volume of circumfrential bone loss in fig 1 D and E. Significant difference between strains	-	Micro-CT	-	-	-
132. Hiyari et al	2018	Ligature, sub-marginal	Silk	6-0	No	1w, 1m, 3m respective ly	No ligature	General description of degree of soft tissue edema.	-	-	Micro-CT	-	Undecalcified and decalcified sections	-	7, 30, 90	Figure 2c in paper	-	Histological sections, average of mesial and distal sites	-	-	Fig. 3 Volymetric bone loss less in 3m compared to 1m in implants and in teeth at 1m and 3m

																					compared to 1w. Seems to follow osteoclast activity (fig. 5)
133. Huang et al	2018	Ligature, sub-marginal	Cotton	NA	No	12w	Ligatures around all implats	General description	-	-	Yes but method not specified. Mesial and distal bone loss assessed radiographically	Assessment of supra-alveolar bone loss, infrabony defects, marginal bone loss.	Undecalcified ground sections in the bucco-lingual plane	-	84	"Distance from bone ridge to first bone-to-implant contact (Ridge-fBIC): Crestal astra implant: 0,19±0,25 Crestal Bicon implant: 0,34±0,30 Subcrestal Astra: 1,32±0,48 Subcrestal Bicon: 0,95±0,39"	Table 1 in paper	Histo + X-ray: Bucco-lingual sections. Average from measurements on buccal, lingual (histo) mesial and distal (X-ray) aspects. Bone ridge used as ref. point since implants were placed on both crestal and sub-crestal positions.	-	-	No losses

NA = not available; X = not investigated; Pi = plaque index; GR = gingival redness; BoP = Bleeding in probing; PPD = pocket probing depth; CAL = clinical attachment level; TPS = titanium plasma sprayed; HA = hydroxyapatite, U = ~~uncoated~~ turned/machined; IO – intraoral; ICT = Infiltrated connective tissue; MBL – marginal bone level
LM: Light microscope, GI = Gingival index

Table S3. Main characteristics of the studies included in the quantitative synthesis.

									Type of implant				Surgical protocol							Pre- and postoperative care			
Author	Published	Species	Comparison (X vsY)	No. of animals	Jaw	No. of extracted teeth	Healing period (m=months, w=weeks)	Study design (split mouth, inter quadrant)	Shape (cylindrical/ screw-type)	Size (witdh/ Length) (mm)	Material	Coating	Bone level/ Tissue level	Nr of implants/ animal	1 stage/ 2 stage surgery	Implant center to center distance (mm)	Time implant healing + abutment-PI induction	Randomisation	Loading	Antibiotic	Plaque control timing (before/ after induction)	Plaque control duration (months)	Plaque control method
1. Lindhe et al	1992	Dog, Beagle		5	Mandible	PM1 PM2 PM3 PM4 On the right side	3m	Split mouth, implants on left side, teeth on the right	Screw shaped Brånemark	NA	Ti	Turned	Bone level	3 on the right side of the mandible	2-stage	NA	3m+6m	No	NA (No suprastructure)	NA	(A) After abutment connection	(A) 6m	(A) Brush and dentifrice daily
2. Lang et al	1993	Monkey, cynomolgus	Plaque accumulation vs ligature	4	Mandible	6: PM2 M1 M2 On bot sides	90d	Interquadrant: One implant with ligature and one without in each quadrant	Cylindrical ITI type F	6,0x3,0 mm)	Ti	TPS	Tissue level	4: 2 on each side of the mandible	1-stage	NA	60d+0d	NA	No	NA	(A) After implant placement (B) From day 60-	(A) 60d (B) 30d	(A) Rubber cup and pumice 3 times/ w. (B) Suspende

																							d plaque control
3. Cook et al	1995	Dog, Mongrel	2 implant surfaces	14	Mandible, implants on both sides	4 premolars on each side of the mandible	2m	Interquadrant	Cylindrical (Calicitec implants)	4x10	Ti	A: Cancellous-structured titanium B: HA-coated	Bone level	6 (3 on each side. 2 implants were not inserted due to anatomical reasons)	2-stage	NA	8w+0	NA	NA (although no suprastructure was used)	5 days postoperatively	None at the test side. At the time of abutment connection on the control side.	From abutment surgery to end of study	Weekly brushing
4. Persson et al.	1996	Dog, Labrador	TREATMENT STUDY	5	Mandible	PM3, PM4, M1 on both sides of the mandible	3m	Split mouth	Screw type, Brånemark, Nobel Biocare, Göteborg, Sweden	3,75x10 mm	Ti	U	Bonelevel	6: 3 on each side	2 stage	-	3m+3m	No	-	1 month after ligature removal: Amoxicillin 375mg x2 for 3w	(1) After abutment surgery	(1) 3m	(1) tooth and abutment cleaning 3t /w.
5. Fritz et al	1997	Monkey (Macaca mulatta)	Plate-from vs root-form implants vs natural teeth	36	Mandible, both sides	All mandibular molars	6m	Comparison between animals	Plate-form Screw-shaped (Osseodent. Collagen Corporation, Palo Alto CA. According to Fritz 1994)	Screw-shaped: 3,75x7 Plate form: 14x7x1,6	Ti	Na	Bone level	12 natural teeth (2nd molar), 11 plate-form (2nd molar region, 12 root form implants (2nd molar egion)	2-stage	-	6m+12m	Yes.	Yes, Fixed bridge placed min 3 m after implantation	Penicillin G procaine, 300,000 post operatively	At the time of suprastructure connection, min 6 months after implant installation	12m	Monthly cleaning, polishing of implants and teeth and implant scaling with plastic scaler
6. Hanisch et al	1997	Monkey, Macaca Mulatta	Experimental peri-implantitis	4	Maxilla and mandible	All premolars	3m	Maxilla vs mandible	Cylindrical	NAx10 Bio-vent Dentsply	Ti	HA-coated	Bone level	8: 2 in each quadrant	2-stage	NA	12m+5m	No	NA (no suprastructure)	NA	(A) 2m after abutment connection (B) 11m after baseline (1m afterligature removal)	(A) 3m (B) 4w	(A) Brusch + 2% CHX daily (B) Brusch+ 2% CHX 3times/w
7. Abrahamsson et al.	1998	Dog, Beagle	3 implant systems: 1. Astra tech Implants, Dental System. 2. Nobel biocare, Brånemark System. 3. ITI dental implants system	5	Mandible	All mandibular premolars. (1st 2nd and 3rd maxillary molars also extracted to avoid occlusal interferences)	3m	One of each fixture in each quadrant in a randomized order	Screw-type	1: 3,5x8 2: 3,75x7 3: 4x8	Ti	1: NA (TiOblast?) 2: NA (TiUnite?) 3: TPS	1 and 2: Bonelvel 3: Tissuelevel	6	1-2: 2-stage (submerged) 3: 1-stage (tissuelevel)	NA	3m + 1m	Yes	No	NA	After implant installation	4m	Daily brushing and dentifrice
8. Hürzeler et al	1998	Monkey, cynomolgus	Repetitive mechanical trauma ± ligature	5	Mandible	All mandibular PM:s and	12w	Ligatures on one side of the mandible	Screw shaped	3,75x7 mm Brånemark	Ti	U	Bone level	8:4 on each side of the mandible	2-stage	NA	16w+4w	Yes ligature side chosen by flipping a coin	Yes, single crowns	-	(A) First week after abutment (B) Second week after abutment	(A) 1w (B) 3w	(A) 2% CHX swabbing 3 times/ w

						M1, M2																	(B) Brush + flour of pumice and 2% CHX spray 3 times/ w.
9. Tillmanns et al	1998	Dog, Beagle	Bone loss around 3 differrent implant systems with and without ligatures	16	Mandible	PM2, PM3, PM4 On both sides	3m	Inter-quarant – 1 of each implant type on each side	(a) cylindrical (b) cylindrical (c) screw-form	(a)-(c) 4x 10 mm	(a)-(c) Ti-6A1-4V	(a) Calcitite HA coating (Sulzer Calcitek, Carlsbad, CA) (b) Commercially pure TPS coating (APS Materials, Dayton OH) (c) Machined Ti-alloy surface (Sulzer, Calcitek)	Bone level	6: 3 on each side	2-stage	NA	3m+4w	YES, one implant of each type randomly placed on each side of the mandible<br><br>Ligature side also randomly selected	No – suprastructure on neithbouring teeth used to prevent implant loading	NA	(A) After abutment surgery	(A) 1m on ligature side. 4m on control side (until sacrifice)	(A) Brushing, interproxmal brushin and scaling with graphite scaler 3t/w. With sedation every 2w when necessary
10. Persson et al.	1999	Dog, Beagle	TREATMENT STUDY	4	Mandible	PM1, PM2, PM3, PM4 on each side	4m	Different positions (inter-quadrant)	Screw type, Brånemark, Nobel Biocare, Göteborg, Sweden	7 mm leangth	Ti	U	Bonelevel	6: 3 on each side	2 stage	-	4m+2m	No	-	1m after ligature removal,(Imacillin 250 mg x2) for 3w	(1) After abutment surgery	(1) 2m	Tooth and abutment cleaning every 2nd day
11. Deppe et al	2001	Dog, Beagle	TREATMENT STUDY	6	Mandible, both sides	NS	NA	1 of 3 treatment methods in each hemimandible	Cylindrical (Frialit 2)	3,8x11	Ti	Ti plasma spray coated	Bone Level	10 5 on each side)	2-stage	NA	3m+4w	No	NA (no suprastructure	NA	At abutment connection	4w	Oral hygiene
12. Persson et al.	2001a	Beague dogs	Turned x SLA surface	4	Mandible	8: 4 each side (1ºM, 2ºPM, 3ºPM, 4ºPM)	12m	Split-mouth	Screw-type (ITI Straumann, Waldenburg, Switzerland)	3.3x8.0	Ti	U (left side) SA (right side)	Tissuelevel	6	1 stage	-	3m + 0	No	-	5 weeks after removal of ligatures, for 17 days	(1) After implant installation; (2) 1 month after treatment	(1) 3m (2x/week); (2) 6m	(1) and (2) Tooth and implant cleaning
13. Shibutani et al	2001	Dog, Beagle	Ligature induced bone loss with-without IV Pamidronate	10	Mandible	PM2, PM3 on the left side	6m	Comparison between animals	Screw shape (TiOblast, Astra Tech AB, Mölndal, Sweden)	3,5x 11 mm	Ti	TiOblast	Bone-level	2 per animal	2-stage	NA	4,5m+3w	NA	No	NA	(A) After abutment connection	(A) 3w	(A) Brush and 0,12% CHX rinse 1t/ w.
14. Shibli et al	2003	Dog, Mongrel	Microbiology and bone loss after ligatures around 4 different implants	6	Mandible	All mandibular and maxillary PMs	3m	Inter-quadrant	Screw-shape (1) Commercially pure Ti (3i) (2) TPS (ITI/ Straumann, Esthetic plus (3) HA-coated (Calcitek) (4) Hybrid: first 3 threades	(1), (3), (4): 3,75 x 10 mm<br><br>(2) 4,1 x 10 mm	Ti	(1) Turned (3i) (2) TPS (ITI/ Straumann, Esthetic plus (3) HA-coated (Calcitek) (4) Hybrid: first 3 threades machined, the rest acid etched (Osseotite, 3i)	Bone-level	6: 3 on each side	2-stage	NA	90d + 45d	Yes, at least one implant of each kind in each animal	No	Potassium and sodium benzyl penicillin once/w for 2w postoperatively	(A) 2w before extraction<br><br>(B) After tooth extraction	(A) at one time<br><br>(B) 225d (until 45 days after abutment connection)	(A) Oral hygiene<br><br>(B) Scrubbing with 0,12 % CHX daily and scaling and root

									machined, the rest acid etched (Osseotite, 3i)														planning 1t/m.
15. Deppe et al	2004	Dog, Beagle	Assessment of reliability of 3 bone attachment measurement methods	6	Mandible, both sides	NS	3m	Reliability of 2 X-ray methods and pressure-forced probing,	Cylindrical (Frialit 2)	3,8x11	Ti	Ti plasma spray coated	Bone level	10 (5 on each side)	2-stage	NA	12w+4w	NA	NA (no suprastructure)	NA	At abutment connection	4w	Oral hygiene
16. Martins et al.	2004	Dog, Mongrel	4 surfaces	6	Mandible	8 premolars (4/side). (maxillary also extracted to avoid occlusal trauma interference)	3m	≥ one of each implant in each animal	Screw-type	3,75x10 4,1x10	Ti	TPS, HA, U + AE (U in 3 first threads and AE in other threads), U	Bone level	6: 3 on each side of the mandible	2 stage (submerged)	10	3m + 45d	No	No	24000 IU/kg benzylpenicillum 1x/week for 2 weeks after implant placement	Before PI	9,5 (2 weeks before extraction to ligature placement.)	Daily chlorhexidine scrubbing + Scaling and root planning 1x/month
17. Martins et al	2005	Dog, Mongrel	4 surfaces	6	Mandible	8 premolars (4/side). (maxillary also extracted to avoid occlusal trauma interference)	3m	≥ one of each implant in each animal	Screw-type	3,75x10 4,1x10	Ti	TPS, HA, U + AE (U in 3 first threads and AE in other threads), U	Bone level	6: 3 on each side of the mandible	2 stage (submerged)	10	3m + 45d	No	No	24000 IU/kg benzylpenicillum 1x/week for 2 weeks post op (after implant placement – OTHERWISE, POST-OP. OF WHICH SURGERY? THIS MIGHT BE CONFUSING FOR THE READER IF NOT INDICATED	(A) Before PI (B) After PI	(A) 9,5 (2 weeks before extraction to ligature placement.) (B) 12m	(A) Daily chlorhexidine scrubbing + Scaling and root planning 1x/month (B) same as A
18. Berglundh et al	2007	Dog, Beagle	2 surfaces	5	Mandible, both premolar regions	All mandibular premolars	3m	Split mouth	Screw shaped. Straumann SP	3,3x8	Ti	Sandblasted Acid Eched Vs Polished	Tissue level	6	1-stage	NA	3 months and 2 weeks + 0	No	No (Not specifically stated, no extraktion of antagonizing teeth reported)	NA	2w after implant installation	3m minus 2w	Daily cleaning of implants and teeth with toothbrush and 0,12% CHX gel.
19. Shibli et al	2006	Dog, Mongrel	TREATMENT STUDY: Lethal photosensitization and GBR	5	Mandible	All PMs and M1 on both sides of	3m	Split mouth	Screw shape (1) CpTi Sterngold Implantmed,	3,75x10 mm	Ti	(1)Turned (2) TPS,	Bone level	8: 4 on each side	2-stage	NA	3m+2m	Yes according to treatment	No – upper PMs extracted to avoid loading	otassium and sodium benzyl penicillin once/w for	(A) After tooth extraction	(A) until ligature placement	(A) Scrubbing with 0,12 % CHX daily and

						the mandible			Attleboro, MA, USA)<br>(2) TPS, Sterngold…<br>(3) Hybrid – machined in first 3 screws and then acid etched (3i Osseotite, Implants Innovations<br>(4) Sandblasted with ti oxide Porous, Conexao Implants…			(3) Hybrid – machined in first 3 screws and then acid etched<br><br>(4) Sandblasted with ti oxide								2w postoperatively			scaling and root planning 1t/m.
20. Albouy et al	2008	Dog, Labrador (same animals as study 6 in this table)	4 implants/ -surfaces (A, B, C, D)	6	Mandible, right side	12 (All mandibular premolars and the three anterior premolars in the maxilla on both sides)	3m	Interquadrant: One of each implant type in the same quadrant	Screw type<br>A: Biomet 3i ICE Micro miniimplant<br>B: Astra Tech MicroThreaded<br>C: Straumann SP NN<br>D: Nobel Biocare MKIII NP	A: 2,35x10<br>B: 3,5x11<br>C: 3,3x10<br>D: 3,3x10	Ti	A: U<br>B: TiOblast<br>C: Sandblasted AE<br>D: TiUnite	A,B,D: Bonelevel<br>C: Tissuelevel	4 (all in 1 quadrant)	1-stage	NA	3m + 0	Yes	No	NA	2w after implant installation	3m minus 2w	Daily cleaning of implants
21. Albouy et al	2012	Dog, Labrador	2 surfaces	5 according to abstract, 6 according to material and method	Mandible, one side	6 (All mandibular premolars and the 3 anterior premolars in the maxilla on one side.)	3m	Interquadrant: One of each in one side of the mandible	Screw type (Brånemark MKIII narrow platform)	3,3x10	Ti	Turned, TiUnite	Bone level	2	1-stage	NA	3m + 0	Yes	No	NA	2w after implant installation	3m minus 2w	Daily cleaning of implants
22. López-Piriz et al	2012	Dog, Beagle	Soda-lime-glass/ Ag abutment coating vs control	5	Mandible	PM1<br>PM2<br>PM3<br>PM4<br>M1<br>On both sides of the mandible	3m	Interquadrant: Test implants at all central and distal sites.	Screw shaped Phibo dental solutions	NA	Ti	?	Bone level	6: 3 on each side of the mandible	2-stage	NA	2m+	No	NA (no suprastructure)	No	(A) After abutment connection	(A)4w	(A) Toothbrush and dentifrice 5d/w
23. Carcuac et al	2013	Dog, Labrador (same animals as study	Teeth vs implants/ 2 implant surfaces	6 according to material and method, 5	Mandible, implants on the right side, teeth on the left	Right mandibular premolars and first	3m	Split mouth (IMPLANTS VS TEETH)	Screw shaped Nobel Biocare MKIII, NP	3,3x10	Ti	A: Turned<br>B: TiUnite	Bone level	4 (On the right side of the mandible)	1-stage	NA	3+0	Yes – Pairwise placement	No	NA	2w after implant installation	3m minus 2w	Tooth and abutment cleaning 3 times a week

		13 in this table)		according to absctract		molar. Three right anterior maxillary premolars		Interquadrant (2 SURFACES)															
24. Madi et al	2013	Dog, Beagle	Experimental PI at different implant surfaces	6	Mandible	PM1 PM2 PM3 PM4 On both sides of the mandible	3m	Interquadrant, anterior-posterior randomized fashion	3 screw shaped and 1 cylinrical - the (4) HA plasma-sprayed+pressurized	3,3x10 mm (screw shaped) and 3,25x10 mm (cylindrical)	Ti	(1) Machined (2) Sandblasted, acid-etched (3) Sputter HA-coat (4) HA plasma-sprayed+pressurized	Bone level	8: 4 on each side of the mandible	1-stage	NA	0+3m	YES, implants placed in a randomized order	No	Metronidazole 11mg/kg postoperatively for three days	(A) 2w after implant placement	(A) 3m	(A) 2% CHX rinse 3t/w+scaling 1t/m
25. Martinez et al	2014	Dog, Beagle	Soda lime glass coated abutments with silver Nano-particles	5	Mandible	PM1 PM2 PM3 PM4 M1 On both sides of the mandible	12w	Inter-qudrant: Mesial implant as control, 2 distal ones as test	Screw shaped (Phibo dental solutions, Barcelona, Spain)	3,75x11,5	Ti	NA	Bone level	6: 3 on each side of the mandible	2-stage	NA	2m+4w	No	No	No	(A) After abutment connection	(A) 4w	(A) brush and dentifrice 5d/w
26. Carcuac et al	2015	Dog, labrador	TREATMENT STUDY 4 implants/ surfaces. CHX vs Saline	6	Mandible, both sides	All mandibular premolars. 1st, 2nd and 3rd maxillary premolars	3m	Split mouth (CHX vs SALINE) Inter-quadrant (4 surfaces)	Screw shaped. A, B, and C: Astra Tech Implant system. D: Nobel Biocare.	A,B,C: 3,5x11 D: 3,3x11,5	Ti	A: TiOblast B: Osseospeed C: AT-I D: TiUnite	Bone level	8 (4 on each side of the mandible)	1-stage	NA	3+0	Yes – identical placementsequence on both sides but randomized sequence between dogs	No	NA	2w after implant installation	3m minus 2w	Plaque control 3 times a week
27. Huang et al	2015	Dog, Beagle	Bone defect configuration depending on implant placement depth and implant type	6	Mandible	PM2, PM3, PM4, M1 on both sides of the mandible	8w	Interquadrant: 2 of each implant type. 1 of each implant type placed 1,5 mm sub-crestally and the other 2 at bone level	Screw-type (OsseoSpeed, (A) Astra, Mölndal, Sweden and (B) Integra-CP. Bicon, Boston, USA I	(A)&(B) 3,5x8	Ti	(A) Fluoride modified TiOblast (B) Plasma-sprayed calcium-phosphate	Bone level and 1,5 mm sub-crestal level	4 on the left side, total of 24 implant in the study	2-stage	NA	12w+4w	Yes	No	For 7d after extractions.	(1) After tooth extraction (2) After implant insertion (3) 10d after abutment surgery	(1) 1w (2) 1w (3) 4w minus 10d	(1) & (2) Cleaning with 0,12% CHX solution (3) 0,12% CHX irrigation every 2nd day initially, then brush every 2nd day
28. Namgoong et al.	2015	Beagle dogs	3 surfaces: U, SA, SA/HA	5	Mandible	10: 5 each side (1ºPM, 2ºPM, 3ºPM, 4ºPM, 1ºM)	12w	Split-mouth	Screw-type (Osstem, Busan, Korea)	3.0x8.5	Ti	U, SA, SA/HA	Bonelevel (1 mm subcrestally)	3	2 stage (submerged)	NA	12w + 2w	Yes	No	NP	During implant healing: toothbrush 2x/w for 12w. Post-op.: NP	12w pre-op.	toothbrush

29. López-Piriz et al	2015	Dog, Beagle	3 different antimicrobial glassy coated abutments effect on bacteria, biofilm and bone loss	5	Mandible	All Ms and PMs on both sides of the mandible	3m	Inter-qudrant: (1)control (2) ZnO-glassy (3) G3 glassy coating (4) n-Ag coating	Screw shaped SEVEN, MIS	NA	Ti	?	Bone level	8; 4 on each side of the mandible	2-stage	NA	8w+	No	NA (No suprastructure)	NA	(A) After abutment connection	(A)4w	(A) Toothbrush and dentifrice 5d/w
30. Pirih et al	2015	Mice, C57BL/6 J male	Ligature (10 mice) vs control (8 mice)	18	Maxilla	3: M1, M2, M3 on the left side	8w	Comparison between animals	Screw type (G. Hartzell and Son, Concord, CA, USA)	0,5x1	Ti	U	Tissuelevel	1	1 stage	-	4w+0	Yes, toss of a coin	No	For 4w aft tooth extractions/ implant surgery respectively. Diluted in drinking water	No	No	No
31. Ishii et al	2016	Dog, Beagle	UV-light irradiated SLA-surface vs conventional SLA-surface	3	Mandible	PM2, PM3, PM4 on both sides	0m immediate post extraction implantation	Split mouth	Screw shaped	3,3x8 mm Straumann SP	Ti	SLA (sandblasted + AE)	Bone level	4: 2 on each side of the mandible	1-stage	NA	90d+0d	No	No	-	(A) After implant surgery	(A) 90d	(A) 0,12% CHX
32. Godoy-Gallardo et al	2016	Dog, Beagle	Implants with 3 surfaces: Ti, Ti_Ag Ti-TSP	5	Mandible	All PMs on both sides of the mandible	3m	Inter-quadrant	Screw-type (Soadco S.A., Escaldes-Engordany, Andorra)	3,5x8 mm	Ti	Ti-group: Sandblasted and acid etched Ti-Ag: same + silverelectrodeposition Ti-Tsp: same + TESPA sinlanisation	Tissue level	6: 3 on each side	1-stage	Min 3 mm between implants	2m+0	Yes, random implant placement	NA-no suprastructure	Amoxicillin post op.	(A) After implant insertion. (B) 10d after implant insertion	(A) 10d (B) 2m	(A) cleanin with CHX on gauze. (B) Brusch with CHX 3t/ w.
33. Park et al.	2017	Beagle dogs	Immediate vs. delayed implantation	4	Mandible	6: 3 each side (3ºPM, 4ºPM, 1ºM)	0 (3ºPM) and 3m (4ºPM)	Split-mouth	Screw-type (TSIII SA fixture; Osstem, Seoul, Republic of Korea)	3.5x8.5	Ti	SB/AE	Tissuelevel	4	1 stage (3ºPM) 2 stage (4ºPM)	NA	0 + 0 (3ºPM) 3m + 0 (4ºPM)	No	No	After GBR (3d)	2w after ligature removal	Only once	Dental water jet + flossing
34. Lin et al	2017	Dog, Beagle	Stainless steel ligature investigated	6	Mandible	All PMs on both sides of the mandible	4w	Ligatures around all implants	Screw-type (Straumann tissue levele)	3,3x8 mm	Ti	Sand blasted and acid etched (SLA)	Tissue level	6: 3 on each side, tot 36	1-stage	NA	12w+0	No	NA-no suprastructure used	30 min before all surgical treatment	(A) after abutment connection	(A) 12w	(A) brusch 2t/w
35. Koutouzis et al	2017	Rat, Wistar	Polymicrobial inocula by gingival lavage vs sham	12 (5 were analyzed)	Maxilla	M1, on both sides	1m	Comparison between animals	Screw-type custom made	1,5x2	Ti	Turned	Bone level	2: one on each side of the maxilla	2-stage	-	2m+1w	No	No	Yes, before for 4 consecutive days prior to inoculation	(A) Prior to inoculation	(A) 4d	(A) CHX swabbing

Ti = titanium; TPS = titanium plasma sprayed; HA = hydroxyapatite, U = ~~uncoated~~ turned/machined, AE – acid-etched, Before PI = prior to induction of experimental peri implantitis; After PI = following induction of experimental peri-implantitis; NA = Not available; CHX = Chlorhexidine

Table S4. Induction and outcome of experimentally induced peri-implant bone loss in studies included in the quantitative synthesis.

Induction of peri-implant bone loss								Diagnostic markers							Peri-implant bone defect						
Author	Published	Method (ligature/ overload/other)	Ligature material	Ligature size	Ligature exchange	Duration	Control side protocol	Clinical measurements (BoP/ PPD etc)	Mobility	Microbiological sampling (cultivation/ PCR)	X-ray	Histometric measurements	Histologic evaluation	Other	Registration (days from baseline/ ligature placement)	Vertical Bone loss Mean ± SD (mm)	Horizontal Bone loss Mean ± SD (mm)	Measuring method	Development after ligature removal (progression/ healing)	Bone loss ± SD (mm) at control side	Lost implants
1. Lindhe et al	1992	Ligature, submarginal Followed by 1 month plaque accumulation after removal	Silk	NA	Replaced after 3w	6w	Ligatures around 2 teeth	PI, BoP,	All teeth and implantsclinically stable at end of study	In Leonhardt et al, 1992	Periapical radiographs	Size and content of ICT reported in Morphometric measurements	Size and content of ICT reported in Morphometric measurements	-	32 with ligature + 30 with spontaneous accumulation	Implant 3,2±0,3 Tooth 1,1±0,6	-	X-rays, periapical with eggen holder. Not specified whehter a mean- or max value was used for each implant	-	-	None
2. Lang et al	1993	(A) Ligature implant (B) Spontaneous plaque accumulation implant (C) Natural tooth (M3)	Silk	NA	New ligatures on top of old ones at 3m and 6m	8m	None	PI, GI, CAL	-	-	Periapical radiographs	Supposed to have benn reported in subsequent study that couldn't be retrieved.	Supposed to have benn reported in subsequent study that couldn't be retrieved.	-	240 (baseline at ligature placement after 30d plaque accumulation)	Radiographic loss (240 days) (A) 1,01 ±0,74 (B) 0,36 ±0,31 (C) 0,64 ±0,67 Data from 30, 60, 150, 180 days also available in table 2 on page 8	-	Periapical x-rays, standardized with oral bite block. Mean of mesial and distal values.	-	Registred in vertical bone loss box	None reported
3. Cook et al	1995	Ligature (tightened between fixture and abutment, suture ends extended to oral cavity)	Silk	4-0	No	4w, 8w, 16w and 26w	Weekly brushing. No ligature	-	-	-	Routine dental radiographs	Ground sections - Bone and tissue apposition, porosity of coating on experimental implants, HA thickness on contral implants	Degree of inflammatory response (0-5): Minimal in both CSTi and HA at all time periods examined.	-	28+56+112+182	Histology results at 182d (5 animals): 14x CSTi: 1,96±0,94 28x HA: 2,69±1,27	-	Histo. Mean + SD	-		2 lost: One prior to abutment surgery, the other removed at 2nd surgery due to infection
4. Persson et al	1996	Ligature, submarginal	Cotton	NA	-	About 6w/ 20% radiographic bone loss	Ligatures on both sides	Bone loss measured with probe during PI-surgery.	-	-	IO	Marginal bone loss after GBR.	Cell composition and localisation of ICT	-	42	Implant shoulder to bottom of defect: 1,8±0,45 (30 days after ligature removal)	-	Periodontal probe, mean value of 4 sides per implant	-	-	-
5. Fritz et al	1997	Ligature	Silk (braided)	NA	No	6m	Ligatures around all implants, contralateral 2nd molar and non-ligated second premolar (anterior abutment)	Plaque score, redness, PPD	NA	-	Periapical with "quantitative subtraction method"	-	-	-	90+180	Root form implants: 90d: 1,35±0,24 180d: 1,72±0,28 Plate form: 90d: 1,59±0,44 180d: 1,89±0,45	-	"X-ray periapical with standardized technique Quantitative subtraction method"	-	-	-
6. Hanisch et al	1997	Ligature (submarginal)	Cotton	NA	Yes, every 1m	10m + 1m additional plaque	Ligatures around all implants	Modified PI, GI, BOP, PPD, CAL. Calibrated probe	Periotest, Siemens	Submucosal samples from 1 randomly chosen implant in maxilla	-	-	-	-	300	Maxilla: 3,3±1,2 Mandible: 3,4±1,4	Maxilla: 2,0±0,5 Mandible: 2,0±0,4 Overall:	Clinical measurement at abutment connection	-	-	1

						accumulation				and mandible respectively. Morphotype, culture and DNA-probe						Overall: 3,3±1,3 (2 months after ligature removal)	2,0±0,5	and at reconstructive surgery after removal of soft tissue			
7. Abrahamsson et al	1998	Plaque accumulation	-	-	-	5m	-	”redness, swelling and bleeding on gentle probing in all systems”	-	-	-	LM analysis of ground sections. Localisation, vertical extension and area of ICT. Marginal bone position with abutment/fixture junction as reference	Fractions of ICT infiltrated with Collagen, vascular structures, fibroblasts, macrophages, lymphocytes, plasma cells, PMN cells and residual tissue.	-	150	NO BASELINE RECORDED Distance from abutment-fixture (Polished-TPS in ITI-implants) junction to marginal bone:<br>1: 0,64 ± 0,44<br>2: 0,64 ± 0,72<br>3: 0,67 ± 0,25	-	Histo. Mean + sd	-	-	None reported
8. Hürzeler et al	1998	(A) Ligature<br>(B) Ligature + repeated overload<br>(C) Repeated overload<br>(D) Control	Silk, Overload	NA	New ligatures every 4w	Ligature: 32w Overloading: 16w (initiated 16w after ligature placement	Brush with pumice mixed with 2% CHX 3 times/w	-	-	-	-	Ground sections in bucco-lingual direction. Distance fromimplant top to first mineralised bone-implant contact. Mean value of buccal and lingual side. Percentage of mineralized bone-implant contact leangth	-	-	224	Ligature: 2,3±0,6,<br>Ligature+overload: 2,6±0,7,<br>Overload: 1,3±0,4,<br>Control: 1,1±0,5.<br>(224d ligature + 112d overload)	-	Histo. Mean value of buccal and lingual aspect of each implant	-	1,1±0,5	None
9. Tillmanns et al	1998	Ligature, submarginal	Cotton, braided retraction cord (GingiBraid, VanR Dental Products, Oxnard, CA)	NA (although type and brand mentioned)	Only if necessary at plaque control appointments	3m 6 dogs<br>6m 8 dogs	Ligatures on one side, continued plaque control on the other side of the mandible	No (reported in separate paper)	No (reported in separate paper)	DNA from deepest pocket of each implant at baseline and then monthky. Different thresholds used for extent of microbes. P.gingivalis P. intermedia Actinobacillus actinomycetemcomitans	Periapical at baseline and monthly	Ground sections	Bone height and subsequent vertical or horizontal bone loss.<br>Thickness of HA coating		90 (n=6) and 180 (n=8)) respectively after ligature placement	90 days - 6 dogs<br>HA ctrl: 0,87±0,44. Test: 2,64±2,76<br>TPS ctrl: 1,41±0,98. Test: 2,53±1,02<br>Ti-A ctrl: 1,57±0,75. Test: 2,90±1,41<br>180 days - 8 dogs<br>HA ctrl: 1,72±1,18. Test: 2,85±1,76<br>TPS ctrl: 1,46±0,90. Test: 2,21±0,94<br>Ti-A ctrl: 1,84±0,43. Test: 2,18±0,42	Fig 11, 12 in paper	Histo. Mesiodistal sections. Not specified whether an average of mesial and distal/ or greatest value was used.	-	-	3 implants lost, 1 of each kind.
10. Persson et al	1999	Ligature	Cotton	NA	New ligatures after 1m and 2m	3m	-	-	-	-	IO		-	-	90	Baseline<br>Average of 1,33±0,91, 1,60±0,47 and 1,42±0,76<br>30 days after ligature removal<br>Average of 1,47±0,91, 3,37±0,20 and 3,54±0,67	-	Periapical x-rays with individual film holders prepared for each animal and site. Not specified whehter a mean- or max value was used for each implant.	-	-	-

11. Deppe et al	2001	Ligature	Cotton floss	NA	No	3m	Ligatures around all implants	-	-	-	Periapical X-rays Long cone technique with custom made film holder	Ground sections – computer assisted histometry: Size of former bone defect and reappositioned bone	-	-	90	Group 1: 1,7±0,8 min 0,00 max 2,90<br>Group 2: 1,7±0,9 min 0,1 max 3,70<br>Group 3: 1,7±0,5 min 0,9 max 3,30<br>(all 3 groups identical in PI but differing in subsequent treatment)	-	X-ray peripical, customized holder. Mean value of mesial and distal aspect of each implant.	-	-	-
12. Persson et al.	2001a	Ligature	Cotton	NA	(1) 1 mo; (2) 2 mo	3m	-	-	-	-	IO	IC-BDc (mm), IC-PM (mm), PM-aJE (mm), aJE-CBI (mm), IC-CBI (mm), IC-BDh (mm)<br>Legends: implant shoulder (IC), bottom of the bone defect (BDc), the marginal level of bone in contact with the implant (CBI), the marginal portion of the periimplant mucosa (PM), the apical termination of the barrier epithelium (aJE), bottom of the defect (BDh)	-	-	90	Turned: 3.1±0.5 SLA: 3.2±0.3 (120 days)	-	X-ray	-	-	-
13. Shibutani et al	2000	Ligature, submarginal technique	Silk	NA	NA	12w	Ligaures around all implants. IM bisphosphonate injection (Pamidronate 0,6 mg/ kg IM every 3 days) vs control	-	-	-	Pariapical	-	-	-	84	Clinical bone loss (mm) in:<br><br>Pamidronate group 1,59±0,55<br><br>Control group 2,41±0,48	NA	Clinical measurement with periodontal probe on the buccal side at center of the implant	-	-	-
14. Shibli et al	2003	Ligature, submarginal and sutured in the peri-implant mucosa for retention	Cotton floss	NA	Further ligatures on top of old every 20d	60d	Ligatures around all implants	-	-	Before ligature placement and at 20d, 40d and 60d after ligature placement. Cultivation of various bacteria	Periapical, long cone technique at ligature placement and 20d, 40d and 60d after ligature placement	-	-	-	60	(1) cpTi 2,09±1,70<br><br>(2) TPS 1,70±1,52<br><br>(3) HA-coated 1,94±1,59<br><br>(4) hybrid turned+acid etched 1,62±1,32	NA	X-ray, periapical. Average of mesial and distal aspect of each implant	-	-	NA
15. Deppe et al	2004	Ligature	Cotton (floss)	NA	No	3m	Ligatures around all implants	Pressure-froced pocket probing in anesthezized animals	-	-	Periapical X-rays, long cone technique.	Ground sections – computer assisted histometry	-	-	90	Conventional X-ray: 2,92±0,51<br>Digital X-ray: 2,97±0,44	-	Conventional X-ray with indiviaual template	-	-	-

											Conventiona<br>l film VS<br>digital					Histology:<br>3,29±0,50		Digital X-<br>ray with<br>individual<br>template<br>Not specified<br>whether a<br>mean value<br>or max value<br>has been<br>used for each<br>implant			
16.<br>Martins<br>et al	2004	Ligature<br>(submarginal<br>position)<br>followed by 1<br>year of<br>supragingival<br>plaque control	Cotton<br>floss	NA	Further<br>ligatures at<br>20 day<br>intervals	60 days or<br>at 40%<br>bone loss	Ligatures<br>around all<br>implants	Pi, GR, BoP,<br>PPD, CAL,	Periotest	-	IO for<br>vertical and<br>horizontal<br>bone loss	-	-	-	Baseline to<br>20d<br>40d<br>60d	Baseline<br>TPS: 2,50 ± 0,61<br>HA: 2,01 ± 0,46<br>AE: 2,36 ± 0,54<br>cpTi: 2,40 ± 0,51<br>20 days<br>TPS: 3,85 ± 0,95<br>HA: 3,62 ± 0,29<br>AE: 3,64 ± 0,17<br>cpTi: 4,12 ± 0,72<br>40 days<br>TPS: 4,62 ± 0,90<br>HA: 4,65 ± 0,84<br>AE: 5,19 ± 0,51<br>cpTi: 5,20 ± 0,71<br>60 days<br>TPS: 6,00 ± 0,70<br>HA: 6,22 ± 0,50<br>AE: 6,06 ± 0,27<br>cpTi: 6,32 ± 0,00	Available in paper	Periapical x-<br>rays,<br>standardized<br>with<br>individual<br>bite-blocks.<br>Average of<br>mesial and<br>distal aspects<br>of each<br>implant.<br>Baseline<br>value greater<br>than 0 due to<br>ref. point<br>above the<br>bone crest.<br>Subtraction<br>of baseline<br>value from<br>reseptive<br>measurement<br>values will<br>give amount<br>of bone loss	-	-	None reported
17.<br>Martins<br>et al	2005	Ligature<br>(Submarginal<br>position) + 1 year<br>passive phase<br>with daily<br>cleaning	Cotton<br>floss	NA	Further<br>ligatures at<br>20 day<br>intervals	60 days	Ligatures<br>around all<br>implants	PD, CAL	Periotest	-	IO for<br>vertical and<br>horizontal<br>bone loss	-	-	-	Baseline to<br>20d<br>40d<br>60d<br>425d	Baseline<br>cpTi: 2,32 ± 0,53<br>TPS: 2,50 ± 0,61<br>HA: 2,01 ± 0,46<br>Acid: 2,36 ± 0,54<br>20 days<br>cpTI: 4,12 ± 0,72<br>TPS: 3,85 ± 0,95<br>HA: 3,62 ± 0,29<br>Acid: 3,64 ± 0,17<br>40 days<br>cpTI: 5,20 ± 0,71<br>TPS: 4,61 ± 0,90<br>HA: 4,65 ± 0,84<br>Acid: 5,19 ± 0,51<br>60 days<br>cpTI: 6,32 ± 0,33<br>TPS: 6,00 ± 0,70	Available in paper	Periapical x-<br>rays,<br>standardized<br>with<br>individual<br>bite-blocks.<br>Average of<br>mesial and<br>distal aspects<br>of each<br>implant.<br>Baseline<br>value greater<br>than 0 due to<br>ref. point<br>above the<br>bone crest.<br>Subtraction	-	-	None during<br>ligature phase,<br>17 during the<br>1 year follow<br>up

																HA: 6,22 ± 0,50<br>Acid: 6,06 ± 0,27		of baseline value from reseptive measurement values will give amount of bone loss			
18. Berglundh et al	2006	Ligature (sub-marginal position) +spontaneous accumulation	Cotton	NA	Every 2 weeks	4m with ligature and 5m with additional plaque accumulation	-	-	-	-	Periapical X-rays	LM analysis of ground sections. Greater amount of bone loss at the SLA sites than at Polished sites. (7,34 vs 5,95 mm)	-	-	120	SLA: 2,51± 0,55<br>Polished: 2,27±1,05		X-ray periapical, customized film holder. Mean values for each variable	Additional loss 5 months after ligature removal:<br>SLA: 1,12±1,07<br>Polished: 0,07±0,72	-	None reported
19. Shibli et al	2006	Ligature, submarginal and sutured in the peri-implant mucosa for retention	Cotton floss	NA	Further ligatures on top of old every 20d	90d	Ligatures around all implants	-	-	-	-	-	-	-	90	(1) Turned<br>2,43±0,96<br><br>(2) TPS<br>4,55±1,77<br><br>(3) Hybride<br>2,60±0,96<br><br>(4) Sandblasted with Ti-oxide<br>2,8±0,41	-	Intraoperative measurement with periodontal probe, after reflection of soft tissues. Mean value of 4 sites around each implant.	-	-	2 implants in 2 animals lost during ligature phase:<br>(1) cpTi (Turned) test group<br>(4) (Sanblasted Ti-oxide) control group
20. Albouy et al	2008	Ligature (sub-marginal position) +spontaneous accumulation	Cotton	NA	At weeks 3, 6, and 9	12w (40-50% boneloss) Plaque accumulation continued for 24 weeks after ligature removal	-				IO before ligature placement. At ligature removal. At 6, 14 and 24 weeks after ligature removal.	-	-	-	84	Baseline at ligature placement. Bone loss at ligature removal:<br>A: 3.53 ± 1,04<br>B: 4.10±0,63<br>C: 4,69±0,52<br>D: 3,58±0,37	-	X-ray peripical, customized holder. Mean value of mesial and distal aspect of each implant.	ADDITIONAL bone loss 24 weeks after ligature removal:<br>A: 1,84±1,41<br>B: 1,72±1,25<br>C: 1,55±0,68<br>D: 2,78± 1,91	-	A: 1 implant lost at week 26.<br>D: 1 lost at week 35.
21. Albouy et al	2012	Ligature (sub-marginal position) +spontaneous accumulation	Cotton	NA	Every 3 weeks	10w (12 according to Fig 1 text) Plaque accumulation continued for 26 weeks after ligature removal	-	-	-	-	IO periapical X-rays	LM analysis of ground sections. Localisation, vertical extension and area of ICT. Marginal bone position with abutment/fixture junction as reference	-	-	70	Marginal bone loss from IO X-rays:<br>At ligature removal (10 weeks):<br>A: 3,00 ± 0,44<br>B: 3,47 ± 0,45	-	X-ray peripical, customized holder. Mean value of mesial and distal aspect of each implant.	Additional bone loss 16 weeks after ligature removal:<br>A: 0,03 ± 0,50<br>B: 1,47 ± 0,65	-	None reported

22. López-Piriz et al	2012	Ligatures, submarginal technique	Cotton	NA	Replaced every 3w	3m	Ligatures around all implants	Assessment of plaque and inflammation weekly	-	-	Periapical radiographs	-	-	-	90	(Redcued variable) test<br>Distal test 1,33±1,56<br>Distal ctrl 3,47±3,01<br>Mesial test 2,04±3,72<br>Mesial ctrl 3,73±4,55	-	"X-rays, periapical with holder that allowed easy and predictable allignment. Mean of mesial and distal aspect"	-	-	1 case implant lost at abutment connection
23. Carcuac et al	2013	Ligature (Sub-marginal position) at implants and 2nd, 3rd and 4th premolars in left mandible	Cotton	NA	At weeks 3 and 6	10w + 26w continued plaque accumulation	Ligatures around all implants and teeth	-	-	-	Periapical X-rays with customized film-holder	LM analysis of groundsections,	Immunohistochemical analysis of paraffin sections.	-	70	"Turned: 2,69±0,57<br>Ti-Unite 3,14±0,69	-	X-ray peripical, customized holder. Mean value of mesial and distal aspect of each implant.	Additional loss 26 weeks after ligature removal:<br>Teeth: 0,00±0,53<br>A: -0,02±0,66<br>B: -1,34±1,19	-	None reported
24. Madi et al	2013	Ligature, submarginal position	Silk	NA	Replaced every 3w	4m with ligature + 5m spontaneous plaque accumulation after ligature removal	Ligatures around all implants	PPD, CAL, modified GI at 1,2,3,4,5, and 9 m with periodontal probe at fixed points marked on the abutments	-	-	Yes, long cone technique at baseline, 4m and 9m	-	-	-	120+150	Radiograph mesial:<br>(1) 1±0,6<br>(2) 1,1±0,7<br>(3) 1,1±0,5<br>(4) 1,7±0,6<br>Radiograph distal:<br>(1) 0,9±0,6<br>(2) 0,92±0,6<br>(3) 0,83±0,5<br>(4) 1,6±0,6	-	X-rays, periapical with custom-made long cone parallelling device. Mesial and distal aspects reported separately	Minor changes in CAL and PPD after 5 months spontaneous accumulation following ligature removal	-	none
25. Martinez et al	2014	Ligature (submarginal position)	Cotton	NA	Replaced every 3w	3m and 13w both mentioned	Ligatures around all implants	-	-	-	-	Plastic embedded ground sections Abutment fixture junction, Gingival margin, crestal bone margina, coronal position of bone to implant contact	See paper	-	90	Uncoated controls:<br>Lingual 3,2 ± 0,71<br>Buccal 2,8 ± 0,20<br>Coated mesial implant:<br>Lingual 2,8 ± 0,53<br>Buccal 2,5 ± 0,27<br>Coated distal implant:<br>Lingual 2,7 ± 0,56<br>Buccal 2,6 ± 0,19	-	Histo.<br>2 central bucco-lingual sections from each implant	-	-	One distal coated implant lost
26. Carcuac et al	2015	Ligature (sub-marginal position)	Cotton	NA	At weeks 3 and 6.	9w	Ligatures around all implants	-	-	4 weeks after ligature removal (hygiene reinstated at ligature removal) DNA-DNA hybridization tech	Periapical X-rays	-	-	-	63	TiOblast: 3,58±0,76<br>OsseoSpeed: 3,72±0,65<br>At-I: 3,73±0,47<br>TiUnite: 3,57±0,63	-	X-ray peripical, customized holder. Mean value of mesial and distal aspect of each implant.	-	-	None during the breakdown phase
27. Huang et al	2015	Ligature, sub-marginal	Cotton	NA	No	12w	Ligatures around all implats	General description	-	-	Yes but method not specified.	Assessment of supra-alveolar bone loss, infrabony defects, marginal bone loss.	Undecalcified ground sections in the bucco-lingual plane	-	84	"Distance from bone ridge to first bone-to-	Table 1 in paper	Histo + X-ray: Bucco-lingual	-	-	No losses

											Mesial and distal bone loss assessed radiographic ally					implant contact (Ridge-fBIC):<br>Crestal astra implant: 0,19±0,25<br>Crestal Bicon implant: 0,34±0,30<br>Subcrestal Astra: 1,32±0,48<br>Subcrestal Bicon: 0,95±0,39"		sections. Average from measurement s on buccal, lingual (histo) mesial and distal (X-ray) aspects. Bone ridge used as ref. point since implants were placed on both crestal and sub-crestal positions.			
28. Namgoo ng et al.	2015	Ligature	Stainless steel	NA	NP	23w	-	-	-	-	IO (5, 8, 12, 18, 23w), after GBR (4, 8, 12w)	PPDD, BRH, BRA, BIC	-	-	161	Turned: 2.4 ± 1.0 (XR) 2.7±1.2 (H) SA/HA: 2.7 ± 0.6 (XR) 3.5±1.2 (H) SA: 2.3 ± 1.0 (XR) 3.2±1.4 (H) (23 weeks) See fig 8	-	IO X-ray and histometric analysis	-	-	None reported
29. López-Piriz et al	2015	12w spontaneous accumulation followed by 10w of submarginal ligatures	Cotton	NA	Replaced every 3w	10w (preceded by 12 w spont. Accumula tion)	Same procedure on both sides.	-	-	CFU/ml and cultivation	Periapical radiographs	-	-	-	84+70	After spontaneous accumulation (84d):<br>Control 0,5±0,44<br>G3 0,45±0,39<br>ZnO35 0,8±0,77<br>G1n-Ag 1,2±0,47<br>Total bone loss after ligature (84d+70d):<br>Control 2,21 ± 0,46<br>G3 1,64±0,43<br>ZnO35 1,42±0,40<br>G1n-Ag 1,45±0,56<br>Bone loss during ligature phase<br>total bone loss - spontaneous accumulation loss	-	X-rays, periapical with holder that allowed easy and predictable allignment. Mean of mesial and distal aspect	-	-	1 G1n-Ag lost at sec surgery
30. Pirih et al	2015	Ligature	Silk	6-0	No	12w	Control animals without ligature	Gingival swelling	Bucco-lingual wiggling forces applied to implants after 4w	-	Micro-ct	Ground sections.	Ground sections	-	84	Distance from apical point of implant head to first bone contact, (4 lost ligature implants counted as 1 mm bone loss = total implant leangth):<br><br>Ligature group = n10<br>0,579±0,0490<br><br>Control group = n8	NA	Micro CT	-	-	4 ligature implants lost but included in the statistics.

																0,226±0,016					
31. Ishii et al	2016	Ligature	Dental floss	NA	-	90d	Same as experimental side	-	-	-	(1) Standardized dental radiographs (70kv, 15mA, 0,25s). (2) Micro-CT	Ground sections	-	-	90	(1)<br>UV-group: 2,0±0,5 mm<br>Controls: 2,7±0,4 mm	(2)<br>Area of bone resorption mm2<br>UV-group: 45,7±9,6<br>Controls: 64,4±10,6	(1) Dental x-rays Dental X-rays, standardized technique with silicone bite block mean of mesial and distal aspect of each implant<br>(2) Micro-CT	-	(1) 2,7±0,4	-
32. Godoy-Gallardo et al	2016	Ligature, submarginal	Silk	4-0	NA	2m	Ligatures around all implants	PPD, mucosal recession, keratinized ginigiva, CAL, PI, BoP	-	-	Periapical X-rays, micro-ct	Bone and tissue resorption with light microscopy. SEM also used.	Ground sections	-	30, 60	At ligature placement<br>Ti_Ag: 2,9±0,6<br>Ti_TSP: 2,8±0,5<br>Ti: 3,0±0,6<br><br>30 days<br>Ti_Ag: 3,5±0,4<br>Ti_TSP: 3,6±0,5<br>Ti: 3,9±0,7<br><br>60 days<br>Ti_Ag: 4,1±0,5<br>Ti_TSP: 4,0±0,5<br>Ti: 4,6±0,7		X-ray, periapical, standardized mean of mesial and distal aspect	-	-	No losses
33. Park et al.	2017	Ligature	Braided cotton	1.58 mm	Additional ligature every 4 weeks (no exchange)	4m	-	IS-BD	-	-	IO	(1) the mineralized tissue area (mm2), including the newly formed bone and bone graft material; (2) coronal bone loss (IS-BD) from the implant shoulder (IS) to the bottom of the defect (BD); (3) defect depth (BC-BD) from the bone crest (BC) to BD; (4) the re-osseointegration height (mm) as the most coronal BIC level from BD; (5) vertical bone fill (%) as the ratio of the re-osseointegration height to IS-BD; (6) total perimeter of BIC (mm) within the re-osseointegrated bone, and (7) the BIC ratio (%) within re-osseointegrated bone as the ratio of (1) the mineralized	-	-	120	***IS-BD (clinical)***<br>Immediate model<br>3.88±0.99 (mesial)<br>3.88±0.99 (distal)<br>5.38±1.30 (buccal)<br>3.38±0.52 (lingual)<br>Conventional model<br>3.50±0.53 (mesial)<br>3.13±0.35 (distal)<br>4.00±0.53 (buccal)<br>3.13±0.35 (lingual)<br>***IS-BD (histological)***<br>Immediate model<br>6.02±1.20 (buccal)<br>4.41±1.07 (lingual)<br>Conventional model<br>4.34±0.86 (buccal)<br>3.81±0.61 (lingual)<br>***BC-BD (histological)***<br>Immediate model<br>0.01±0.00 (buccal)<br>1.06±0.73 (lingual)	-	Histometric analysis	-	-	None reported

												tissue area (mm2), including the newly formed bone and bone graft material; (2) IS-BD from the IS to the BD; (3) BC-BD from the BC to BD; (4) the re-osseointegration height (mm) as the most coronal BIC level from BD; (5) vertical bone fill (%) as the ratio of the re-osseointegration height to IS-BD; (6) total perimeter of BIC (mm) within the re-osseointegrated bone, and (7) the BIC ratio (%) within re-osseointegrated bone as the ratio of the total perimeter of the bone contact to the whole thread perimeter (mm) between BD and the first BIC position				Conventional model 0.11±0.20 (buccal) 1.20±0.58 (lingual)					
34. Lin et al	2017	Ligature – Stainless steel ligature wrapped 6 turns around the implant neck and forced as deeply into the pocket as possible	Stainless steel	0,010 inch	No	12w	Ligatures around all implants	PPD at 4 aspects per tooth baseline, ligature removal and 4w after ligature removal	-	-	Standardized periapical radiographs at 3w and 12w	-	-	-	21, 84	21d (mm) 1,6±0,6<br><br>84d 4,0±0,8	Width of bone defect mm (linear distance between the 2 peaks of the saucer-shaped defect, minus the diameter of the implant):<br><br>3w: 4,9±1,2<br><br>12w: 6,8±0,8	X-ray, periapical, film holder connected to tube. Average of mesial and distal values	-	-	No loose or lost implants at ligature removal
35. Koutouzis et al	2017	Bacterial inoculation with P. gingivalis, Treponema Denticola, Tannerella forsythia by means of gingival lavage	-	-	-	24 inoculations during a 6w period	6/12 animals were sham inoculated	-	-	Yes, 16S ribosomal RNA	Micro-ct	-	-	-	42	Infected (n= 4 implants): 0,80±0,72<br>+2 infected rats had both implants failed<br>Sham-infected (n= 3 implants): 0,48±0,13		Micro-CT. Distance between implant platform to first BIC.			Majority of implants in both groups were excluded before study end, for reasons outside the treatment, such as aspiration: Three rats with 4 implants in the infection group and two rats with 3 implants in the sham-infection

group were analyzed

NA = not available; X = not investigated; Pi = plaque index; GR = gingival redness; BoP = Bleeding in probing; PPD = pocket probing depth; CAL = clinical attachment level; TPS = titanium plasma sprayed; HA = hydroxyapatite, U = ~~uncoated~~ turned/machined; IO – intraoral; ICT = Infiltrated connective tissue; MBL – marginal bone level
LM: Light microscope, GI = Gingival index
